# Local US officials’ views on the impacts and governance of AI: Evidence from 2022 and 2023 survey waves

**DOI:** 10.1371/journal.pone.0332919

**Published:** 2025-10-06

**Authors:** Sophia Hatz, Noemi Dreksler, Kevin Wei, Baobao Zhang

**Affiliations:** 1 Department of Peace and Conflict Research, Uppsala University, Uppsala, Sweden; 2 Centre for the Governance of AI, Oxford, United Kingdom; 3 Harvard Law School, Harvard University, Cambridge, Massachusetts, United States of America; 4 Political Science Department, Maxwell School of Citizenship and Public Affairs, Syracuse University, Syracuse, New York, United States of America; A’Sharqiyah University, OMAN

## Abstract

This paper presents a survey of local US policymakers’ views on the future impact and regulation of AI. Our survey provides insight into US policymakers’ expectations regarding the effects of AI on local communities and the nation, as well as their attitudes towards specific regulatory policies. Conducted in two waves (2022 and 2023), the survey captures attitudinal changes within the six months following the public release of ChatGPT and the subsequent surge in public awareness of AI. Local policymakers express a mix of concern, optimism, and uncertainty about AI’s impacts, anticipating significant societal risks such as increased surveillance, misinformation, and political polarization, alongside potential benefits in innovation and infrastructure. Many also report feeling underprepared and inadequately informed to make AI-related decisions. On regulation, a majority of policymakers support government oversight and favor specific policies addressing issues such as data privacy, AI-related unemployment, and AI safety and fairness. Democrats show stronger and more consistent support for regulation than Republicans, but the latter experienced a notable shift towards majority support between 2022 and 2023. Our study contributes to understanding the perspectives of local policymakers—key players in shaping state and federal AI legislation—by capturing evolving attitudes, partisan dynamics, and their implications for policy formation. The findings highlight the need for capacity-building initiatives and bi-partisan coordination to mitigate policy fragmentation and build a cohesive framework for AI governance in the US.

## Introduction

The release of ChatGPT in November 2022 drew the world’s attention to Artificial Intelligence (AI), demonstrating the potential of AI to impact nearly all aspects of society. Both expectations of vast benefits across areas such as the economy, healthcare, education, and national security, and concerns about risks such as discrimination, job loss, and authoritarianism, have come to the forefront of public discourse.

As the far-reaching impacts of AI materialize, governments are increasingly considering regulating AI. In the years following the launch of ChatGPT, several concrete AI regulations emerged [1]. The European Union advanced landmark policy action with the EU AI Act: a legislative framework that governs AI development, marketing, and use within the EU, approved by the European Parliament in 2024 [2,3]. In the US, there has been a surge in regulatory policy on AI at the national, state, and local level. In October 2023, US President Biden issued an Executive Order on the “Safe, Secure, and Trustworthy Development and Use of AI,” including new standards for safety and security and provisions to protect citizens and workers. AI became a focal topic for state legislatures in 2023, with a massive increase in the number of proposed AI-related bills. City and county governments have also moved forward, adopting AI technologies in city services and enacting their own AI policies. As AI continues to advance rapidly, understanding how policymakers view and plan to govern this technology is increasingly critical.

This paper examines US policymakers’ perspectives on AI’s future impact and regulation, focusing specifically on local elected officials. Our survey provides insight into the expectations of local US policymakers about the future effects of AI on local communities and the nation, as well as their attitudes toward specific regulatory policies. We analyze how views on AI vary between Democrats and Republicans, as well as by gender and level of education. In addition, we conducted our survey in two waves, May/June 2022 (*n* = 524) and May/June 2023 (*n* = 504), allowing us to assess how attitudes changed from the six months before to the six months after the public release of ChatGPT [4], during a period that witnessed a substantial increase in public awareness of AI [1,5].

In general, local policymakers express a mixture of concern, optimism, and uncertainty about the impacts of AI over 2025–2050 in their local community and the broader country. A majority of local policymakers anticipate significant societal risks from AI over the next decades, including increased surveillance, misinformation, political polarization, decreased data security, and threats to US democracy. Some also foresee positive impacts, particularly in innovation and transportation/infrastructure. Views on AI’s future economic impacts are more measured, with most expecting negative impacts on jobs and inequality, but mixed expectations about the US economy. Notably, policymakers also express a great deal of uncertainty, particularly around broader impacts such as international conflicts, the probability of great power war, bias and discrimination, and the impact on the US economy. Also reflecting this uncertainty, local policymakers feel under-prepared for AI governance, believing they are unlikely to make AI-related decisions in the next few years, and reporting they feel inadequately informed to make such decisions.

In regard to the governance of AI, we asked respondents whether they thought AI should be regulated, and whether they thought a range of AI policies would be beneficial for the country in the years 2025–2050. A majority of local policymakers support government regulation and oversight. Further, policymakers are in favor of a number of policies that address specific AI issues such as data privacy, AI-related unemployment, and AI safety and fairness. UBI stands out as the only AI policy proposal facing strong opposition.

However, in examining support for AI regulation between Democrats and Republicans, we find substantial differences. Democrats are generally more in favor of government regulation than Republicans, and consistently show greater support for policies addressing specific AI use cases and issues. This includes policies related to AI-driven unemployment, wage decline, regulation that ensures deployed AI systems are safe, robust, and fair, AI-use in judicial decisions, AI use in hiring, immigration reform for AI developers, and company use of robots.

Tracking changes in attitudes towards AI over the 2022–2023 time period, we observe a significant increase in support for government regulation of AI in the six months after as compared to six months before ChatGPT release. Although support increased among both Democrats and Republicans, the change was much greater among Republicans, who shifted to majority support in 2023.

Understanding local officials’ views on AI is important for several reasons. First, due to the decentralized nature of US policymaking, local officials on the county-, municipal- and township-level play important roles in shaping state legislation. For example, AI guidelines introduced by the city of Seattle were influential in shaping Washington state law [6]. Second, in the absence of federal-level comprehensive AI law, US AI policy is largely being shaped by states, particularly since many states are controlled by one political party [7, p. 7]. Our survey of local US officials thus provides insight into expectations and attitudes that will shape how AI is regulated in the US.

Our study expands on earlier survey research in a number of ways. First, our survey of local US policymakers is thematically broad, measuring expectations of future risks and benefits, as well as attitudes towards government regulation and a battery of potential policies. While existing survey research provides comprehensive understanding of US public opinion on AI [8], and prior surveys of US officials provide insights into attitudes on specific issues such as autonomous vehicles [9], we lack a broad mapping of the views of local US political elite.

Second, our two survey waves—fielded in 2022 and 2023—repeated the same questions, allowing us to capture how policymakers’ views changed over this time period. This time period spans sudden and significant changes in AI, including the public releases of OpenAI’s ChatGPT and GPT-4, Midjourney, Stable Diffusion, Microsoft’s Bing AI Chat, and Google Bard [4,10–14]. Public awareness of AI and concern about its risks also increased significantly over this period [15]. Third, we focus on the differences in views on AI across Democrats and Republicans, as well as changes in partisan differences over the 2022–2023 time period.

Four key findings have clear implications for the trajectory of AI regulation in the US. First, we observed a significant increase in local policymakers’ support for AI regulation in 2023. While we cannot rule out other simultaneous influences during 2022–23, many analysts attribute the surge in AI regulation globally, nationally, and in the US to the release of ChatGPT in 2022 [1, p. 17] [5,16]. Given that advances in AI technology tend to be sudden and significant [17], it’s likely that big changes such as those in 2022–23 will occur again if we accept that substantial jumps in support for AI regulation are associated sudden and significant AI advancements.

Second, we observed polarization in views on AI regulation across party lines, with Republicans consistently showing less support for government regulation, as well as for policies addressing specific AI challenges like unemployment, safety, and fairness. Interestingly, however, this polarization was stronger in regard to policy preferences; Democrats and Republicans express more similar views on the impacts of AI. We believe this has important implications both for public opinion formation and for policy adoption. Some research suggests that on polarized issues, the public tends to form opinions in line with the political elite who share their partisanship [18,19], relying less on substantive arguments about the issue [20]. Further, polarization along party lines can have implications for state policy. Divergent state-level policies may result in “patchwork” AI legislation across the US, which could generate uncertainty and complications for companies, workers and consumers who operate across state lines [16].

Third, we also observed some indication that partisan differences are decreasing, as a majority of Republican local policymakers now support government regulation. This is likely to support emerging bi-partisan efforts towards comprehensive AI legislation, such as the SAFE Innovation Framework proposed by Senate Majority Leader Chuck Schumer [21].

Finally, our finding that local officials feel uncertain about AI’s impacts and are unprepared to make AI-related policy decisions highlights the need for both capacity-building and further research. State and local legislators have begun tackling this issue through capacity-building laws establishing working groups tasked with studying AI [7, p. 12–13]. Future survey research could support these efforts by evaluating the specific areas where policymakers may feel least informed or prepared, such as the technical aspects of AI, its economic implications, or its potential role in international relations, and tracking changes in competencies over time.

## Literature review

### US AI policy during 2022–2023

Over the past decade, governments have grown increasingly aware of the need to regulate AI in order to address risks while maximizing the benefits. Concrete regulatory policies have emerged in the past five years, with significant policy advances in 2023, in the year following the launch of ChatGPT [1].

In particular, the European Union advanced landmark policy action with the EU AI Act: a legislative framework which governs AI development, marketing and use within the EU [3]. This is the world’s most comprehensive legislation on AI. Other notable policy actions in 2023 include the UK’s AI Safety Summit and release of a government white paper on AI regulation [22,23], as well as China’s announcement of an “Artificial Intelligence Law” on its legislative agenda [5].

In the US, President Biden issued in 2023 an Executive Order on the “Safe, Secure, and Trustworthy Development and Use of AI,” including new standards for safety and security and provisions to protect citizens and workers. AI also became a high-priority issue in Congress, with a substantial jump in the number of proposed AI-related bills, rising from 88 in 2022 to 181 in 2023 [1, p. 17]. Some bills –primarily focused on government uses of AI– have been voted on and have passed through committee stages [21]. Yet, the US does not yet have “comprehensive” legislation on AI: law which imposes broad new consumer protections and company requirements for AI [7, p. 13]. The biggest obstacle to comprehensive legislation is Congress; where a lack of consensus makes passing legislation unlikely [21,24].

In the absence of congressional legislation on AI, there has been a surge in activity at the state-level. State legislators introduced 191 AI-related bills in 2023; a 440% increase since 2022 [6]. 14 of these bills were enacted into law in 2023. Many of these laws are issue-specific, targeting specific AI use cases and concerns. The range of issues varies widely, including government use, elections, privacy and pornography [7, p. 12–13]. For example, Arizona, Minnesota and Texas passed laws concerning AI in law enforcement. Another six states passed privacy reforms limiting the use of AI in profiling. Three states passed laws targeting generative AI and deepfakes in political advertising. States also enacted capacity-building laws which set up working groups or task forces to study AI, conduct inventories or develop regulatory frameworks [7, p. 12–13]; [6,25].

At the local level, municipalities and county governments have also moved forward, enacting their own policies on AI. Most of these policies, such as in Boston, Santa Cruz County and Grove City, focus on government uses of AI [26,27]. These cities have incorporated AI technologies in city services, serving as pioneers in the anticipation of risks, ethical considerations and necessary regulation [25].

Overall, there has been an enormous increase in federal, state and local government attention to AI regulation in 2023; it has become the “hot topic” in technology policy. There is also great heterogeneity in the AI laws passed, with laws targeting concerns ranging from profiling in automated decisions to deep fakes in political advertising. This highlights the diversity of perspectives on AI in terms of perceived impacts, risks and policy priorities.

### Survey research on AI impacts and policy

Survey research provides insight into the drivers of AI policy by examining attitudes such as expectations of risks and benefits and preferences for regulation. Prior survey research on AI tends to target one of several different audiences: the general public, AI experts, or elite audiences such as CEOs, academics and policymakers.

Surveys of the general US public measure a wide variety of themes, including general awareness and use of AI, trust in AI, support for specific applications, views on AI labs, attitudes towards regulation, expectations of achieving intelligence milestones and concern about risks. Citizens tend to be optimistic about certain uses of AI, particularly criminal justice applications [28,29] and some medical uses [15,29]. They also express concern about risks such as economic harm/job loss [28,30,31] and existential threats [32–34]. A number of surveys asked whether citizens are overall more excited or more concerned about AI, and the results consistently show that concern outweighs excitement [28,29,35–37]. Among the surveys that include a category corresponding to being equally excited and concerned, however, a substantial proportion of respondents fall into this middle ground, reflecting ambivalence or a balanced view of AI’s potential benefits and risks [28,36,38]. From a few public opinion surveys which repeated the same questions in subsequent waves, concerns about risks appear to have increased over the past few years [35,39]. When it comes to the regulation of AI, polls show the majority of the public is in favor [15,29,34,40–42].

A second category is surveys of AI experts such as scientists, researchers and conference participants. In the most recent and largest survey, AI researchers assign non-negligible probabilities to both extremely good outcomes and extremely bad outcomes [43]. Specific risks of high concern include misinformation, increasing inequality and authoritarian control [43, p. 12]; [34, p. 5]. When it comes to the regulation of AI and the implementation of AI safety measures, AI experts express broad support [33,34,44]. One study comparing US voters and AI experts finds that while both groups favor AI regulation, the public considers societal-scale risks both more likely and more impactful [34].

A final category of surveys are those that target elite audiences such as corporate managers, CEOs, entrepreneurs and academics. These surveys tend to focus on risks and opportunities, particularly in reference to business or research. Elite respondents are optimistic about potential benefits of AI, such as advances in health care and education [45], improvements in research processes [46] and gains in efficiency and profitability [47,48]. The risks they express most concern about include misinformation, inaccuracy and cybersecurity [45,46,48–50].

While surveys of the public, AI experts and elite audiences are becoming regular, there have been relatively fewer studies of political elites. To our knowledge, the only prior study of US policymaker opinion on AI is Horowitz and Khan’s survey of 690 US local officials [9], focusing on attitudes towards the adoption of AI technology such as autonomous vehicles and autonomous surgery. As in surveys of the public, local US officials’ express both optimism and pessimism, with approval of AI varying widely depending on the specific use case.

## Materials and methods

In this section, we present methodological details about our survey sample, survey questionnaire, and analysis. A pre-analysis plan was filed prior to analyzing survey results [51]. The pre-analysis plan (https://osf.io/k2efj), and the deviations from the pre-analysis plan (https://osf.io/5kb23) can be found on OSF.

### Sample

We conducted two national surveys of local elected officials in the United States. Data was collected in two independent waves by CivicPulse [52], a non-profit organization that surveys local and state elected officials for academic and public policy research. CivicPulse maintains a list of U.S. government officials for whom contact information is publicly available, and the list is updated every year; respondents are randomly sampled from this list [53]. Specifically, in the context of our survey, CivicPulse collected random samples of US elected officials serving counties, municipalities, and townships with populations of over 1,000 residents. The first survey wave was conducted April 1–May 29 2022 (about six months before ChatGPT release) and the second May 9–June 20 2023 (about six months after ChatGPT release). CivicPulse also collected incomplete responses in 2022 but not in 2023 (*n* = 46); these incomplete responses are not reported below and were excluded from our analysis.

**Fig 1:**
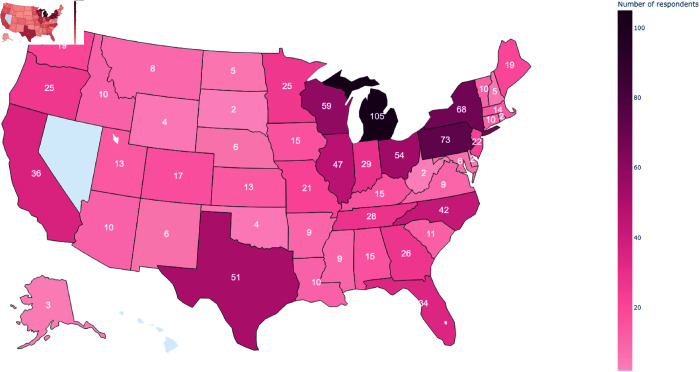
Number of respondents per US state. The figure shows unweighted absolute frequencies across both survey waves; gray regions represent states from which we received no survey responses.

We obtained a total of 1028 complete survey responses across both waves, with 524 and 504 responses from 2022 and 2023, respectively. The sample consisted of county-level officials, municipal-level officials, and township-level officials from across the country, with respondents’ government levels contained in Table 1. Respondents’ party identification are contained in Table 2. Personal demographics of the respondents (gender, race, age, and education level) are contained in Table 3. Number of respondents per state are visualized in Fig 1. Sample representativeness statistics for each sample are included in S8 Sample Representativeness. All tables and figures in this section are for unweighted data, using the pooled sample (i.e., combining the 2022 and 2023 samples).

**Table 1:** Frequency table of respondents’ level of government. The table shows unweighted absolute and relative frequencies across both survey waves.

	Count			Percentage		
	2022	2023	Total	2022	2023	Total
**Government Level**						
Township	125	111	236	23.85%	22.02%	22.96%
Municipality	321	324	645	61.26%	64.29%	62.74%
County	78	69	147	14.89%	13.69%	14.30%

**Table 2:** Frequency table of respondents’ political party identification.^a^

	Count			Percentage		
	2022	2023	Total	2022	2023	Total
Democrat	197	174	371	37.81%	34.73%	36.30%
Independent	38	31	69	7.29%	6.19%	6.75%
Republican	286	296	582	54.89%	59.08%	56.95%

^a^ The table shows unweighted absolute and relative frequencies across both survey waves. We report transformed party data that is used for our regression model. Definitions of this variable and the transformation are described in S2 Variable Definitions.

**Table 3:** Absolute and relative frequencies of respondents’ personal demographics, totals and segmented by year.^b^

	Count			Percentage		
	2022	2023	Total	2022	2023	Total
**Gender**						
Women	149	89	238	29.33%	24.86%	27.48%
Men	359	262	621	70.67%	73.18%	71.71%
Other (Self-Described)	0	7	7	0.00%	1.96%	0.81%
**Race** ^a^						
White	443	302	745	86.02%	87.79%	86.73%
Non-White	72	42	114	13.98%	12.21%	13.27%
**Median Age (Birth Year Range)^b^**						
21 (2001 - 2005)	1	0	1	0.19%	0.00%	0.12%
26 (1996 - 2000)	0	1	1	0.00%	0.30%	0.12%
31 (1991 - 1995)	2	5	7	0.39%	1.50%	0.83%
36 (1986 - 1990)	12	9	21	2.33%	2.70%	2.48%
41 (1981 - 1985)	19	8	27	3.69%	2.40%	3.18%
46 (1976 - 1980)	36	23	59	6.99%	6.91%	6.96%
51 (1971 - 1975)	32	31	63	6.21%	9.31%	7.43%
56 (1966 - 1970)	43	33	76	8.35%	9.91%	8.96%
61 (1961 - 1965)	44	49	93	8.54%	14.71%	10.97%
66 (1956 - 1960)	89	57	146	17.28%	17.12%	17.22%
71 (1951 - 1955)	107	57	164	20.78%	17.12%	19.34%
76 (1946 - 1950)	78	41	119	15.15%	12.31%	14.03%
81 (1941 - 1945)	35	13	48	6.80%	3.90%	5.66%
86 (1936 - 1940)	15	3	18	2.91%	0.90%	2.12%
91 (1931 - 1935)	2	1	3	0.39%	0.30%	0.35%
96 (1926 - 1930)	0	1	1	0.00%	0.30%	0.12%
104 (1920 or earlier)	0	1	1	0.00%	0.30%	0.12%
**Education Level**						
Less than high school	2	1	3	0.38%	0.20%	0.29%
High school graduate	40	45	85	7.68%	9.04%	8.34%
Technical/trade school	26	33	59	4.99%	6.63%	5.79%
Some college	97	89	186	18.62%	17.87%	18.25%
College graduate	131	140	271	25.14%	28.11%	26.59%
Some graduate school	38	38	76	7.29%	7.63%	7.46%
Graduate degree	187	152	339	35.89%	30.52%	33.27%

^a^CivicPulse collected data for sub-groups beyond ‘non-white’ in 2022 but not in 2023, so we report only white/non-white.

^b^CivicPulse collected only birth year ranges, and we impute ages by subtracting median birth year in each bin from 2024. These imputed ages are used in the regression analysis below and are treated as a continuous variable.

^c^The table shows unweighted absolute and relative frequencies across both survey waves.

### Survey questions

The main survey questions consisted of five sets of Likert-scale questions about AI impacts and governance; questions were identical in both waves of the survey.

Question Sets 1 through 3 (QS1–QS3) measured expectations of future AI impacts: QS1 and QS2 consisted of questions about the effects of AI on respondents’ local communities, and QS3 asked about the effects of AI beyond respondents’ local communities. Responses for QS1 and QS3 were measured on a 5-point Likert response scale representing respondents’ expectations of the expectations of AI, from strongly decrease (-2) to strongly increase (2); the 5-point Likert response scale for QS2 was similar but presented in terms of strongly worsen (-2) to strongly improve (2). Respondents were permitted to select “I don’t know” (“IDK”) for QS1–2 but not for QS3.

Question 4.1 (Q4.1) and Question Set 4 (QS4) measured attitudes towards AI governance. Q4.1 measured respondents’ support for government regulation of AI in general (Q4.1). QS4 measured respondents’ beliefs about whether specific federal policies on AI would benefit the United States. The 5-point Likert response scale in Q4.1 and QS4 represented respondents’ agreement with particular policy positions or statements, from strongly disagree (-2) to strongly agree (2). Respondents were not permitted to select “I don’t know” (“IDK”) for Q4.1 or QS4.

Q4.2 measured respondents’ respondents’ beliefs on long-term AI impacts until 2021, asking whether they expected overall positive or negative benefits. This question was also the outcome variable in a survey experiment, but as the experiment is not central to this paper, we describe the survey experiment design and results in S7 Survey Experiment.

The survey questions in QS1–QS4 (excluding Q4.1 and Q4.2) were presented to the respondents in grid format, and the survey incorporated a planned missing design [54,55]. Respondents were randomly presented with three of six questions in QS1, three of five questions in QS2, four of six questions in QS3, and five of fifteen questions in QS4. In practice, the proportion of missing data from the survey design fell in the range of 39–46% for QS1, 32–38% for QS2, 25–30% for QS3, and 66–69% for QS4.

Question Set 5 (QS5) consisted of two questions (Q5.1, Q5.2) related to policymakers’ preparedness for AI-related policy decisions. QS5.1 asked respondents to indicate the likelihood that their local government would make decisions about AI “in the next few years,” and QS5.2 asked respondents to assess whether they felt “adequately prepared to” make decisions about AI. The Likert scale for Q5.1 contained six options, asking respondents to rate probabilities from 0%, 10%, 25%, 75%, 90%, and 100%; the response scale for Q5.2 was the same as for QS4 and Q4.1. Respondents were permitted to select “I don’t know” (“IDK”) for Q5.1 but not for Q5.2.

Finally, the survey included collected demographic information on gender, age, education level, race, political ideology, and political party. CivicPulse then joined the data on respondents’ level of government, geographical location (US state), and US census data.

The dataset also included survey weights: both pooled and unpooled weights were calculated by CivicPulse using a post-stratification raking procedure per [56]. Weights were calculated using Census data for the population of the local government, the proportion of the local population 25 and older with a 4-year college degree, and the county’s proportional votes for President Biden in 2020 (see S3 Survey Text).

Aside from variables constructed from individual survey questions, we constructed several indices which aggregate responses across multiple survey questions. In particular, we create a policy agreement index (average across QS4 and Q4.1); positive impacts index (average of all impacts in QS1–3 which correspond to societal benefits); positive impacts for local community index (average of local community impacts in QS1–3 which correspond to societal benefits); personal well-being and community index (average of impacts on mental health, physical health and quality of life in QS1–3); and progress and innovation index (average of transportation and infrastructure items in QS1–3). A full list of constructed indices and their component items is included in S4 Indices Definitions.

S3 Survey Text contains the complete survey text.

### Analysis

We conducted our analysis of the survey data according to the filed pre-analysis plan [51]. As specified in the pre-analysis plan, we first generate summary statistics describing respondent demographics, expectations of AI impacts and attitudes towards AI governance, pooling the two survey waves. To examine how expectations of AI impacts and attitudes towards AI governance vary across respondent subgroups and over the 2022–2023 time period, we specify in Eq 1 a survey-weighted linear regression model [57]:

$$y_{i}=gender+age+edu+race+party+year_{2023}+party*year_{2023}\;+gov_{municipality}+gov_{county}+college+pop+Biden\;+1_{y_{i}\in \{Indices\backslash Index_{Policy\;Agreement}\}}*policy_{regai}$$ (1)

where $y_{i}\in \{QS1\;-\;4,Q4.1,Indices\}$ represents the outcome variable, which includes the questions on impacts and governance as well as the constructed indices.

To assess group differences we include the respondent-level demographic variables *gender*, *age*, *education*, *race* and *party*. *Party* indicates political party identification, constructed by combining questions on ideology and self-identified political party affiliations (details in S2 Variable Definitions). To assess changes over time, we include the dummy variable *Year*_2023_, which indicates the survey wave (2022 vs. 2023). We also include an interaction term for the combined effect of year and party identification: party*year_2023_.

We include five control variables. $Gov_{municipality}$ and $gov_{county}$ are dummy variables for the level of government of the respondent. *College* is the proportion of residents in each respondent’s geographic unit who are 25 years or older and who have completed a 4-year, post-secondary degree, based on 2015–2019 US Census data and binned in terciles. *Pop* is the total number of residents in each respondent’s geographic unit, based on 2015–2019 US Census data and binned in terciles. *Biden* is the proportion of votes by county for Joe Biden in the 2020 Presidential election, binned in terciles.

$1_{y_{i}\in \{Indices\backslash Index_{Policy\;Agreement}\}}$ is an indicator variable for whether *y*_*i*_ is any index except the policy agreement index, and *policy*_*regai*_ is Q4.1 (which is excluded from the regression equation for the policy agreement index as it is an element of that index).

Missing data for variables in the regression analysis was imputed using multiple imputation by chained equations (MICE) [58–60], which is appropriate for planned missingness designs [61–63]. The MICE model specification contained all questions in QS1–4, and all demographic information as well as survey weights (described in Survey questions). 120 imputed datasets were generated with predictive mean matching (PMM) and run for 200 iterations each. All IDK responses in QS1–4 were recoded to 0 (neutral). Running the regressions after coding the IDK responses as missing (and generating them using MICE) increased the number of statistically-adjusted regression coefficients that were significant at the *p* < 0.05 level, but all significant coefficients under the original coding scheme remained significant at the *p* < 0.05 level. Analysis on the dataset where IDKs were treated as missing, and were then imputed, are presented in S6 Alternative Regression Results. We also generated indices based on the survey questions, which are described in S4 Indices Definitions and on which we fit the regression model above. Note that imputed data and survey weights are only used for regression analysis, so regression results are based on both weighted and contain include data imputed from the original CivicPulse sample. All descriptive statistics and figures (e.g., in Sample and S1 Additional figures) are generated using only original data from the CivicPulse sample (i.e., they do not contain imputed data and are unweighted).

Detailed definitions and coding for all variables are contained in S2 Variable Definitions. Statistical corrections using the Benjamini-Hochberg method were applied to all regression coefficients. The statistical analysis was conducted in R and Python.

## Ethics statement

This study was approved by the Syracuse University Institutional Review Board (#22-045). Informed consent was collected by CivicPulse at the start of the surveys, ensuring only respondents who agreed to participate entered the sample.

## Results

In this section, we present summary statistics and the results of our regression analyses. We begin by describing the distribution of responses in three categories of questions: 1) expectations of the impacts of AI on local communities and the broader country; 2) attitudes towards specific AI policies and AI regulation; and 3) preparedness for AI-related policy decisions. We then examine group differences and changes over time, presenting select regression results per the model specified in Analysis. Full regression results, descriptive statistics, and additional figures are included in S5 Full Regression Results and S1 Additional figures.

### Expectations of the impacts of AI

**Fig 2:**
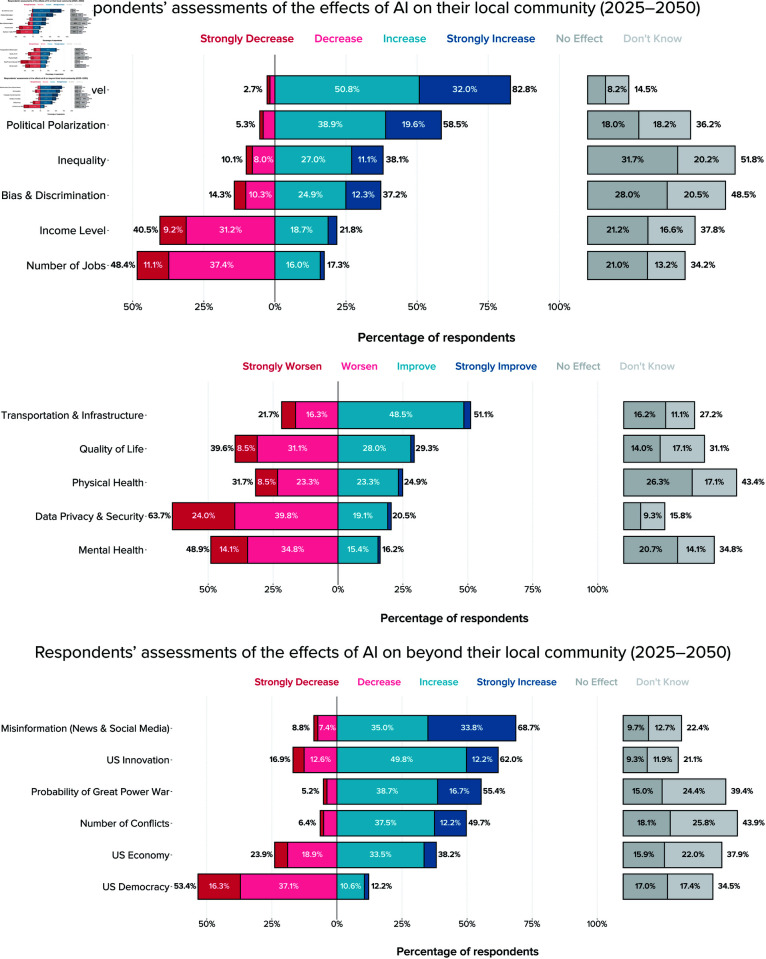
Local US officials’ expectations of the impacts of AI between 2025 and 2050. The figure shows unweighted relative frequencies across both survey waves. In all figures throughout, labels for bars containing relative frequencies of less than 7.0% are hidden for readability.

In regard to the impacts of AI over 2025–2050 in their local community and the broader country, we found that most local policymakers anticipate AI will create significant societal risks over the next decades (Fig 2). A majority of respondents indicated that they somewhat or strongly agreed that AI will increase surveillance levels (83%), increase misinformation (69%), decrease data security (64%), increase political polarization (59%), increase the probability of great power war (56%), and decrease the strength of US democracy (53%). A plurality of respondents also expect other negative effects such as worsening mental health outcomes (49%), increased international conflicts (49%), and decreased numbers of jobs (48%).

Although these results show that respondents are pessimistic about many of the societal impacts of AI, Fig 2 also shows that they expect positive effects in two areas: rates of innovation in the US (62%) and local transportation/infrastructure (51%).

Local policymakers express measured and mixed views on the economic impacts of AI in their local community. Along several economic indicators, greater proportions of local officials expect decreases rather than increases, including jobs (48% vs. 17%), income levels (41% vs. 22%), and inequality (38% vs. 10%). In terms of broader economic impacts, there are mixed expectations: 38% expect the US economy to grow thanks to AI, 24% expect it to decline, and 16% believe it will have no effect.

Respondents also express some uncertainty about the future impacts of AI, with high proportions of both “I don’t know” (“IDK”) and “neither agree nor disagree” (“neutral”) responses, though the latter may not always reflect uncertainty. The highest rates of “I don’t know” responses are for questions about international conflicts (26%) and the probability of great power war (24%). Around a fifth of respondents indicated “I don’t know” with regard to the local impacts of AI on inequality (20%), bias and discrimination (21%) and about the broader impact of AI on the US economy (22%). In addition, a few questions have high proportions of neutral responses, most notably inequality (32%), bias and discrimination (28%), and physical health (26%).

### Attitudes towards governance of AI

To measure attitudes toward the governance of AI, we asked respondents whether they thought AI should be regulated, and whether they thought a range of AI policies would be beneficial for the country in response to AI in the years 2025–2050.

Overall, a majority of local policymakers express support for government regulation: 64% strongly or somewhat agree with the statement that AI should be regulated by the government, with 19% IDK and only 16% disagreeing.

**Fig 3:**
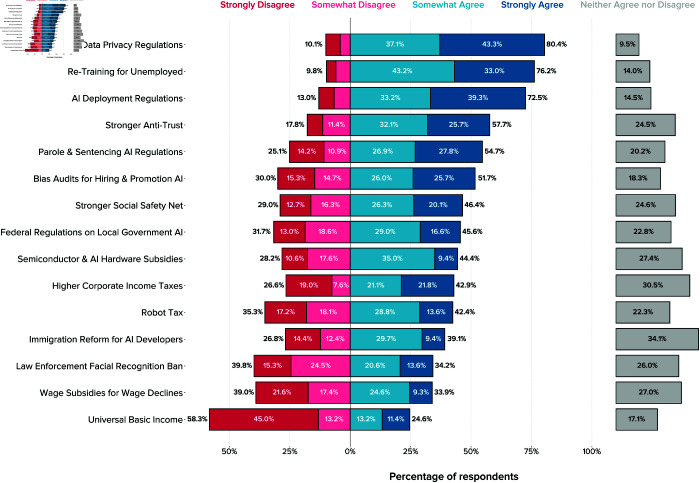
Local US officials’ views on what AI policies would be beneficial between 2025 and 2050. The figure shows unweighted relative frequencies across both survey waves.

Further, local policymakers express support for many specific AI policies addressing particular use cases and issues. Among the policies listed in Fig 3, a majority of respondents express support for stricter data privacy regulations (80%), re-training programs for those at risk of automation-driven unemployment (76%), regulation that ensures deployed AI systems are safe, robust, and fair (72%), stronger antitrust policies (58%), stricter requirements for AI use in judicial decisions (55%), and bias audits for AI used in employment decisions (52%). UBI stands out as the only AI policy proposal facing strong opposition (58% disagree). Two policies face plurality disagreement: wage subsidies for wage declines and a ban on law enforcement usage of facial recognition technologies.

Uncertainty is again evident in this category of questions, with 20% or more of respondents expressing neutral positions on 10 of 16 questions (respondents did not have the option to select IDK for QS3–4 questions). As with the questions on AI impacts, respondents express most uncertainty with respect to foreign policy and economic questions—i.e., immigration reform for AI developers (34%), higher corporate income tax (31%), semiconductor subsidies (27%), and wage subsidies for wage declines (27%). Note, however, that uncertainty is generally substantially lower here compared to questions on future AI impacts.

### Policymakers’ AI preparedness and decision-making

A final category of questions relates to how likely local policymakers think they will make decisions related to AI regulation in the coming years, as well as how informed or prepared they feel to make such decisions.

**Fig 4:**
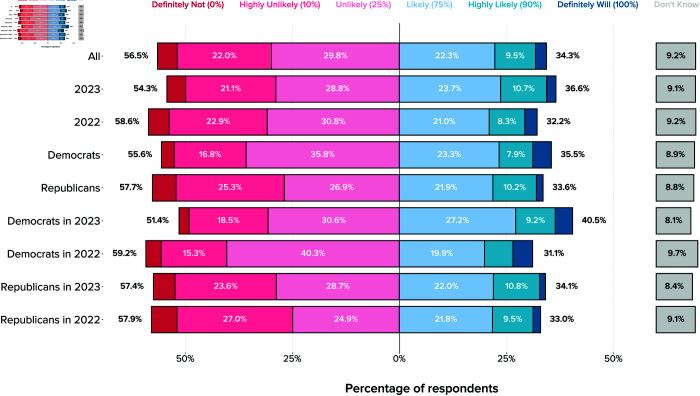
Local US officials’ responses to the question “How likely is it that your local government will have to make decisions about AI-related policies and questions in the next few years?”, with overall support and segments by party and year. The figure shows unweighted relative frequencies across both survey waves.

**Fig 5:**
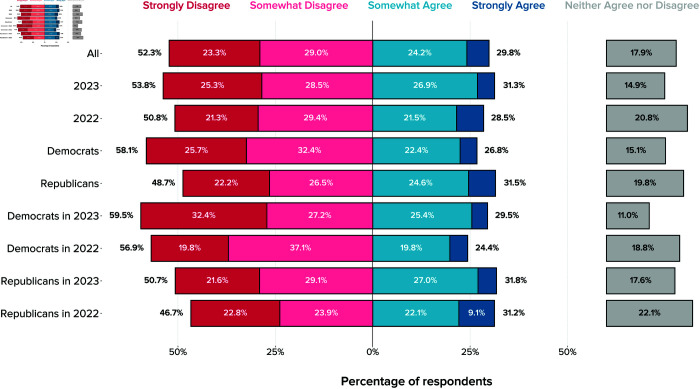
Local US officials’ agreement with the statement “Currently, if I had to make decisions about AI in my position, I would feel adequately informed to do so,” with overall support and segments by party and year. The figure shows unweighted relative frequencies across both survey waves.

Overall, local policymakers responded that they are unlikely to make decisions about AI over the next few years (57% unlikely vs. 34% likely) (Fig 4). The majority (54%) of respondents also indicated that they did not feel adequately informed to make decisions about AI at the time of the survey (Fig 5).

### Differences by party, education, and gender

We estimate the linear regression model specified in Materials and methods to assess differences in attitudes across respondent subgroups and over the 2022–2023 time period spanned by the two survey waves.

Table 4 shows that expectations of future AI impacts vary primarily by education level and gender, with smaller effects linked to age and political party affiliation. Local policymakers with higher education levels are more likely to anticipate improvements in transportation and infrastructure (β=0.121, *p* < 0.001), express greater concern about surveillance (β=0.095, *p* < 0.001), and less concern about AI’s impact on the probability of great power war (β=−0.071, *p* = 0.010). Gender differences reveal that compared to their female counterparts, male policymakers are more optimistic about AI’s impact on mental health (β=0.313, *p* = 0.003), impact on quality of life (β=0.263, *p* = 0.025) and impact on the US economy (β=0.246, *p* = 0.044). Age also has an influence, with older policymakers showing slightly lower concern about surveillance (β=−0.010, *p* = 0.001), but the difference is very close to zero.

**Table 4:** Significant Covariates from Regression Analysis for QS1–3.^f^

	Covariate						
**Question^a^**	*Age*	*Education*	*Gender* ^b^	*Race* ^c^	*Party* ^d^	*Year* ^e^	Party*Year
**QS1: Local Effects of AI**							
Surveillance Level	**–0.010** ^**^	**0.095** ^***^					
	**(–0.016, –0.005)**	**(0.051, 0.138)**					
**QS2: Local Effects of AI**							
Quality of Life			**0.263** ^*^		**–0.161** ^*^		
			**(0.094, 0.432)**		**(–0.263, –0.058)**		
Mental Health			**0.313** ^**^				
			**(0.149, 0.478)**				
Physical Health					**–0.181** ^**^		
					**(–0.278, –0.085)**		
Transportation & Infrastructure		**0.121** ^***^					
		**(0.075, 0.167)**					
**QS3: Broad Effects of AI**							
US Economy			**0.246** ^*^				
			**(0.077, 0.414)**				
Probability of Great Power War		**–0.071** ^*^					
		**(–0.113, –0.030)**					

* = *p* < 0.05, ** = *p* < 0.01, *** = *p* < 0.001. All statistically significant results in bold.

^a^ For each question, higher values represent belief that outcomes would increase.

^b^ 0 = woman and 1 = man.

^c^ 0 = white, 1 = non-white.

^d^ 0 = Democrat, 1 = independent or other party, and 2 = Republican.

^e^ 0 = 2022 and 1 = 2023.

^f^ The table includes only statistically significant covariates for the survey-weighted linear regression model as specified in Materials and methods, across both survey waves; results are hidden where adjusted p-values are above 0.05. P-values are Benjamini-Hochberg adjusted. 95% confidence intervals are shown in parentheses. IDK responses are re-coded as neutral (0) for QS1–2; respondents did not have the IDK option for QS3. Full results are in S5 Full Regression Results.

Political affiliation also shapes expectations, as Republicans are less likely than Democrats to predict positive effects of AI on physical health (β=−0.181, *p* = 0.004) and quality of life (β=−0.161, *p* = 0.023), though these differences remain modest.

We found no statistically significant differences on any questions across race, year, or the interaction between year and party. These results imply that expectations of future risks and benefits from AI are similar across white and non-white populations. Furthermore, these expectations do not appear to have shifted following the launch of ChatGPT and the accompanying increase in public awareness of AI.

Turning to attitudes towards AI governance, Table 5 shows that political party affiliation strongly correlates with AI policy preferences. Republicans express significantly less support (and Democrats express significantly more support) for a number of policies addressing specific AI use cases and issues, including retraining for AI-driven unemployment (β=−0.281, *p* = 0.002), wage subsidies for wage declines (β=0.642, *p* < 0.001), regulation that ensures deployed AI systems are safe, robust, and fair (β=−0.307, *p* < 0.001), parole and sentencing AI regulations (β=−0.397, *p* < 0.001), bias audits for hiring (β=−0.386, *p* < 0.001), immigration reform for AI developers (β=−0.317, *p* = 0.003), and taxes on company use of robots (β=−0.298, *p* = 0.011). Further, Republican local policymakers express less support for government AI regulation generally (β=−0.363, *p* < 0.001).

**Table 5:** Significant Covariates from Regression Analysis for Q4.1–QS4.^f^

	Covariate						
Question^a^	*Age*	*Education*	*Gender* ^b^	*Race* ^c^	*Party* ^d^	*Year* ^e^	Party*Year
**QS4.1: General Support for AI Regulation**							
Support AI Regulation					**-0.363** ^***^	**0.374** ^**^	
					**(–0.473, –0.252)**	**(0.178, 0.569)**	
**QS4: Policy Support**							
Stronger Anti–Trust					**–0.359** ^***^		
					**(–0.490, –0.228)**		
Robot Tax			**–0.552** ^*^		**–0.298** ^*^		
			**(–0.875, –0.229)**		**(–0.472, –0.123)**		
Higher Corporate					**–0.659** ^***^		
Income Taxes					**(–0.821, –0.497)**		
Stronger Social Safety Net					**–0.555** ^***^		
					**(–0.699, –0.412)**		
Universal Basic Income					**–0.859** ^***^		
					**(–1.015, –0.703)**		
Immigration Reform for					**–0.317** ^**^		
for AI Developers					**(–0.482, –0.151)**		
Wage Subsidies for					**–0.642** ^***^		
for Wage Declines					**(–0.790, –0.494)**		
Re–Training for Unemployed					**–0.281** ^**^		
					**(–0.422, –0.139)**		
Stricter Data Privacy		**0.116** ^**^					
Regulations		**(0.049, 0.184)**					
AI Deployment Regulations					**–0.307** ^***^		
					**(–0.446, –0.168)**		
Federal Regulations on					**–0.320** ^**^		
Local Government AI					**(–0.491, –0.149)**		
Semiconductor & AI					**–0.260** ^*^		
Hardware Subsidies					**(–0.419, –0.101)**		
Bias Audits for Hiring &					**–0.386** ^***^		
Promotion AI					**(–0.561, –0.211)**		
Parole & Sentencing					**–0.397** ^***^		
AI Regulations					**(–0.576, –0.219)**		

* = *p* < 0.05, ** = *p* < 0.01, *** = *p* < 0.001. All statistically significant results in bold.

^a^ For each question, higher values represent belief that outcomes would increase.

^b^ 0 = woman and 1 = man.

^c^ 0 = white, 1 = non-white.

^d^ 0 = Democrat, 1 = independent or other party, and 2 = Republican.

^e^ 0 = 2022 and 1 = 2023.

^f^ The table includes only statistically significant covariates for the survey-weighted linear regression model as specified in Materials and methods, across both survey waves; results are hidden where adjusted p-values are above 0.05. P-values are Benjamini-Hochberg adjusted. 95% confidence intervals are shown in parentheses. Respondents did not have the IDK option for Q4.1–QS4. Full results are in S5 Full Regression Results.

In addition to differences by political party, there are a few differences across demographic subgroups. Higher education levels correlate with stronger support for privacy regulations (β=0.117, *p* = 0.009), and men are less supportive of a robot tax compared to women (β=−0.552, *p* = 0.011).

Table 6 shows regression results using the constructed indices. These confirm some general patterns we observed in Tables 4 and 5. Republicans score significantly lower on the index aggregating personal well-being and community health questions (β=−0.174, *p* < 0.001), and Republicans’ average response across all questions is also significantly lower than Democrats (*β* = –0.400, *p* < 0.001). Higher education levels correlate with a higher score on the progress and innovation index (β=0.091, *p* < 0.001). Male policymakers are more optimistic about AI’s impacts on personal well-being and community health (β=0.266, *p* = 0.004).

**Table 6:** Significant Covariates from Regression Analysis for Constructed Indices.^f^

	Covariate							
**Question** ^a^	*Age*	*Education*	*Gender* ^b^	*Race* ^c^	*Party* ^d^	*Year* ^e^	Party*Year	*Policy*_*RegAI*_
**Constructed Indices**								
Policy Agreement					**–0.400** ^***^			
					**(–0.469, –0.330)**			
Positive Impacts (All)			**0.208** ^**^					
			**(0.100, 0.317)**					
Personal Well–Being &			**0.266** ^**^		**–0.174** ^***^			
Community Health Impacts			**(0.122, 0.411)**		**(–0.256, –0.092)**			
Progress &		**0.091** ^***^						
Innovation Impacts		**(0.052, 0.130)**						
Positive Impacts			**0.204** ^*^					
(Local Effects)			**(0.083, 0.324)**					
Positive Impacts			**0.195** ^*^					
(Broad Effects)			**(0.059, 0.332)**					

* = *p* < 0.05, ** = *p* < 0.01, *** = *p* < 0.001. All statistically significant results in bold.

^a^ For each question, higher values represent belief that outcomes would increase.

^b^ 0 = woman and 1 = man.

^c^ 0 = white, 1 = non-white.

^d^ 0 = Democrat, 1 = independent or other party, and 2 = Republican.

^e^ 0 = 2022 and 1 = 2023.

^f^ The table includes only statistically significant covariates for the survey-weighted linear regression model as specified in Materials and methods, across both survey waves; results are hidden where adjusted p-values are above 0.05. P-values are Benjamini-Hochberg adjusted. 95% confidence intervals are shown in parentheses. IDK responses are re-coded as neutral (0). Full results are in S5 Full Regression Results. Index definitions are in S4 Indices Definitions.

In addition, the indices relating to positive AI future impacts reveal a new pattern. These two indices were constructed by aggregating all survey questions relating to positive AI impacts, i.e., potential societal benefits rather than societal risks. These indices include societal benefits such as increases in jobs, quality of life, democracy, and innovation but exclude risks such as increasing political polarization, surveillance, and international conflicts. Examining the positive impacts indices, we see that male policymakers are more likely to expect greater benefits from AI on the local community level (β=0.204, *p* = 0.011), more broadly in the country (β=0.195, *p* = 0.048), and overall across all societal benefits (β=0.208, *p* = 0.003).

### Differences from 2022 to 2023

Generally, both expectations of AI impacts and attitudes towards specific AI policies remained stable over 2022–2023. We found no statistically significant differences across the two years or the interaction between year and party in any of the questions relating to AI impacts or to specific AI policies such as stronger anti-trust laws, a robot tax, or UBI.

A notable exception, however, is support for government regulation, which increased significantly from 2022 to 2023 (β=0.374, *p* = 0.003). The magnitude of the change in support over 2022-2023, alongside the difference in support by party affiliation, are the two largest group effects we observe in the study (see Table 5).

**Fig 6:**
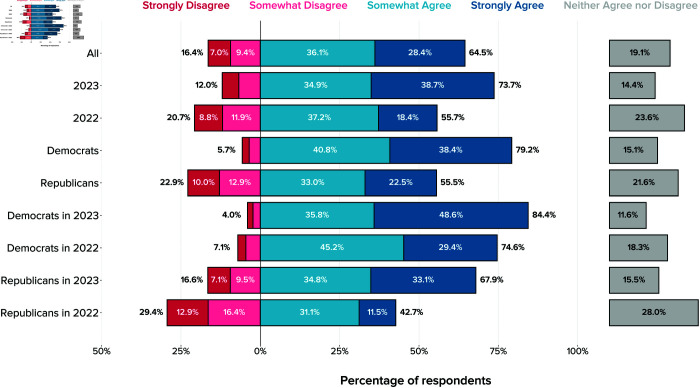
Local US officials’ agreement with the statement “AI should be regulated by the government,” with overall support and segments by party and year. The figure shows unweighted relative frequencies across both survey waves.

Although the interaction between year and party is not statistically significant, it is worth noting that the increase in support was much greater among Republicans compared to Democrats. As Fig 6 shows, support increased across the board from 56% agree or strongly agree in 2022 to 74% agree or strongly agree in 2023. Republicans, however, shifted from minority agreement in 2022 (43%) to majority agreement in 2023 (68%). The shift among Democrats, who already exhibited high levels of support in 2022, was more modest, rising from 75% agreement in 2022 to 84% in 2023.

## Discussion

The results of our study offer valuable insights into how local US policymakers view the societal impacts and governance of AI. Local officials in our study conveyed both optimism about AI’s potential and significant concern about its risks, consistent with prior surveys of the US public and AI experts. Local officials are optimistic about AI’s impacts on innovation, infrastructure, and transportation. But local officials also share concerns about specific risks also highlighted in previous research, including increasing inequality, job loss, and data security threats [8,34,64–67]).

A noteworthy feature of this study was its timing, spanning a period of heightened attention to AI governance, including the release of ChatGPT and subsequent federal and state-level regulatory activity in 2023. Bipartisan support for AI regulation increased markedly between the 2022 and 2023 surveys, mirroring broader national developments, such as President Biden’s Executive Order on AI governance and the surge in AI-related legislation at both the state and federal levels during 2023. The convergence in support for government regulation, with both Republicans and Democrats in favor in 2023, suggests a growing recognition across party lines of the need for oversight in the face of rapid advancements in AI. However, we also see marked partisan differences in support for specific AI policies, with Democrat support generally being significantly higher.

Despite concerns over risks and growing support for regulation, we also found high levels of uncertainty, reported lack of preparedness among policymakers, and limited expectations of near-term involvement in policymaking. These results are particularly striking given the surge in AI-related policy initiatives during this period. They suggest that increased attention to AI at the national and state levels has not yet translated into greater confidence or preparedness among local officials. This is also notable in light of the decentralized nature of US policymaking, which often places local officials at the forefront of implementing state and federal policies.

### Limitations

While the survey results provide important insight into local policymakers’ attitudes and expectations, which help us to understand the trajectory of AI policy-making, our study also has some limitations.

First, we asked policymakers to consider potential future impacts of AI during the 2025–2050 time period. Yet, most policymakers are not trained in forecasting; they may lack expertise and experience to make accurate or confident predictions about the trajectory of AI. It is challenging even for experts to make predictions about the costs and impacts of emerging technologies [68]. This means that we should expect substantial variation in reported expectations of impact, as well as notable rates of non-response. We believe some of this variation and non-response reflects the uncertain trajectory of AI development and is consistent with the finding that most policymakers’ feel inadequately informed about AI. Nevertheless, some of the uncertainty could be due to the difficulty of forecasting in general, as well as the difficulty of forecasting in the context of emerging technologies in particular.

Second, any survey has to contend with selection effects and other considerations related to the survey sample. For example, it may be the case that local US officials who chose to participate differ systematically from those who did not. Details on the representativeness of the sample in terms of area-level population size, 2020 Presidential election, and population educational characteristics can be found in the Supporting Information (S8 Sample Representativeness). Sample medians are slightly skewed toward larger districts, are more Democratic, and are more educated than population median; generally, however, sample medians remain relatively close to population medians, suggesting that the samples are somewhat representative. To improve sample representativeness in our regression analysis, we also used weights computed based on these area-level characteristics. Our regressions found no statistically significant differences based on the area-level characteristics, or even for most individual-level respondent characteristics. In turn, it is important to note that the results also do not necessarily generalize to policymakers in other countries or to state and federal-level policymakers.

Third, the study design and choice of questions introduce further limitations. The questions we asked and specific items chosen reflect our subjective prioritization of topics and may not fully capture the range of concerns that policymakers hold. The phrasing of items likely also has an effect on the expressed beliefs and opinions of individuals in regard to the impacts they expect from AI and the specific policies they support. There may also be effects of positive and negative frames of impacts on expressed beliefs. Additionally, it is important to note that the design was not a longitudinal panel but completed in separate survey waves with different samples. Therefore, we cannot assess individual-level changes, and there may be cohort effects across the waves. Relatedly, our post-ChatGPT sample was collected only six months after the public release of ChatGPT; respondents may not have fully updated their views during this time period, so it is possible that our results do not fully reflect or may underestimate attitudinal changes.

Fourth, an important consideration is the high frequency of “I don’t know” responses to many questions. Likert-scale responses are treated as continuous variables in the linear regressions, which approach does not adequately address the unique nature of IDK responses. In our main analysis, we re-coded IDK responses as neutral (0) to ensure these responses were included in the models. However, this method has its limitations, as it assumes that uncertainty or lack of knowledge aligns with neutrality, which may not accurately reflect respondents’ perspectives. To address this, we also conducted supplementary analyses using imputed IDK responses, which are presented in S6 Alternative Regression Results. This analysis found that all coefficients that were statistically significant at *p* < 0.05 when IDK are re-coded as neutral are also significant when IDK responses are imputed. In addition, the model fit on imputed data sees 21 more statistically significant coefficients.

Fifth, our sample contained high levels of missing data, in part due to our planned missingness design. Rates of missingness were as high as 69% for some questions in QS4; MICE procedures have been shown in simulations to be robust even with much higher rates of missingness [69,70], but high rates of missingness may still bias the imputations. To improve the robustness of our imputations, we impute a large number of datasets, and we also conduct robustness checks (see S5 Full Regression Results) on the imputation procedure.

Finally, our statistical analyses have inherent limitations. Given the breadth of regressions run, we adjusted significance thresholds using the Benjamini-Hochberg procedure to control for false discovery rates. While this reduces the risk of false positives, it also lowers the power of the regressions, increasing the likelihood of false negatives. Moreover, the relatively small sample size, when broken into subgroups or conditions, limits the precision of some estimates.

### Future directions

The findings of our study point to several promising avenues for future research. First, there is a pressing need for further capacity-building initiatives to equip local, state, and national policymakers with the knowledge and tools necessary to navigate AI’s complexities. Research could play a critical role in identifying the specific informational needs of policymakers, including areas where they feel least prepared, such as the technical aspects of AI, its economic implications, and its potential impacts on international relations.

Longitudinal studies could be particularly valuable for tracking how policymakers’ attitudes and competencies evolve over time, especially in response to rapid advancements in AI technology and policy interventions. Such research would provide a clearer picture of how exposure to AI technologies and governance frameworks shapes perceptions and decision-making capabilities. Future studies could also broaden the scope to include other key populations, such as state-level policymakers, federal agencies, and private sector leaders.

Cross-national comparisons offer another avenue for exploration. Understanding how US local policymakers’ attitudes align or diverge from those in other countries—particularly in regions with distinct regulatory frameworks such as the EU or Asia—could provide valuable insights into how differing political, economic, and cultural contexts shape AI governance approaches and inform strategies for international coordination.

Finally, future research should delve deeper into the drivers of uncertainty among policymakers. Investigating the sources of their knowledge gaps, as well as the factors that contribute to shifts in confidence and preparedness, would help design targeted interventions to address these challenges. This could include studies on how exposure to training programs, expert advisory panels, and public debates influences policymaker readiness to engage with AI-related issues.

## Conclusion

This study highlights several important policy implications for AI governance in the US, particularly at the local level. First, there may be growing bipartisan support for AI regulation among local US officials, rising from 55% in 2022 (six months before ChatGPT release) to 74% in 2023 (six months after ChatGPT release). While Democrats consistently show greater support for many specific regulatory policies addressing AI challenges such as unemployment and AI safety and fairness, Republican support for government oversight in general has increased significantly.

Of course, in the future, AI discourse may also become more politicized and polarized as the political landscape evolves. Indeed, the persistence of polarization in specific policy preferences already raises important concerns. While Democrats and Republicans share similar views on AI risks, their divergent support for targeted regulatory measures—which align with partisan cues related to other issues such as immigration, tax policy, criminal justice reform, and social welfare—suggests that public opinion on AI-related issues could eventually become divided along traditional pro- and anti-regulation lines. This dynamic has the potential to exacerbate state-level policy fragmentation. Such disparities could create uncertainty and inefficiencies for businesses, workers, and consumers operating across jurisdictions.

Finally, substantial levels of uncertainty and reported lack of preparedness among local officials highlight a potential need for capacity-building initiatives. Policymakers require targeted training and resources to navigate the complexities of AI governance, particularly in addressing its broader economic, social, and international implications. Bridging these knowledge gaps could empower local governments to play a more active role in shaping AI policy and ensure that governance efforts reflect both local priorities and broader national and global objectives.

## Supporting information

S1 Additional figures

S1.1 Fig Democratic US officials’ expectations of the local impacts of AI between 2025 and 2050. The figure shows unweighted relative frequencies for QS1 across both survey waves.
(TIFF)

S1.2 Fig Republican US officials’ expectations of the local impacts of AI between 2025 and 2050. The figure shows unweighted relative frequencies for QS1 across both survey waves.
(TIFF)

S1.3 Fig Democratic US officials’ expectations of the local impacts of AI between 2025 and 2050. The figure shows unweighted relative frequencies for QS2 across both survey waves.
(TIFF)

S1.4 Fig Republican US officials’ expectations of the local impacts of AI between 2025 and 2050. The figure shows unweighted relative frequencies for QS2 across both survey waves.
(TIFF)

S1.5 Fig Democratic US officials’ expectations of the broad impacts of AI between 2025 and 2050. The figure shows unweighted relative frequencies for QS3 across both survey waves.
(TIFF)

S1.6 Fig Republican US officials’ expectations of the broad impacts of AI between 2025 and 2050. The figure shows unweighted relative frequencies for QS3 across both survey waves.
(TIFF)

S1.7 Fig Democratic US officials’ views on what AI policies would be beneficial between 2025 and 2050. The figure shows unweighted relative frequencies for QS4 across both survey waves.
(TIFF)

S1.8 Fig Republican US officials’ views on what AI policies would be beneficial between 2025 and 2050. The figure shows unweighted relative frequencies for QS4 across both survey waves.
(TIFF)

S1.9 Fig Local US officials’ expectations in 2022 of the local impacts of AI between 2025 and 2050. The figure shows unweighted relative frequencies for QS1 for the 2022 wave only.
(TIFF)

S1.10 Fig Local US officials’ expectations in 2023 of the local impacts of AI between 2025 and 2050. The figure shows unweighted relative frequencies for QS1 for the 2023 wave only.
(TIFF)

S1.11 Fig Local US officials’ expectations in 2022 of the local impacts of AI between 2025 and 2050. The figure shows unweighted relative frequencies for QS2 for the 2022 wave only.
(TIFF)

S1.12 Fig Local US officials’ expectations in 2023 of the local impacts of AI between 2025 and 2050. The figure shows unweighted relative frequencies for QS2 for the 2023 wave only.
(TIFF)

S1.13 Fig Local US officials’ expectations in 2022 of the local impacts of AI between 2025 and 2050. The figure shows unweighted relative frequencies for QS3 for the 2022 wave only.
(TIFF)

S1.14 Fig Local US officials’ expectations in 2023 of the local impacts of AI between 2025 and 2050. The figure shows unweighted relative frequencies for QS3 for the 2023 wave only.
(TIFF)

S1.15 Fig Local US officials’ views in 2022 on what AI policies would be beneficial between 2025 and 2050. The figure shows unweighted relative frequencies for QS4 for the 2022 wave only.
(TIFF)

S1.16 Fig Local US officials’ views in 2023 on what AI policies would be beneficial between 2025 and 2050. The figure shows unweighted relative frequencies for QS4 for the 2023 wave only.
(TIFF)

S2 Variable definitions. (PDF)

S3 Survey text. (PDF)

S4 Indices definitions. (PDF)

S5 Full regression results. (PDF)

S5.1 Fig Convergence Plots for 8 Randomly Selected MICE Imputations, Across 200 Iterations Each. (TIFF)

S5.2 Fig Density Plots for 8 Randomly Selected MICE Imputations. (TIFF)

S6 Alternative regression results. (PDF)

S7 Survey experiment. (PDF)

S7.1 Fig Local US officials’ responses to the question “Do you think that AI will have an overall positive or negative effect on the US from now until 2100?”, with overall support and segments by treatment group and year. The figure shows unweighted relative frequencies across both survey waves.
(TIFF)

S8 Sample representativeness. (PDF)
