# Supplementary material for: Local US officials’ views on the impacts and governance of AI: Evidence from 2022 and 2023 survey waves
Source: PLoS One. 2025 Oct 6;20(10):e0332919. doi: 10.1371/journal.pone.0332919 (PMC12500108; doi:10.1371/journal.pone.0332919)
Supplement: S1 [file pone.0332919.s001.pdf]

S1 Additional figures Figures S1.1–S1.16 contain relative frequencies for QS1–4, segmented by political party and by year. Note that all figures display unweighted statistics across both survey waves. Labels for bars containing relative frequencies of less than 7.0% are hidden for readability.

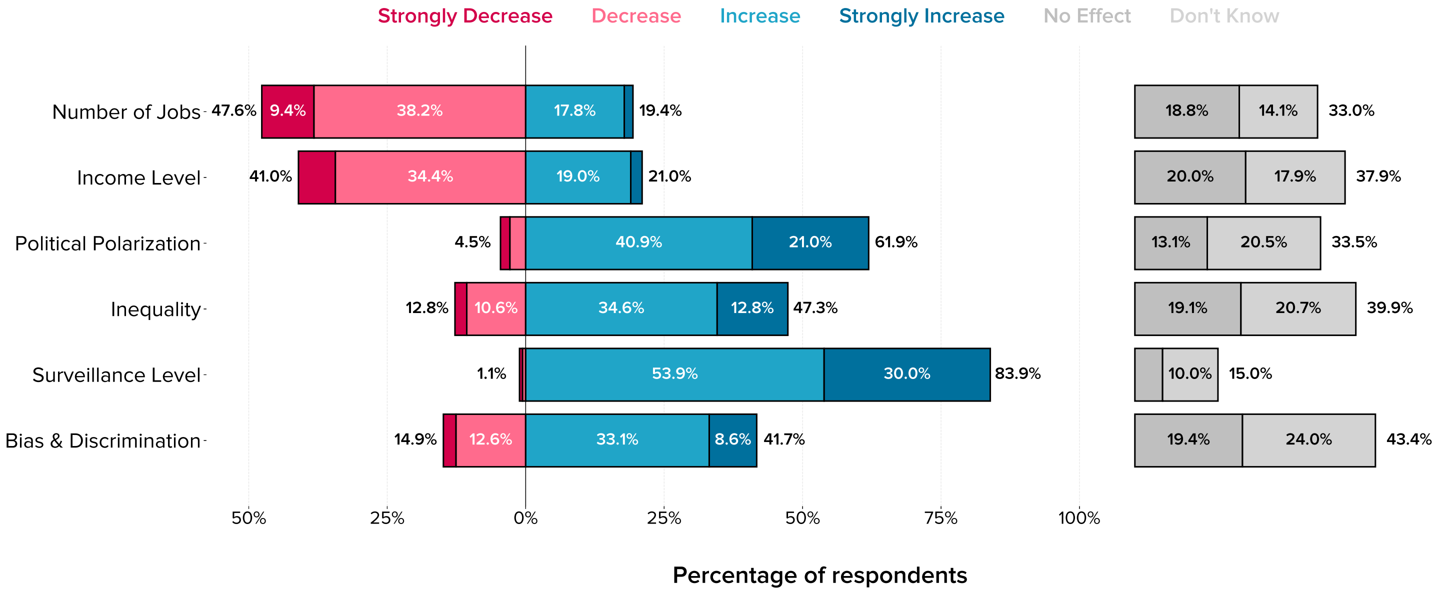

Fig S1.1. Democratic US officials' expectations of the local impacts of AI between 2025 and 2050. The figure shows unweighted relative frequencies for QS1 across both survey waves.

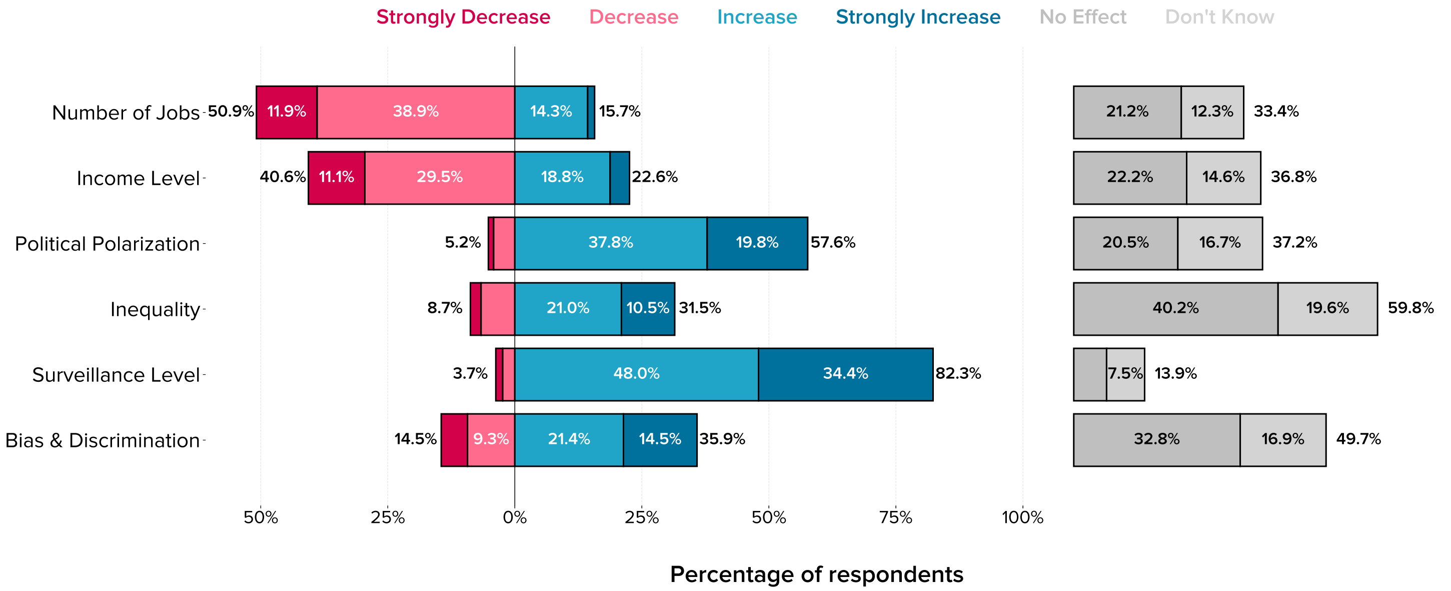

Fig S1.2. Republican US officials' expectations of the local impacts of AI between 2025 and 2050. The figure shows unweighted relative frequencies for QS1 across both survey waves.

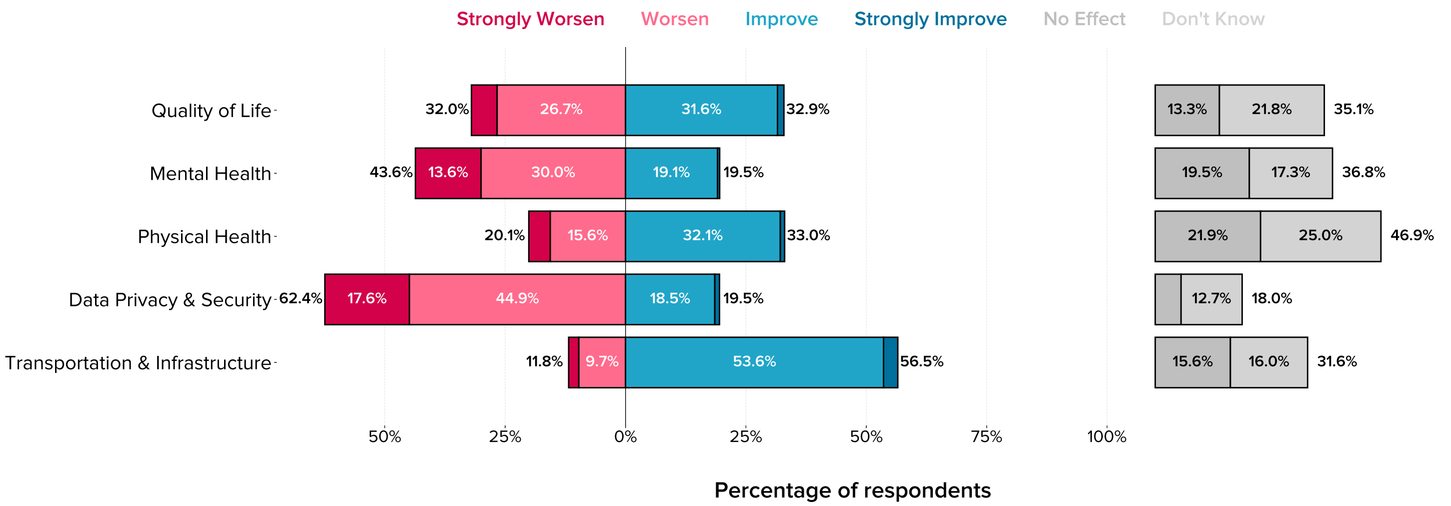

Fig S1.3. Democratic US officials' expectations of the local impacts of AI between 2025 and 2050. The figure shows unweighted relative frequencies for QS2 across both survey waves.

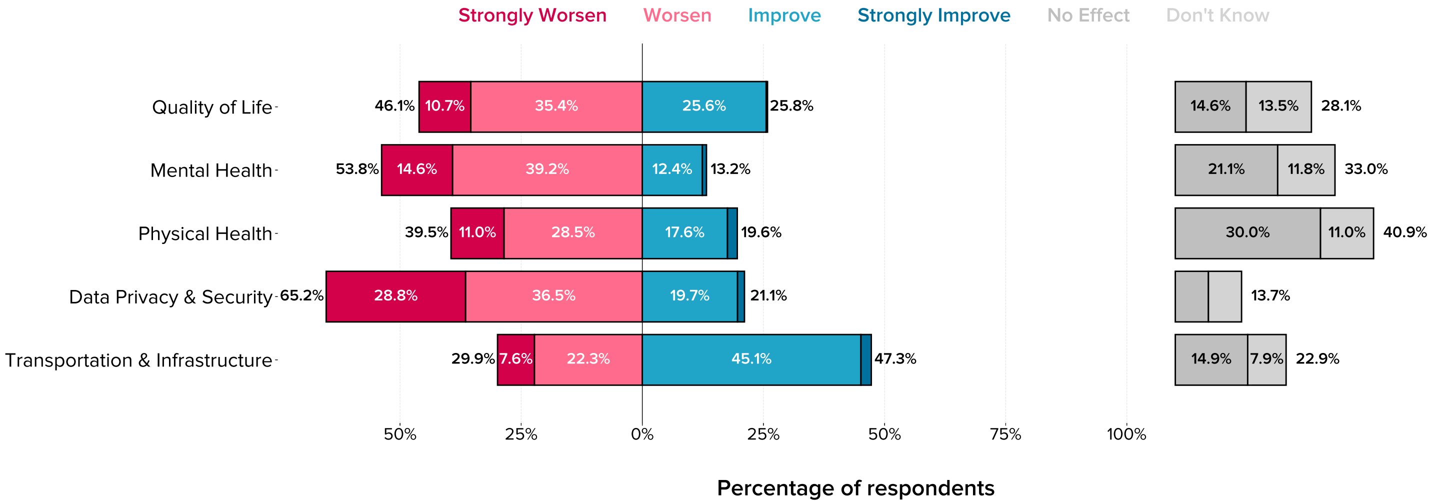

Fig S1.4. Republican US officials' expectations of the local impacts of AI between 2025 and 2050. The figure shows unweighted relative frequencies for QS2 across both survey waves.

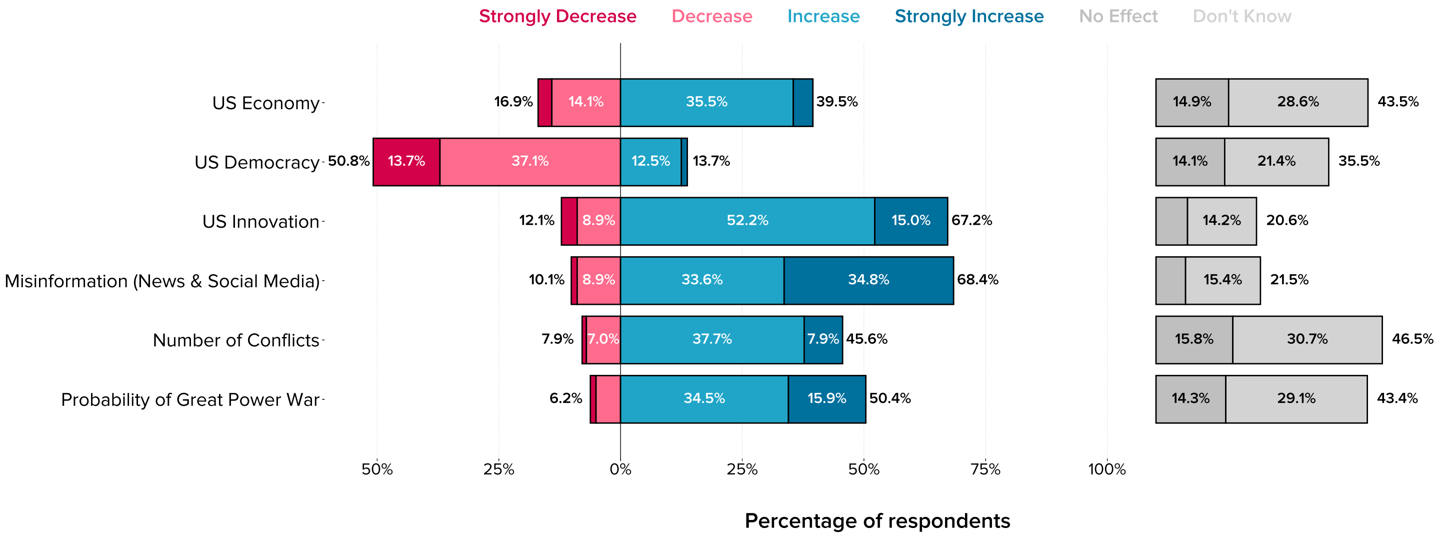

Fig S1.5. Democratic US officials' expectations of the broad impacts of AI between 2025 and 2050. The figure shows unweighted relative frequencies for QS3 across both survey waves.

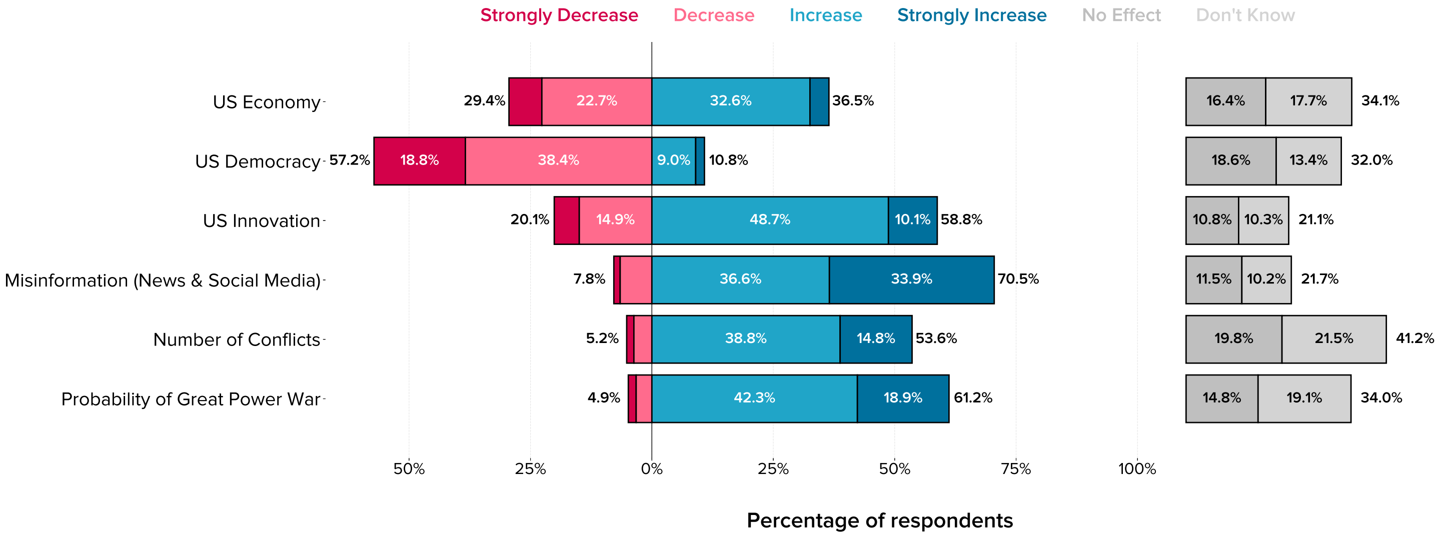

Fig S1.6. Republican US officials' expectations of the broad impacts of AI between 2025 and 2050. The figure shows unweighted relative frequencies for QS3 across both survey waves.

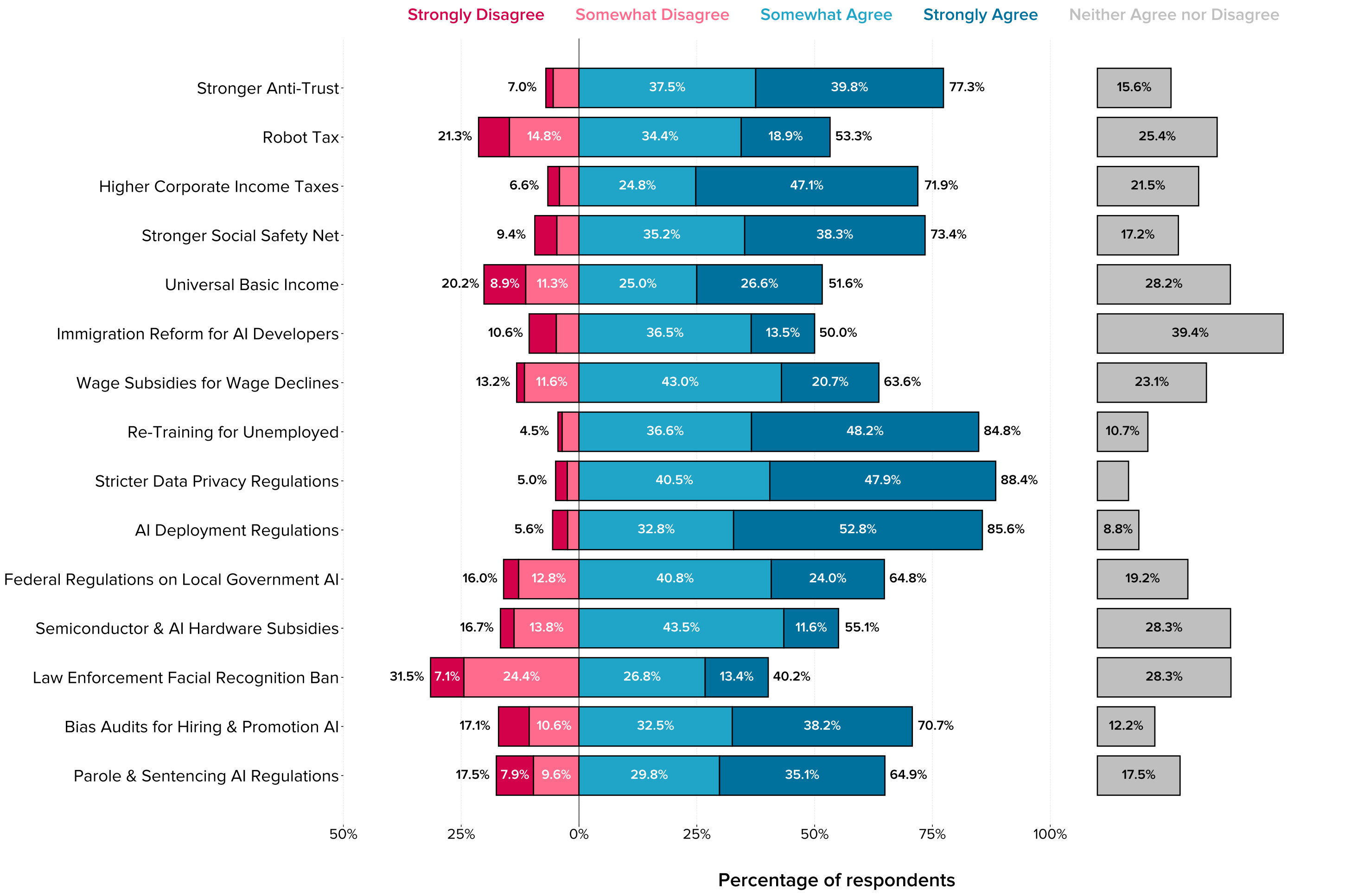

Fig S1.7. Democratic US officials' views on what AI policies would be beneficial between 2025 and 2050. The figure shows unweighted relative frequencies for QS4 across both survey waves.

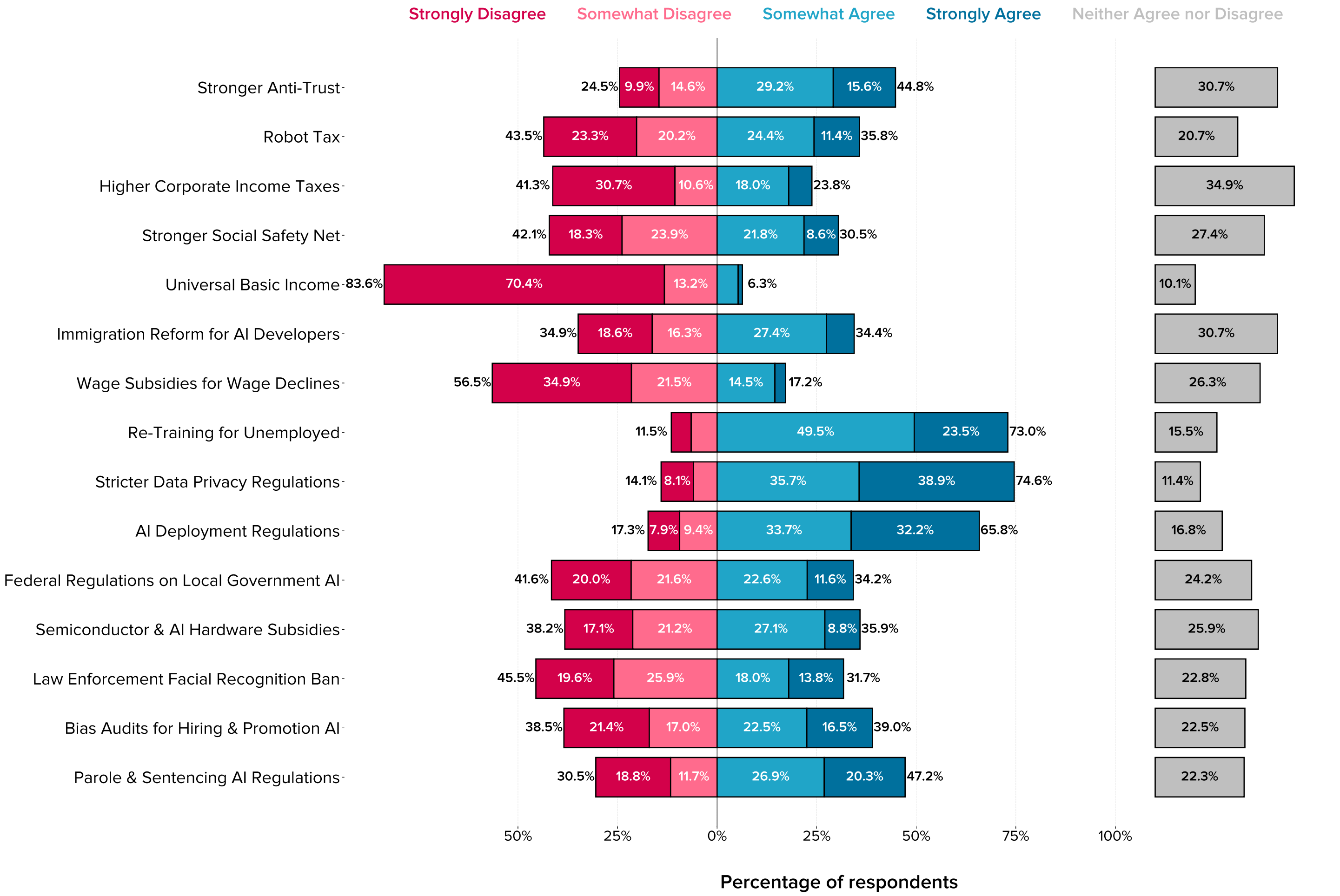

Fig S1.8. Republican US officials' views on what AI policies would be beneficial between 2025 and 2050. The figure shows unweighted relative frequencies for QS4 across both survey waves.

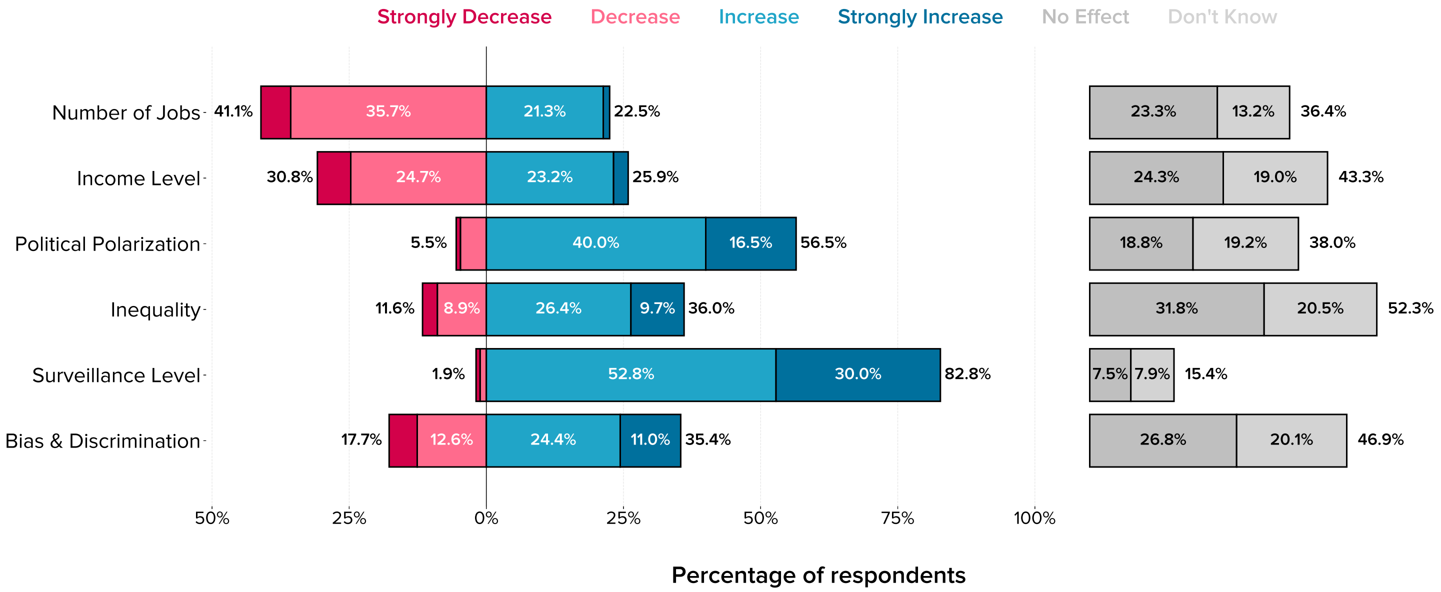

Fig S1.9. Local US officials' expectations in 2022 of the local impacts of AI between 2025 and 2050. The figure shows unweighted relative frequencies for QS1 for the 2022 wave only.

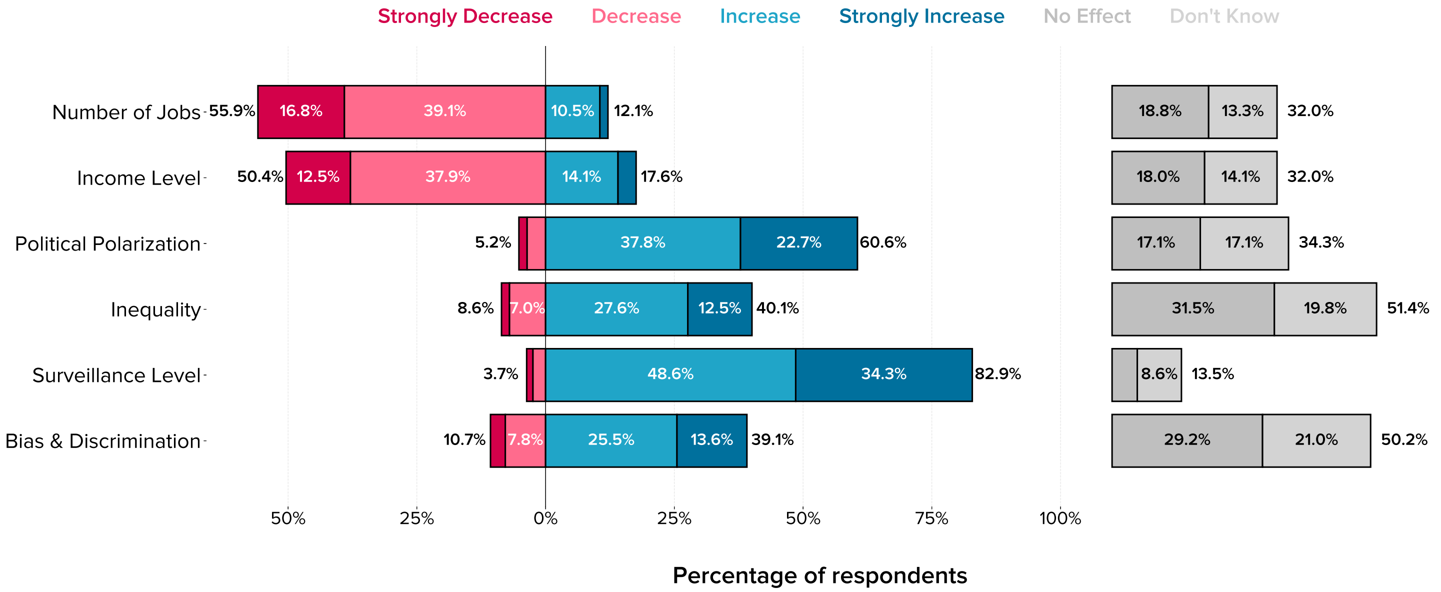

Fig S1.10. Local US officials' expectations in 2023 of the local impacts of AI between 2025 and 2050. The figure shows unweighted relative frequencies for QS1 for the 2023 wave only.

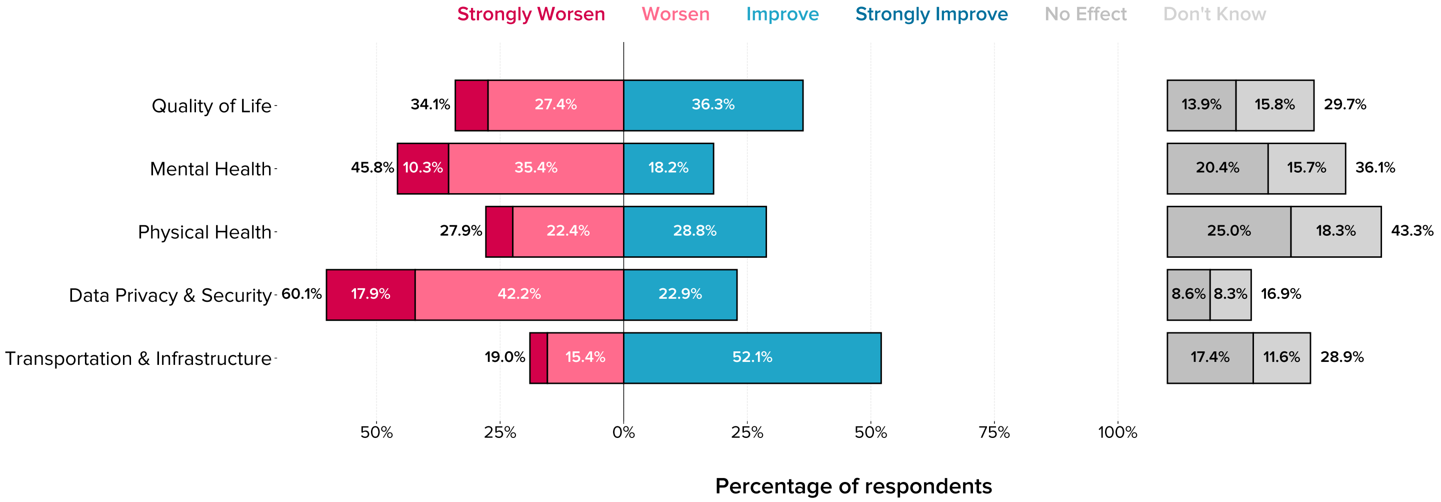

Fig S1.11. Local US officials' expectations in 2022 of the local impacts of AI between 2025 and 2050. The figure shows unweighted relative frequencies for QS2 for the 2022 wave only.

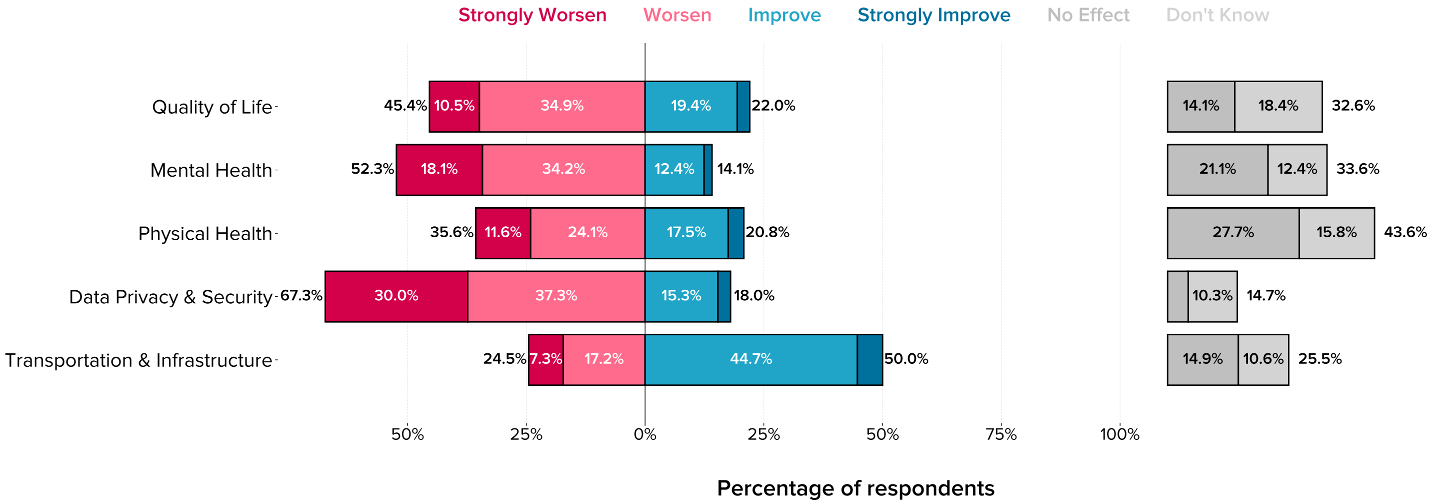

Fig S1.12. Local US officials' expectations in 2023 of the local impacts of AI between 2025 and 2050. The figure shows unweighted relative frequencies for QS2 for the 2023 wave only.

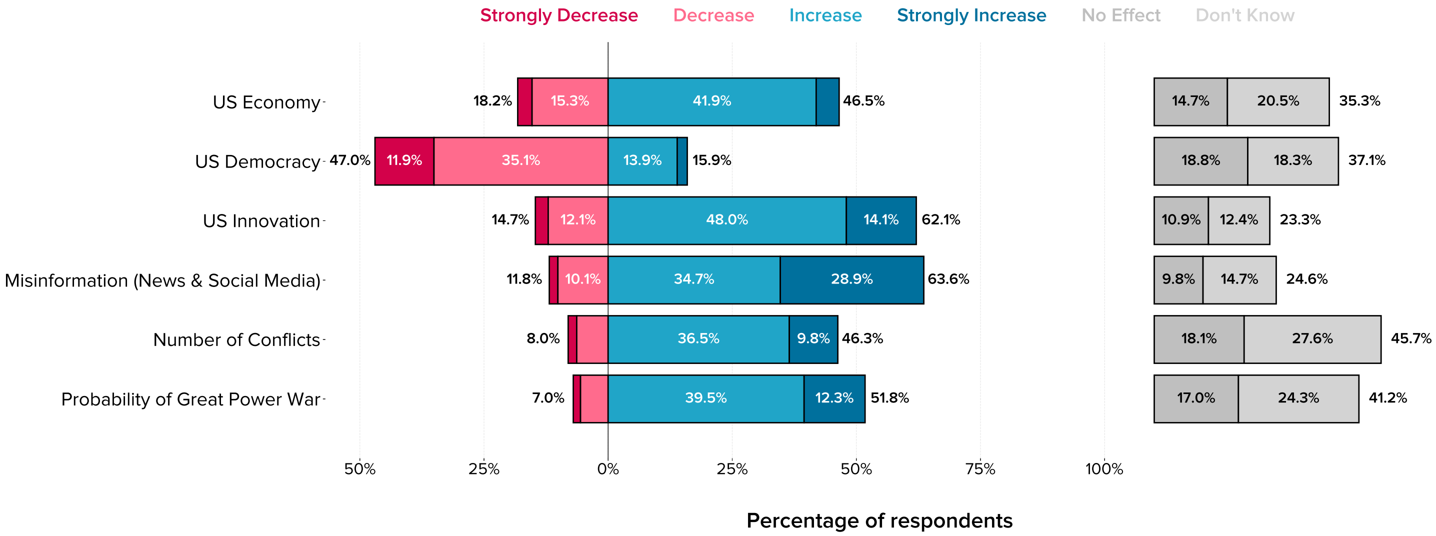

Fig S1.13. Local US officials' expectations in 2022 of the local impacts of AI between 2025 and 2050. The figure shows unweighted relative frequencies for QS3 for the 2022 wave only.

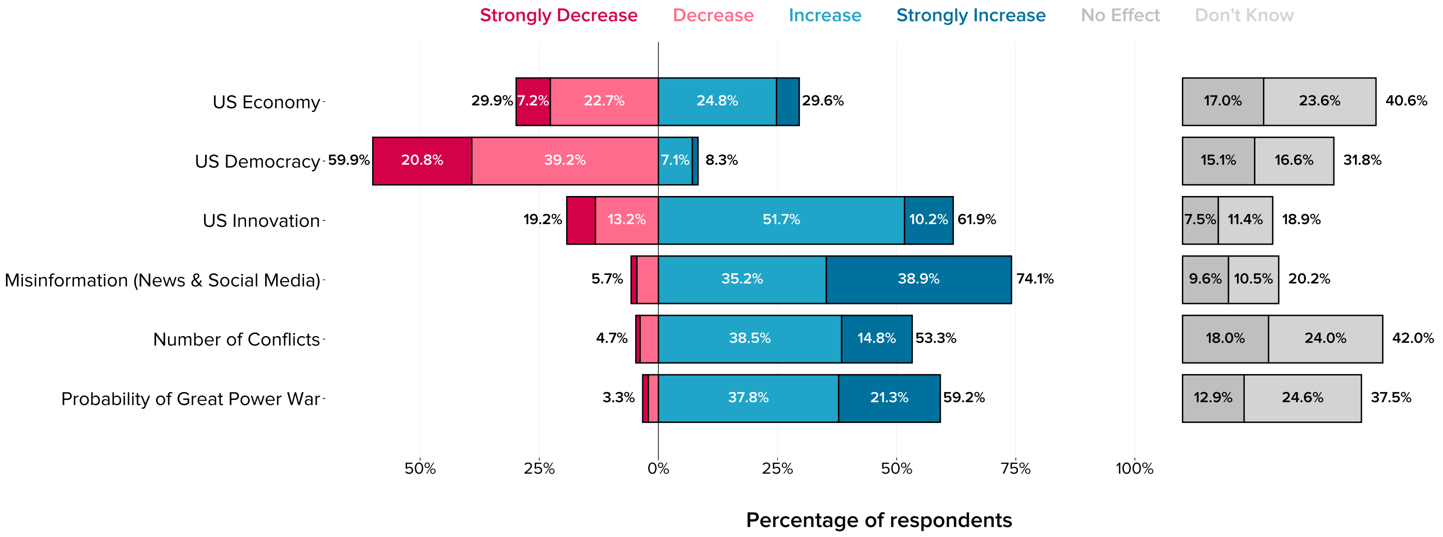

Fig S1.14. Local US officials' expectations in 2023 of the local impacts of AI between 2025 and 2050. The figure shows unweighted relative frequencies for QS3 for the 2023 wave only.

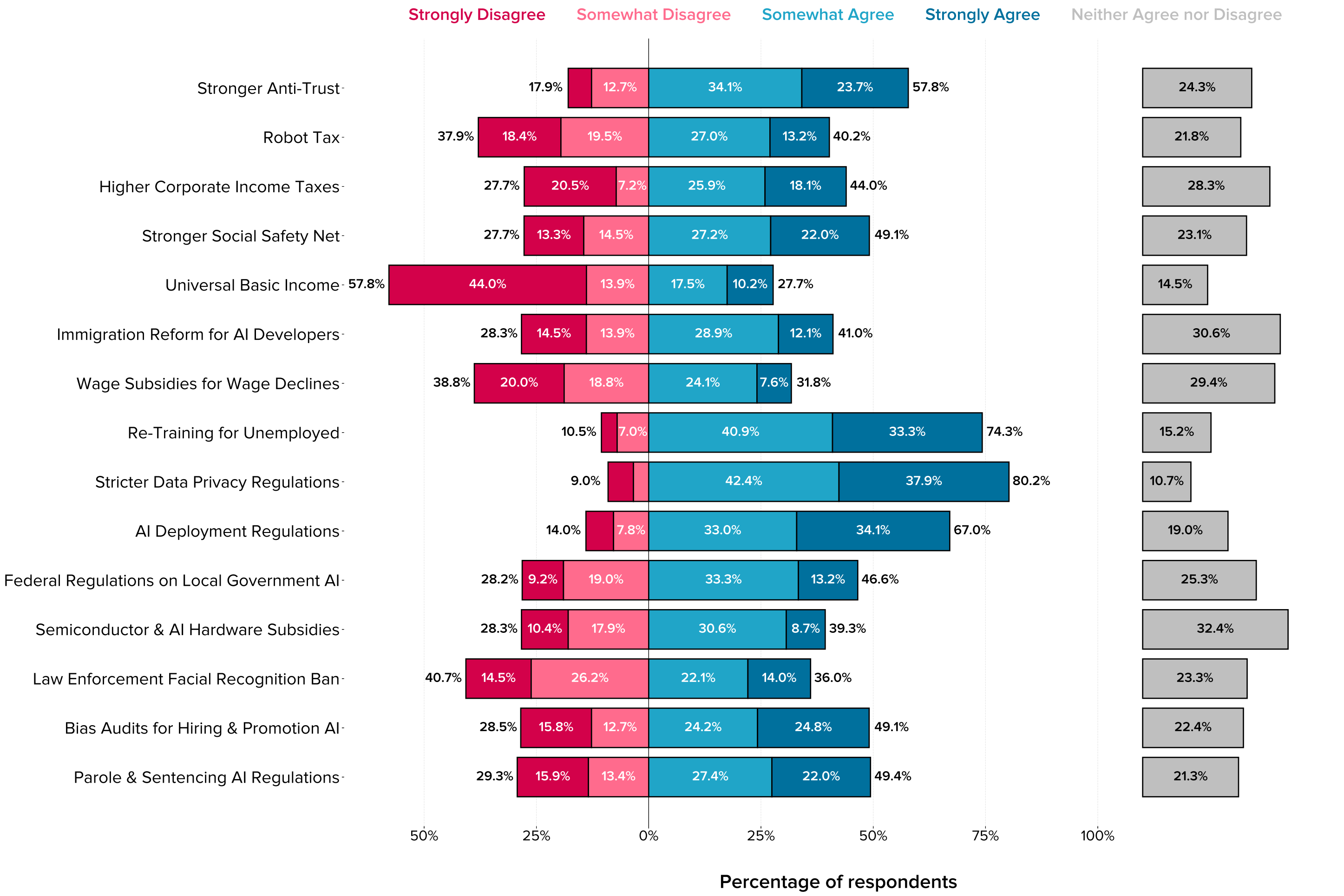

Fig S1.15. Local US officials' views in 2022 on what AI policies would be beneficial between 2025 and 2050. The figure shows unweighted relative frequencies for QS4 for the 2022 wave only.

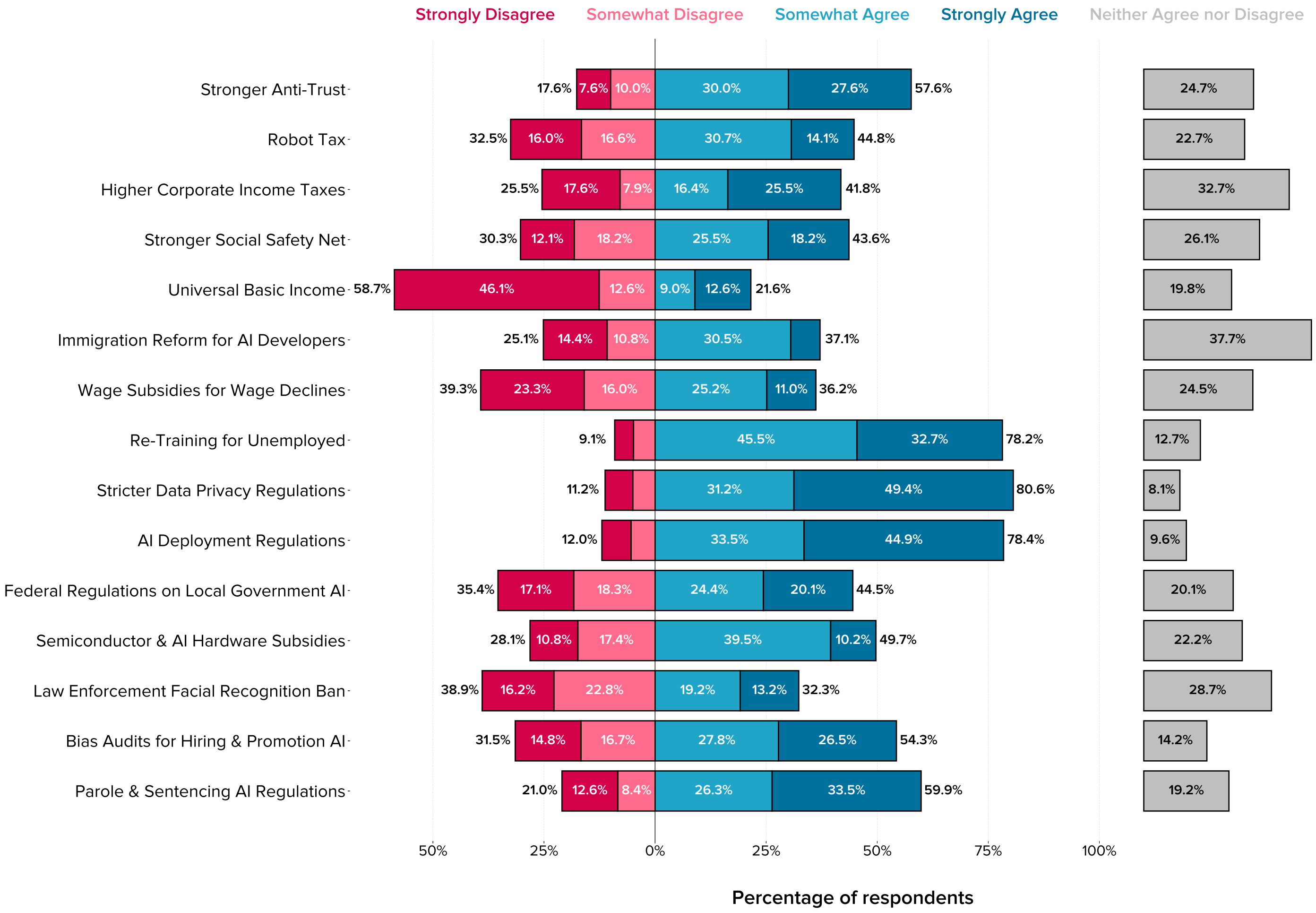

Fig S1.16. Local US officials' views in 2023 on what AI policies would be beneficial between 2025 and 2050. The figure shows unweighted relative frequencies for QS4 for the 2023 wave only.
