# Supplementary figures and images for: Local US officials’ views on the impacts and governance of AI: Evidence from 2022 and 2023 survey waves
Source: PLoS One. 2025 Oct 6;20(10):e0332919. doi: 10.1371/journal.pone.0332919 (PMC12500108; doi:10.1371/journal.pone.0332919)

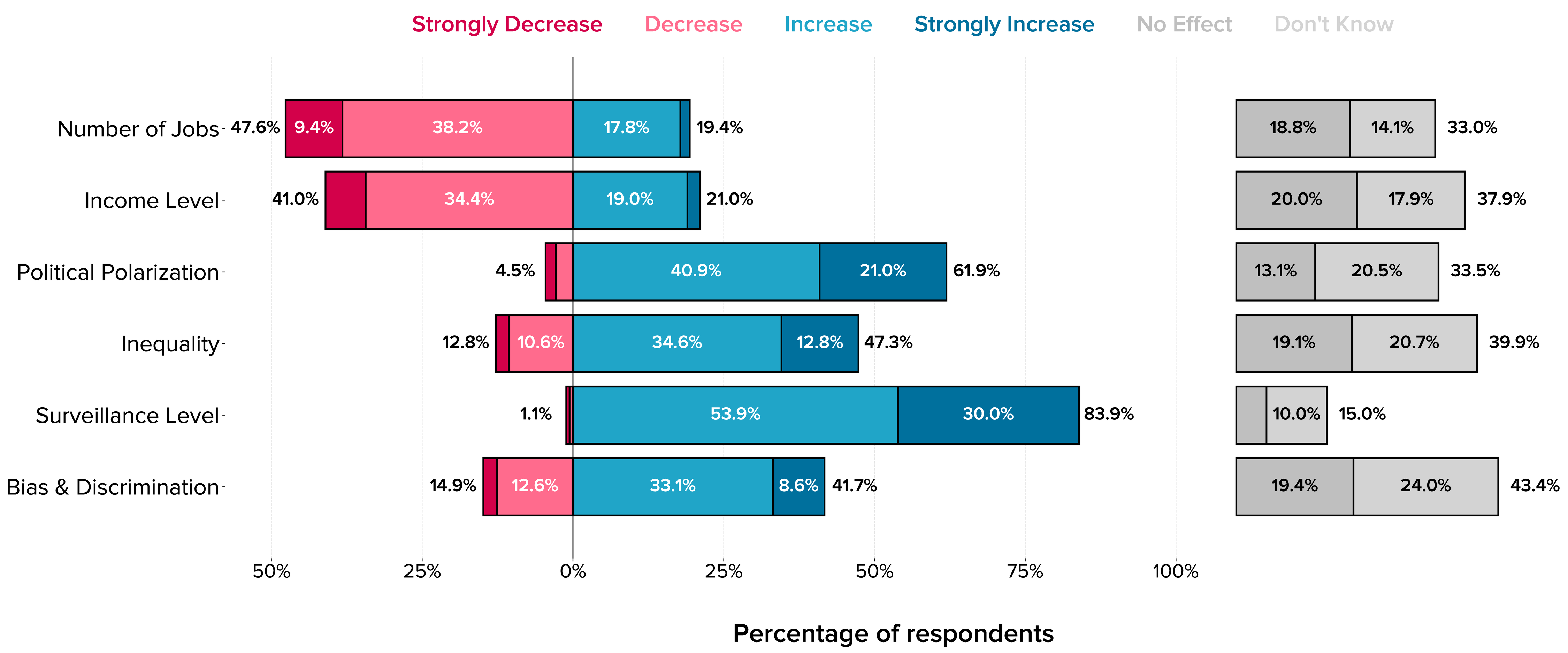

Supplement: S1.1 Fig — The figure shows unweighted relative frequencies for QS1 across both survey waves. (TIFF) [file pone.0332919.s002.tif]

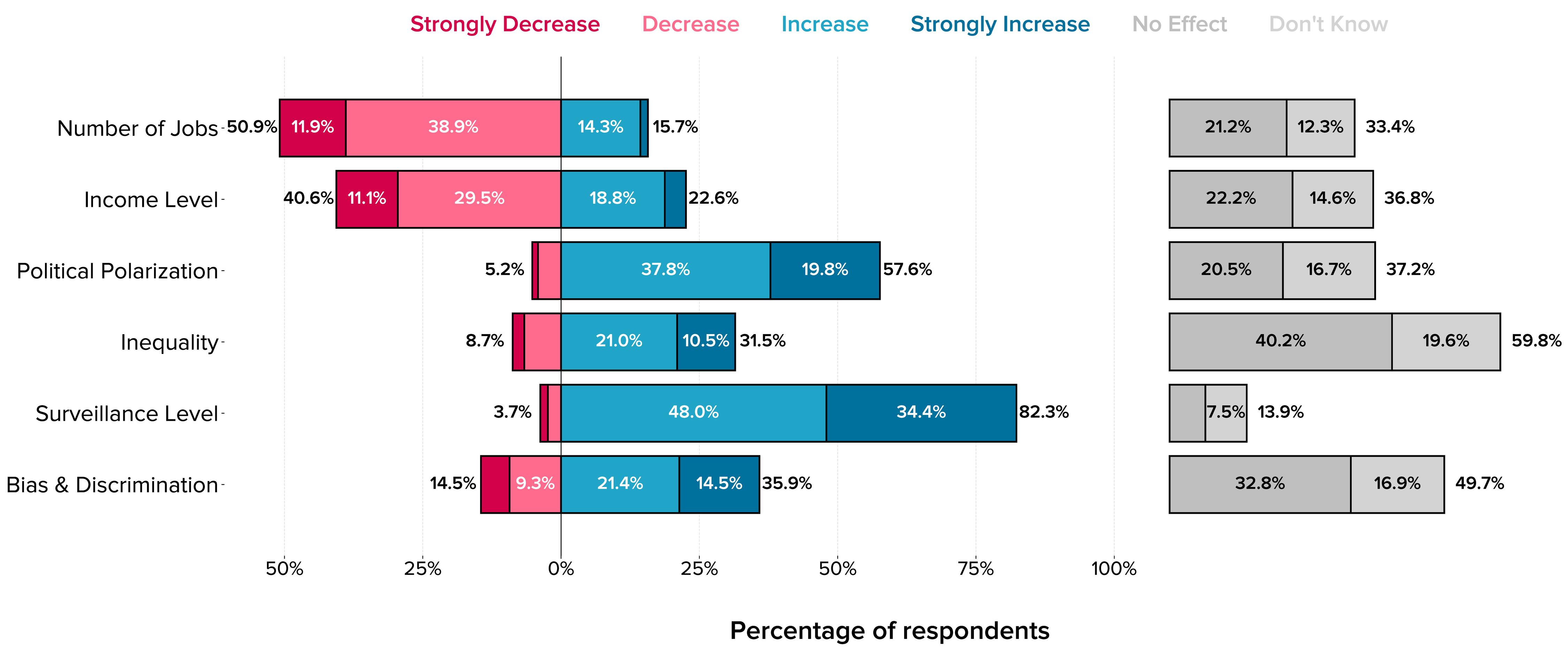

Supplement: S1.2 Fig — The figure shows unweighted relative frequencies for QS1 across both survey waves. (TIFF) [file pone.0332919.s003.tif]

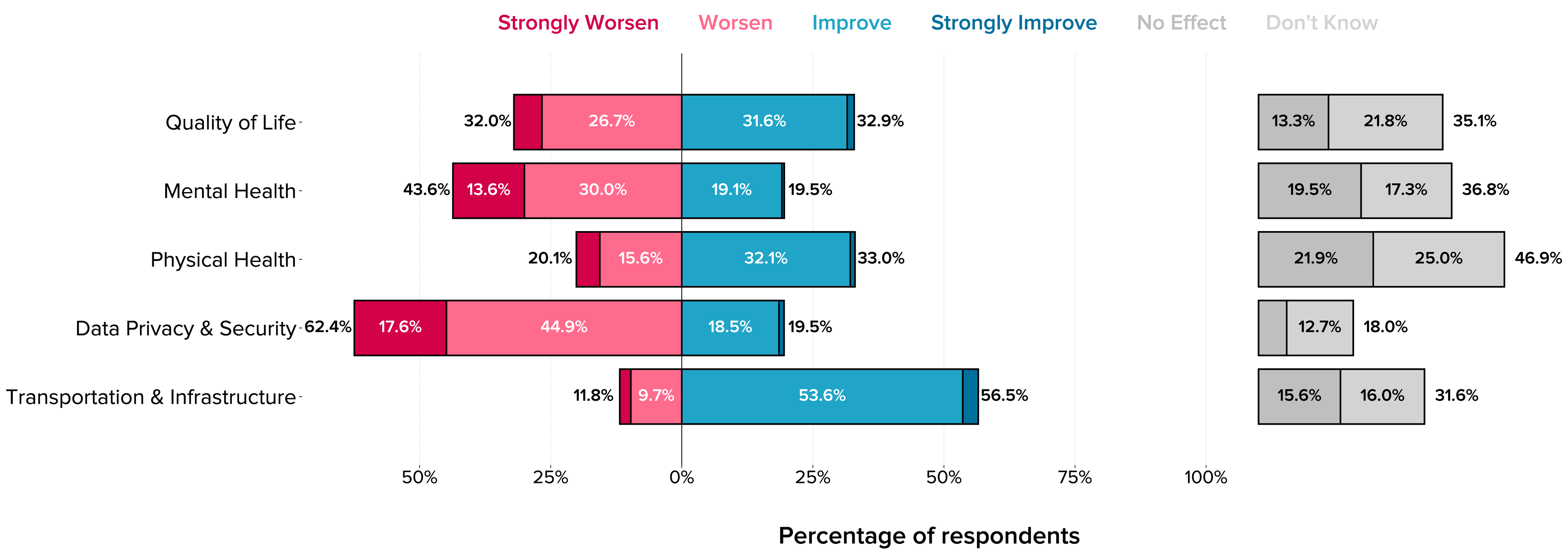

Supplement: S1.3 Fig — The figure shows unweighted relative frequencies for QS2 across both survey waves. (TIFF) [file pone.0332919.s004.tif]

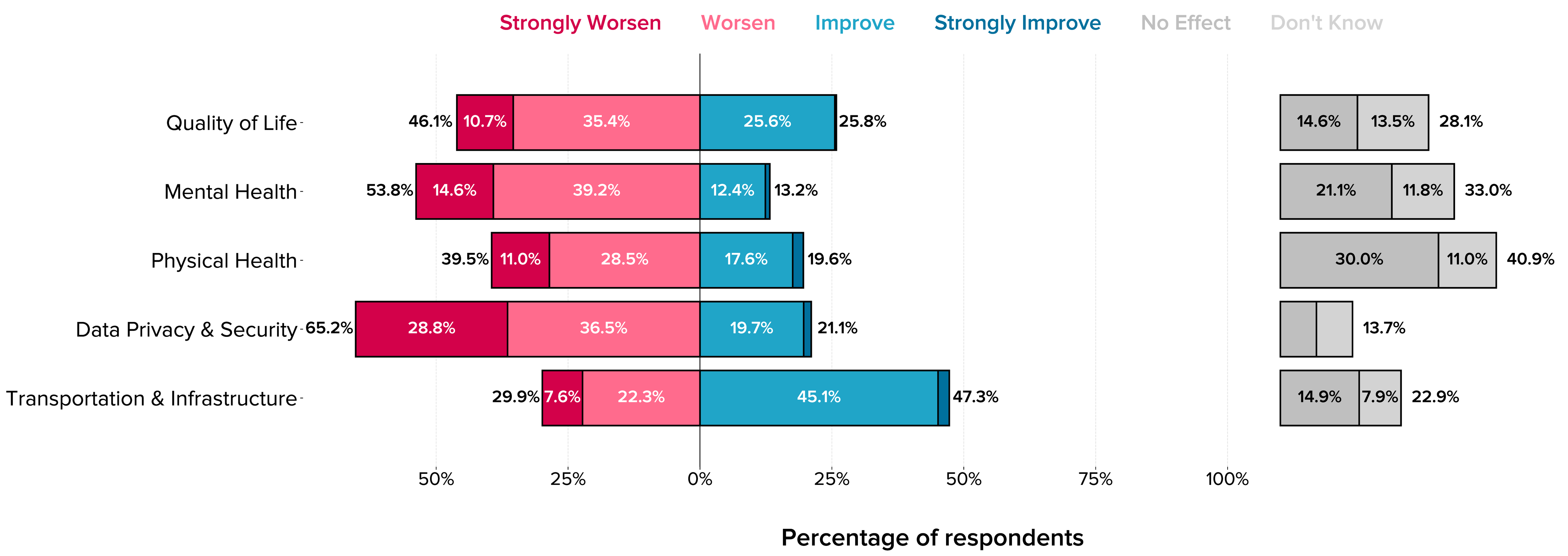

Supplement: S1.4 Fig — The figure shows unweighted relative frequencies for QS2 across both survey waves. (TIFF) [file pone.0332919.s005.tif]

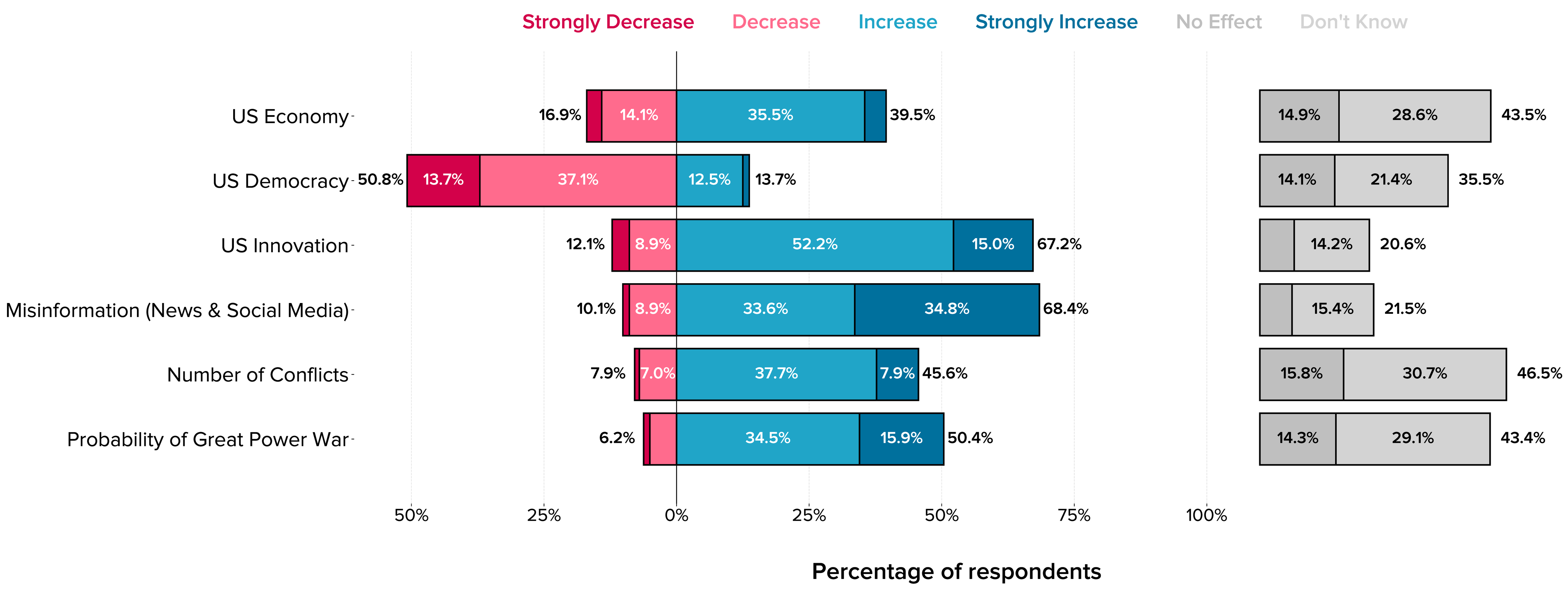

Supplement: S1.5 Fig — The figure shows unweighted relative frequencies for QS3 across both survey waves. (TIFF) [file pone.0332919.s006.tif]

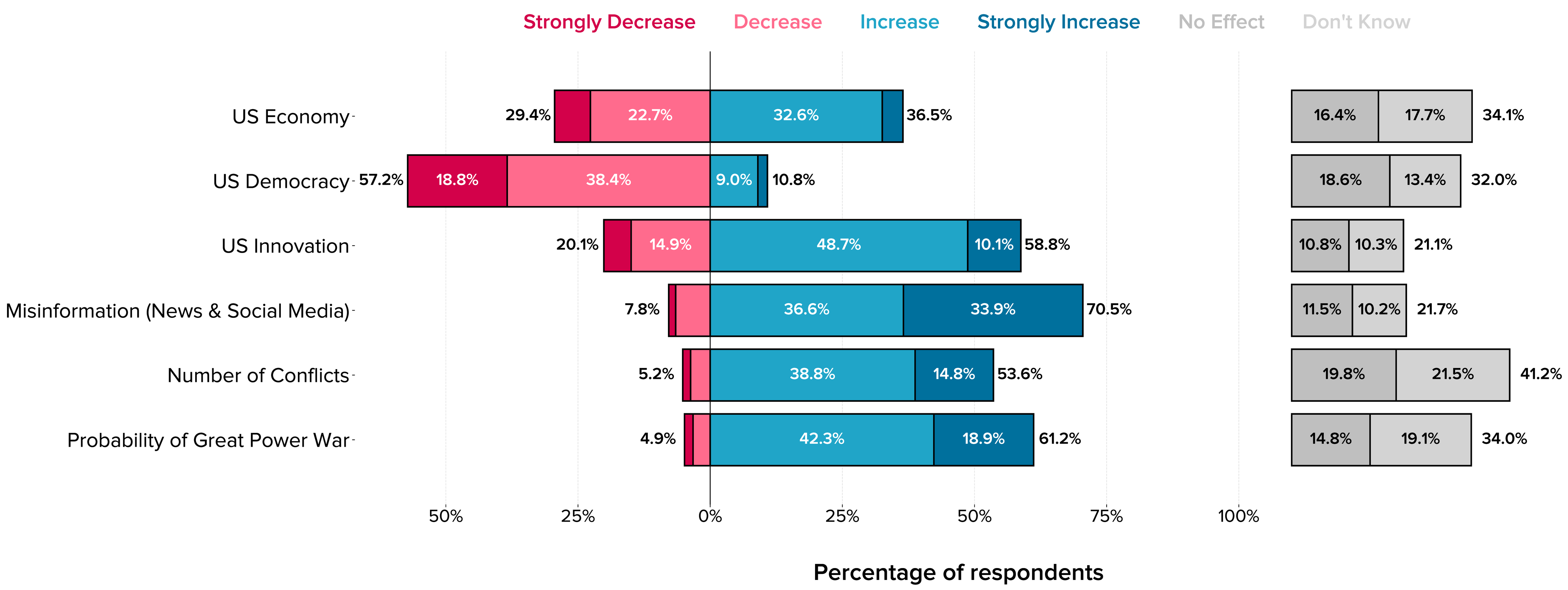

Supplement: S1.6 Fig — The figure shows unweighted relative frequencies for QS3 across both survey waves. (TIFF) [file pone.0332919.s007.tif]

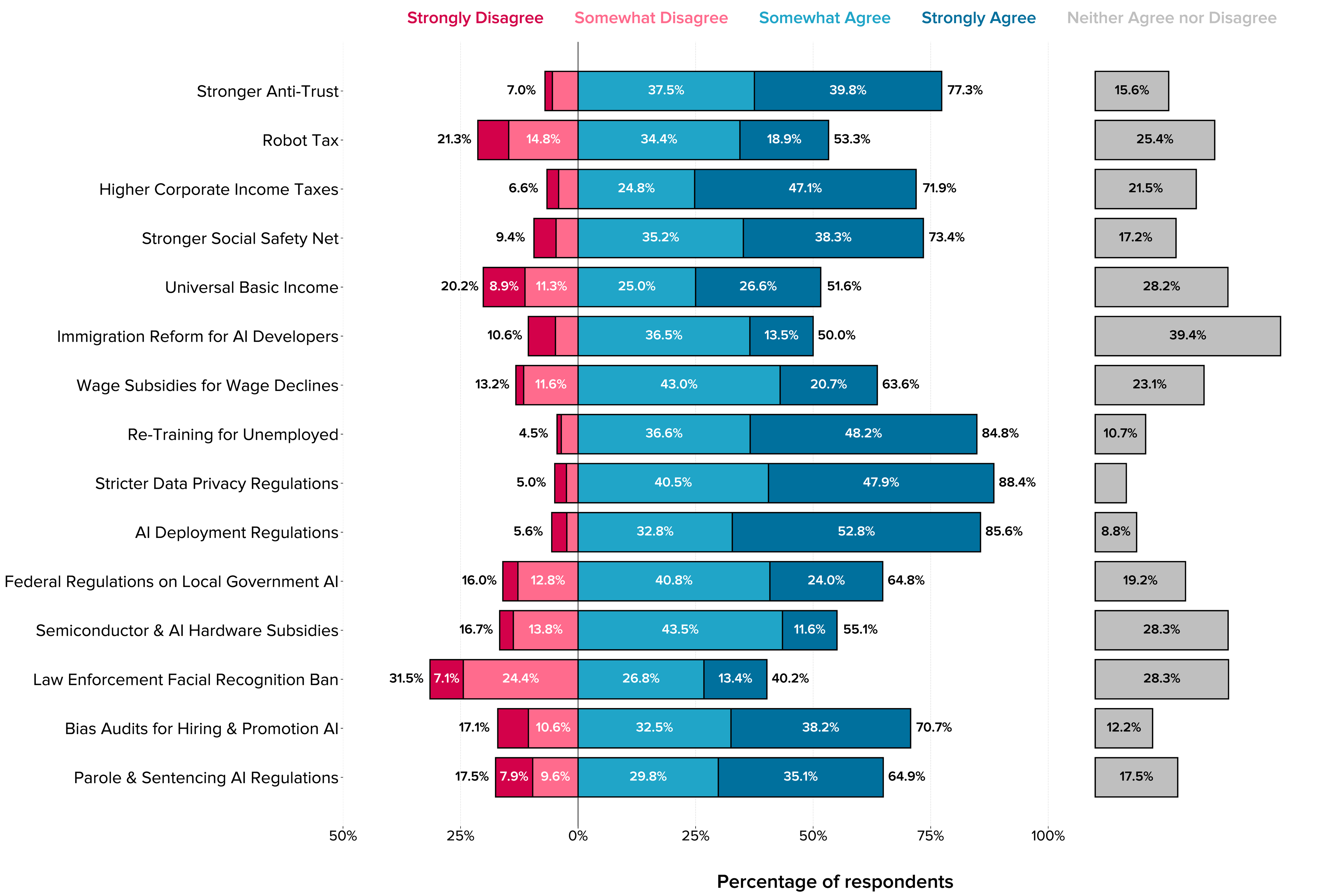

Supplement: S1.7 Fig — The figure shows unweighted relative frequencies for QS4 across both survey waves. (TIFF) [file pone.0332919.s008.tif]

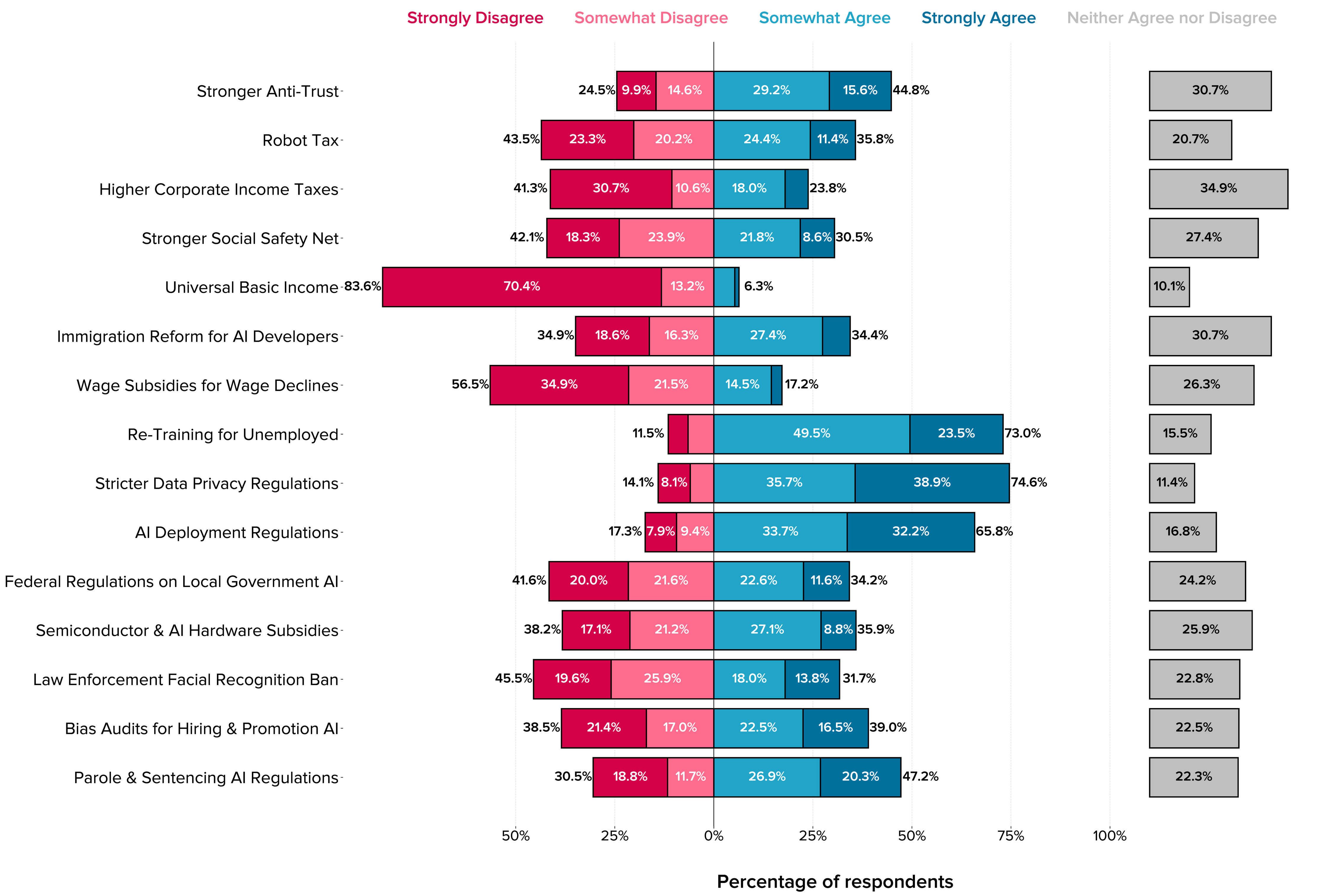

Supplement: S1.8 Fig — The figure shows unweighted relative frequencies for QS4 across both survey waves. (TIFF) [file pone.0332919.s009.tif]

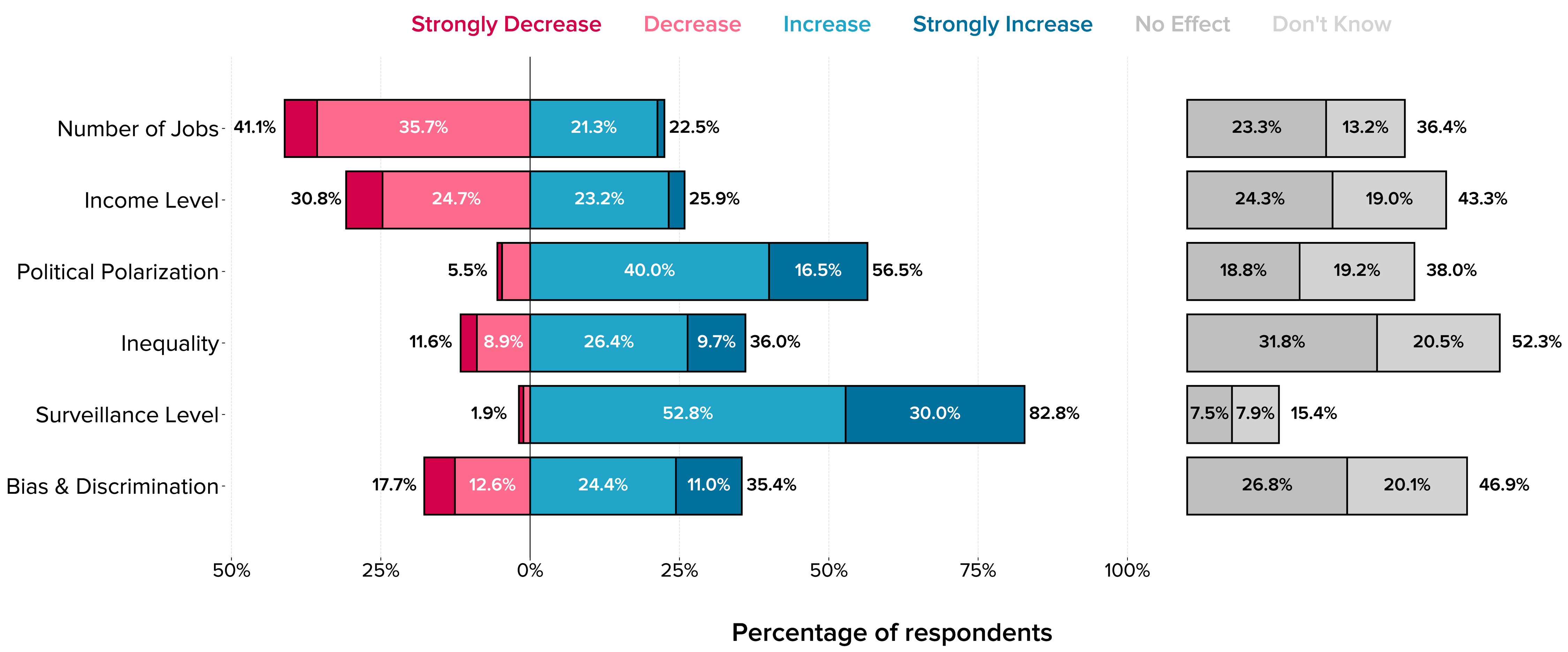

Supplement: S1.9 Fig — The figure shows unweighted relative frequencies for QS1 for the 2022 wave only. (TIFF) [file pone.0332919.s010.tif]

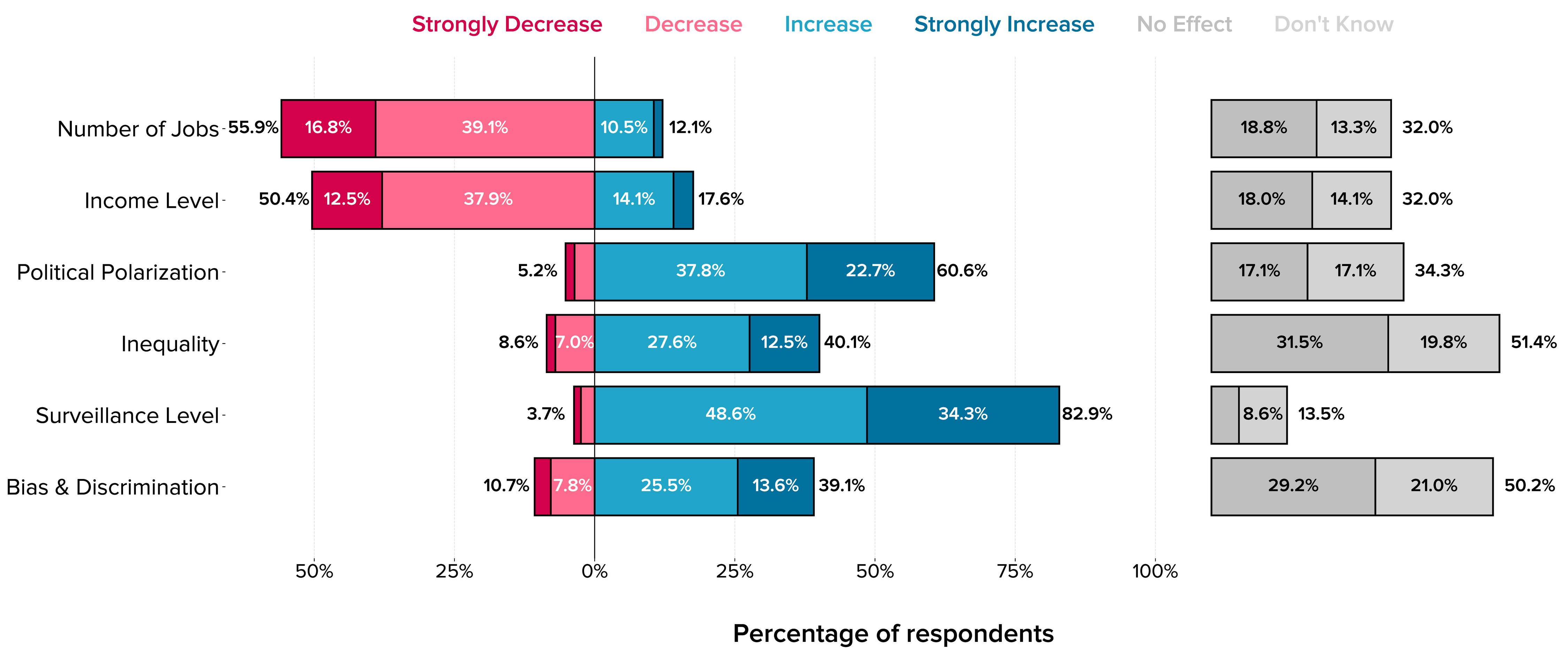

Supplement: S1.10 Fig — The figure shows unweighted relative frequencies for QS1 for the 2023 wave only. (TIFF) [file pone.0332919.s011.tif]

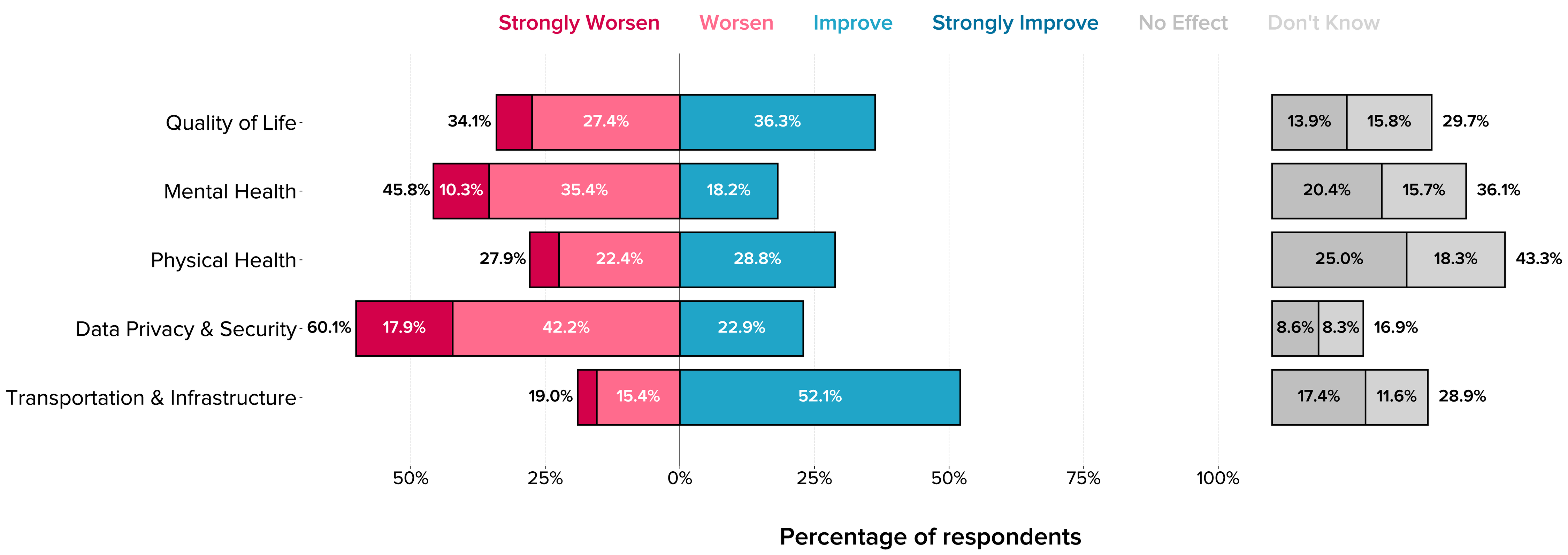

Supplement: S1.11 Fig — The figure shows unweighted relative frequencies for QS2 for the 2022 wave only. (TIFF) [file pone.0332919.s012.tif]

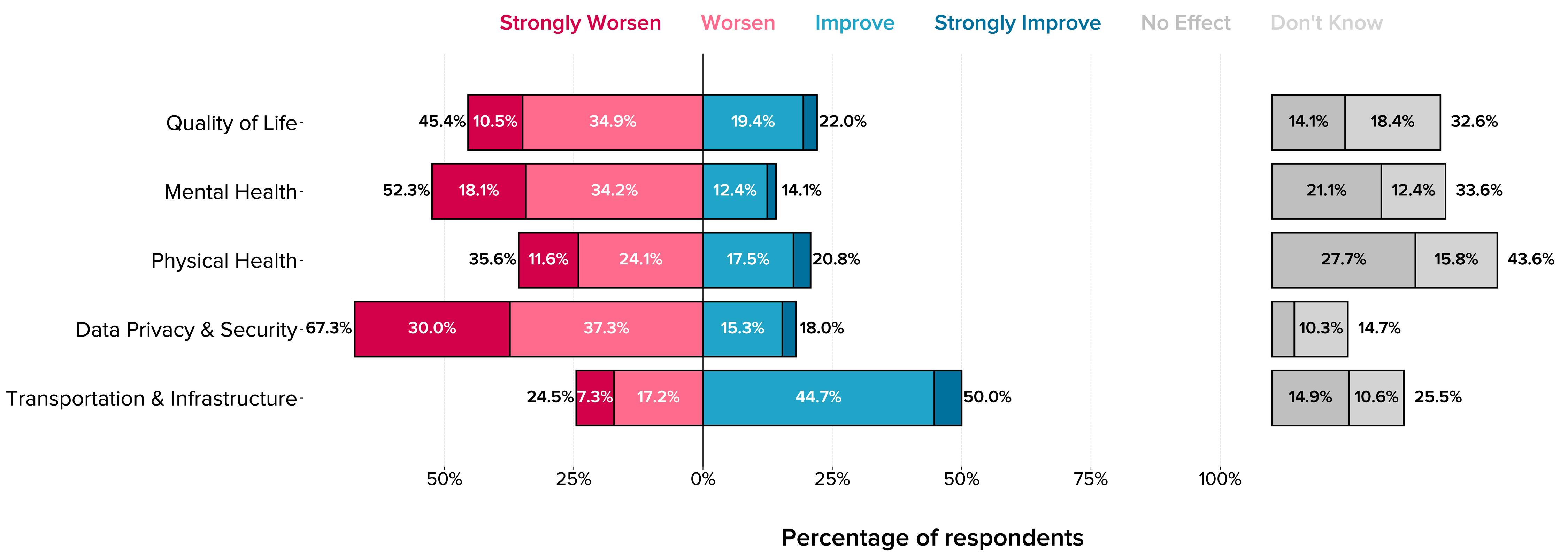

Supplement: S1.12 Fig — The figure shows unweighted relative frequencies for QS2 for the 2023 wave only. (TIFF) [file pone.0332919.s013.tif]

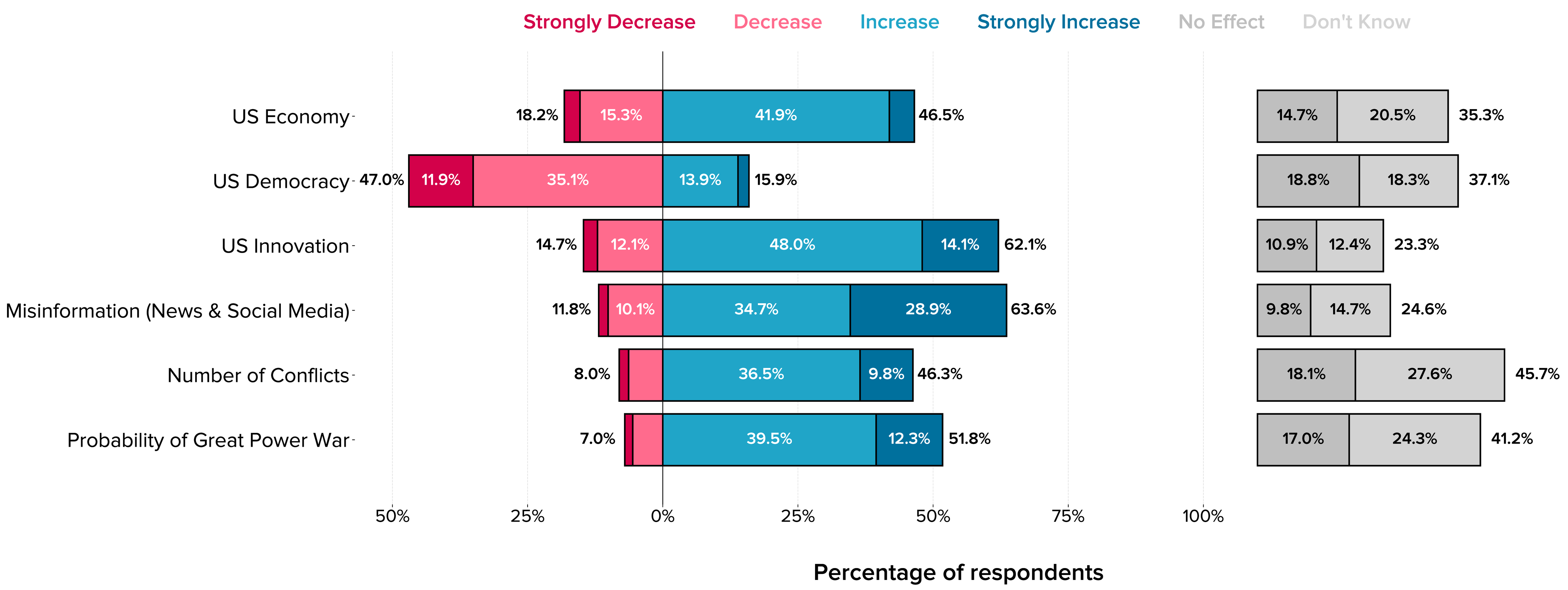

Supplement: S1.13 Fig — The figure shows unweighted relative frequencies for QS3 for the 2022 wave only. (TIFF) [file pone.0332919.s014.tif]

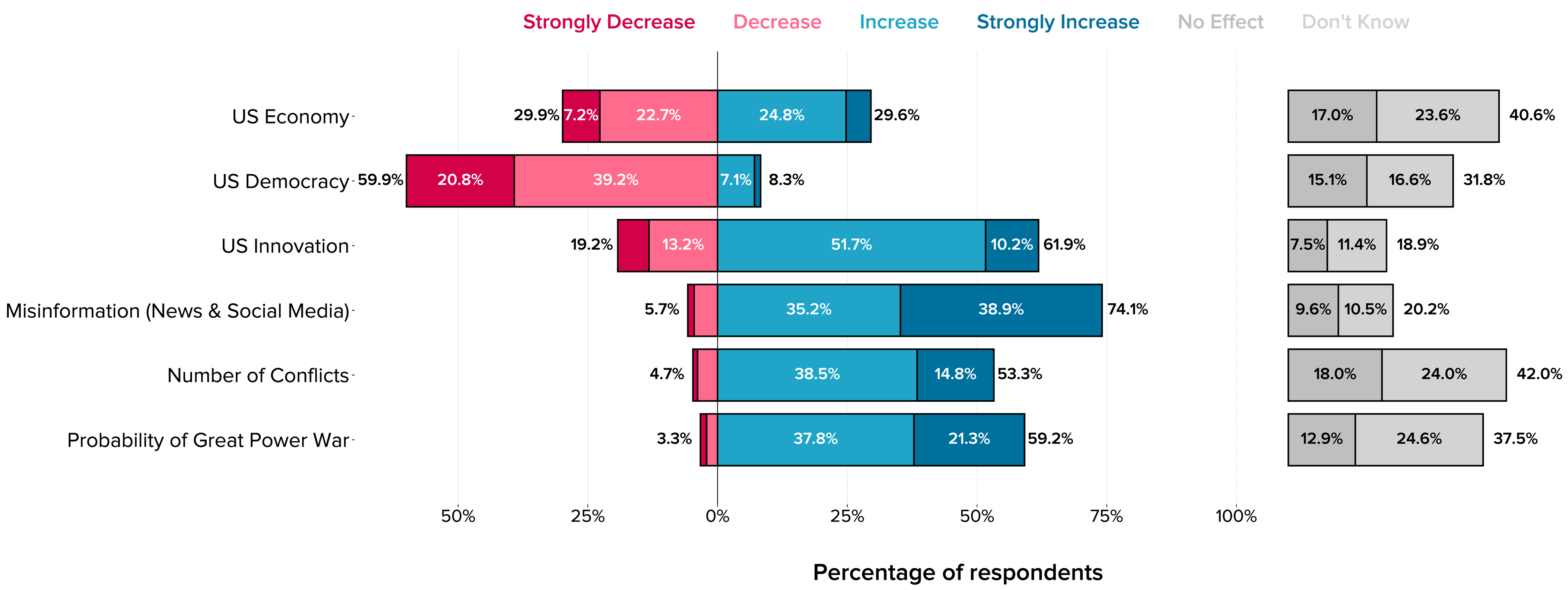

Supplement: S1.14 Fig — The figure shows unweighted relative frequencies for QS3 for the 2023 wave only. (TIFF) [file pone.0332919.s015.tif]

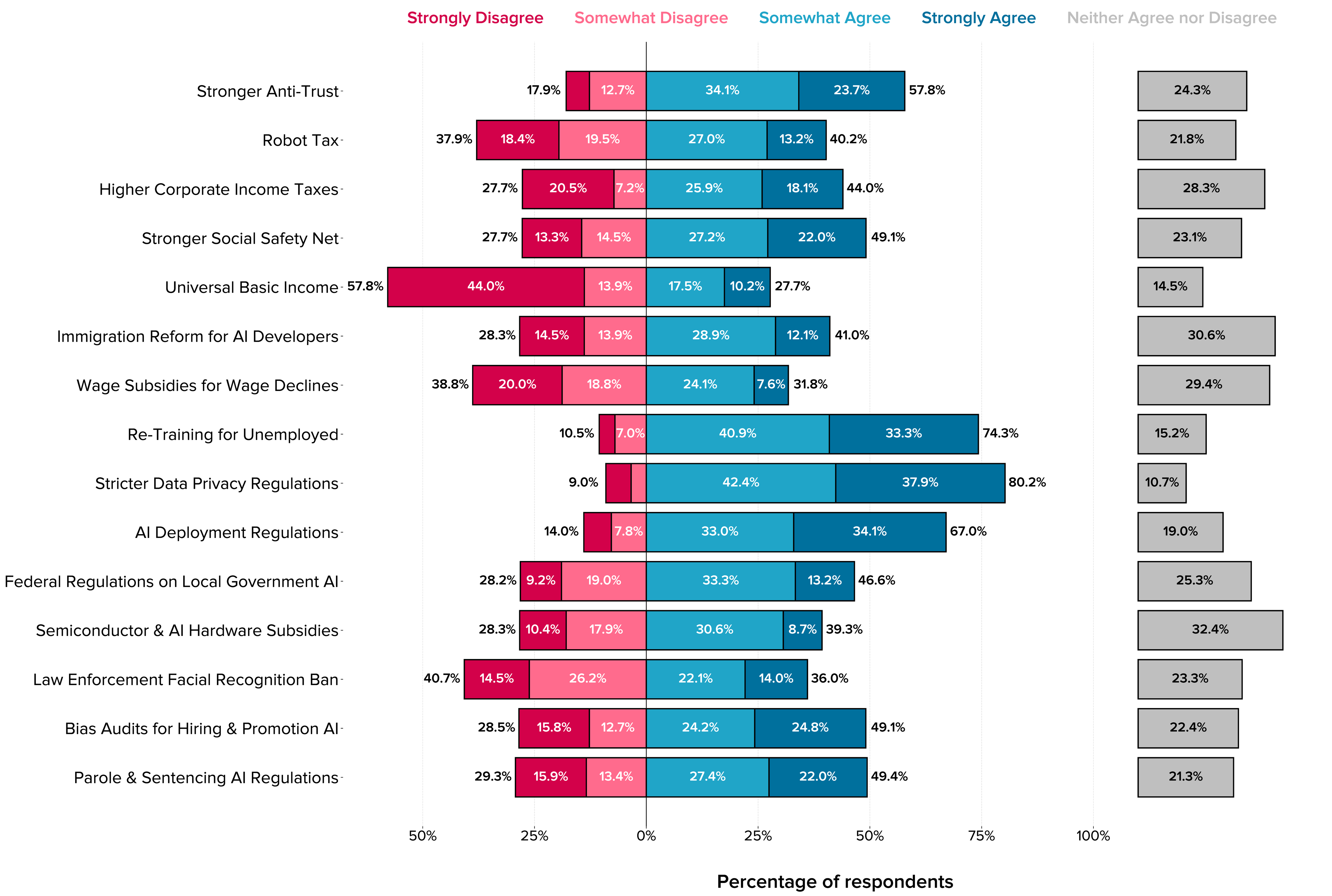

Supplement: S1.15 Fig — The figure shows unweighted relative frequencies for QS4 for the 2022 wave only. (TIFF) [file pone.0332919.s016.tif]

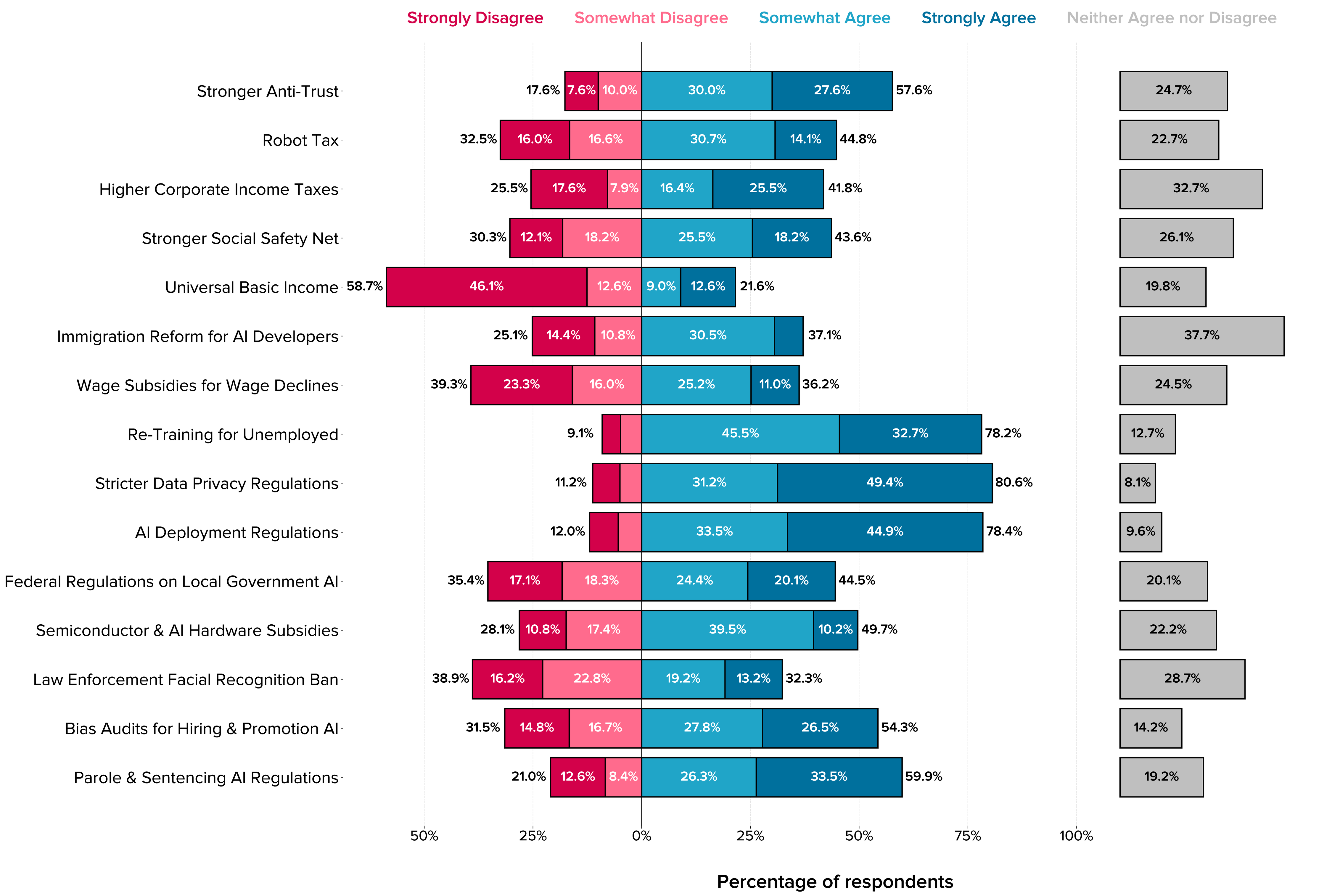

Supplement: S1.16 Fig — The figure shows unweighted relative frequencies for QS4 for the 2023 wave only. (TIFF) [file pone.0332919.s017.tif]

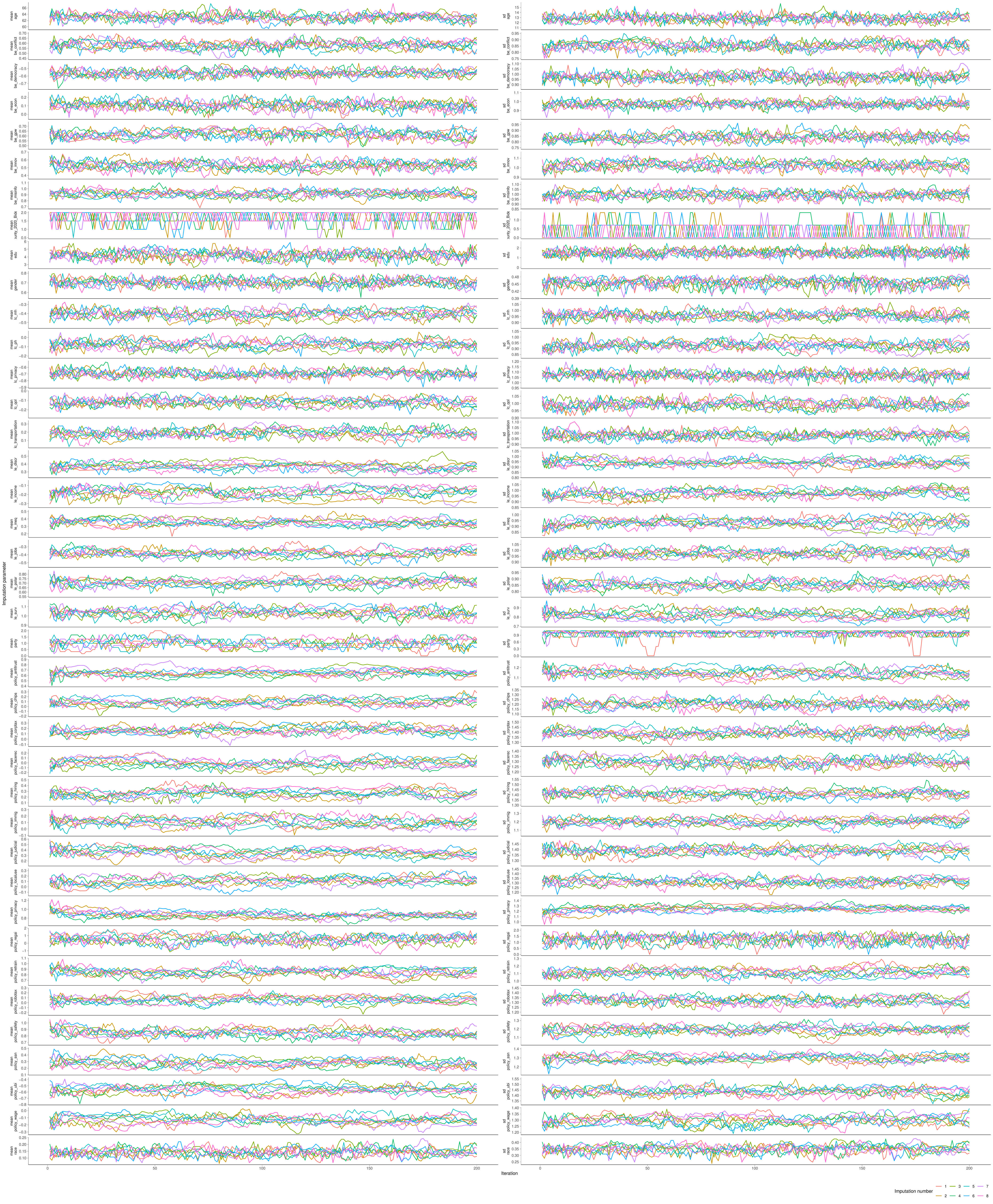

Supplement: S5.1 Fig — (TIFF) [file pone.0332919.s022.tif]

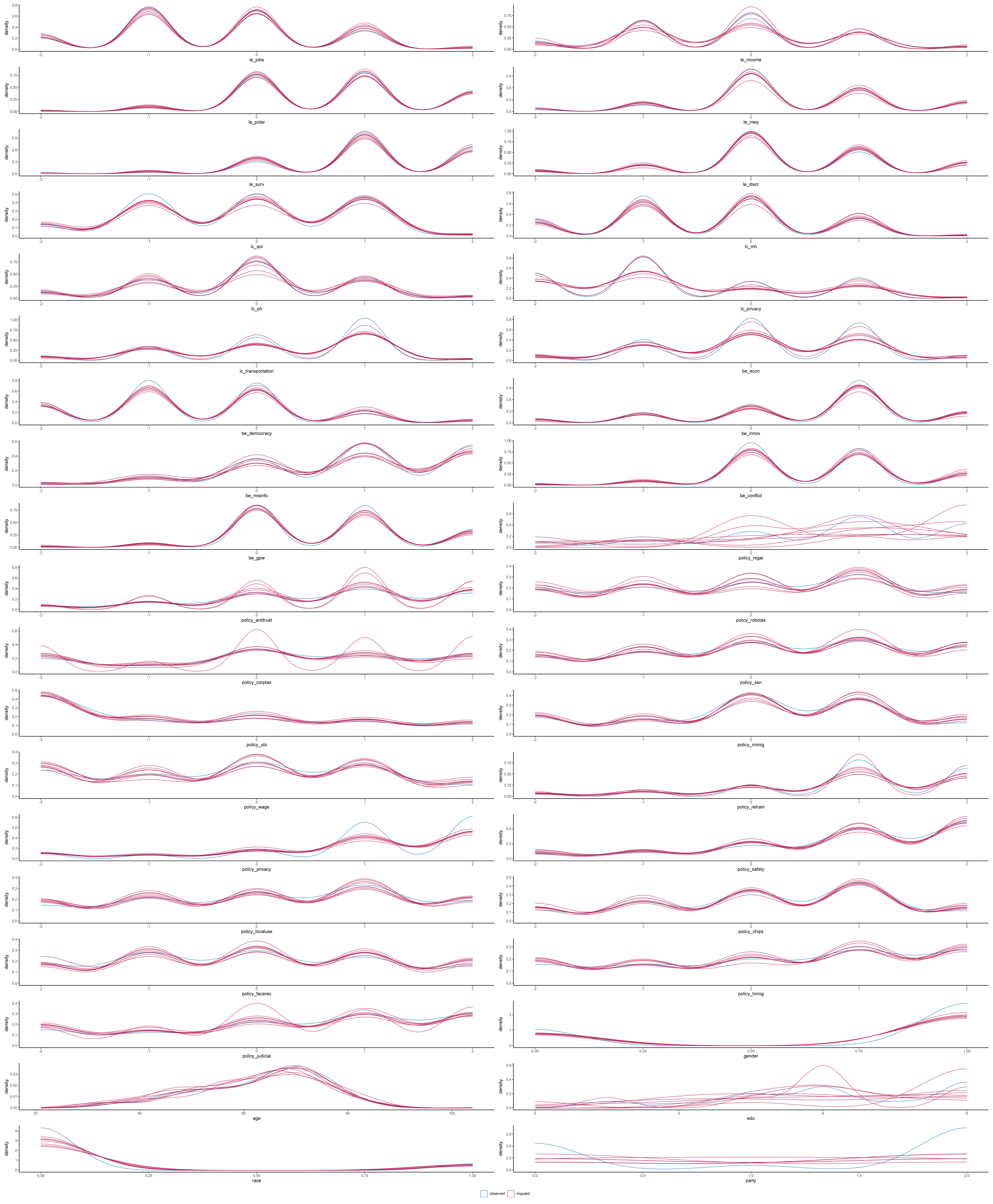

Supplement: S5.2 Fig — (TIFF) [file pone.0332919.s023.tif]

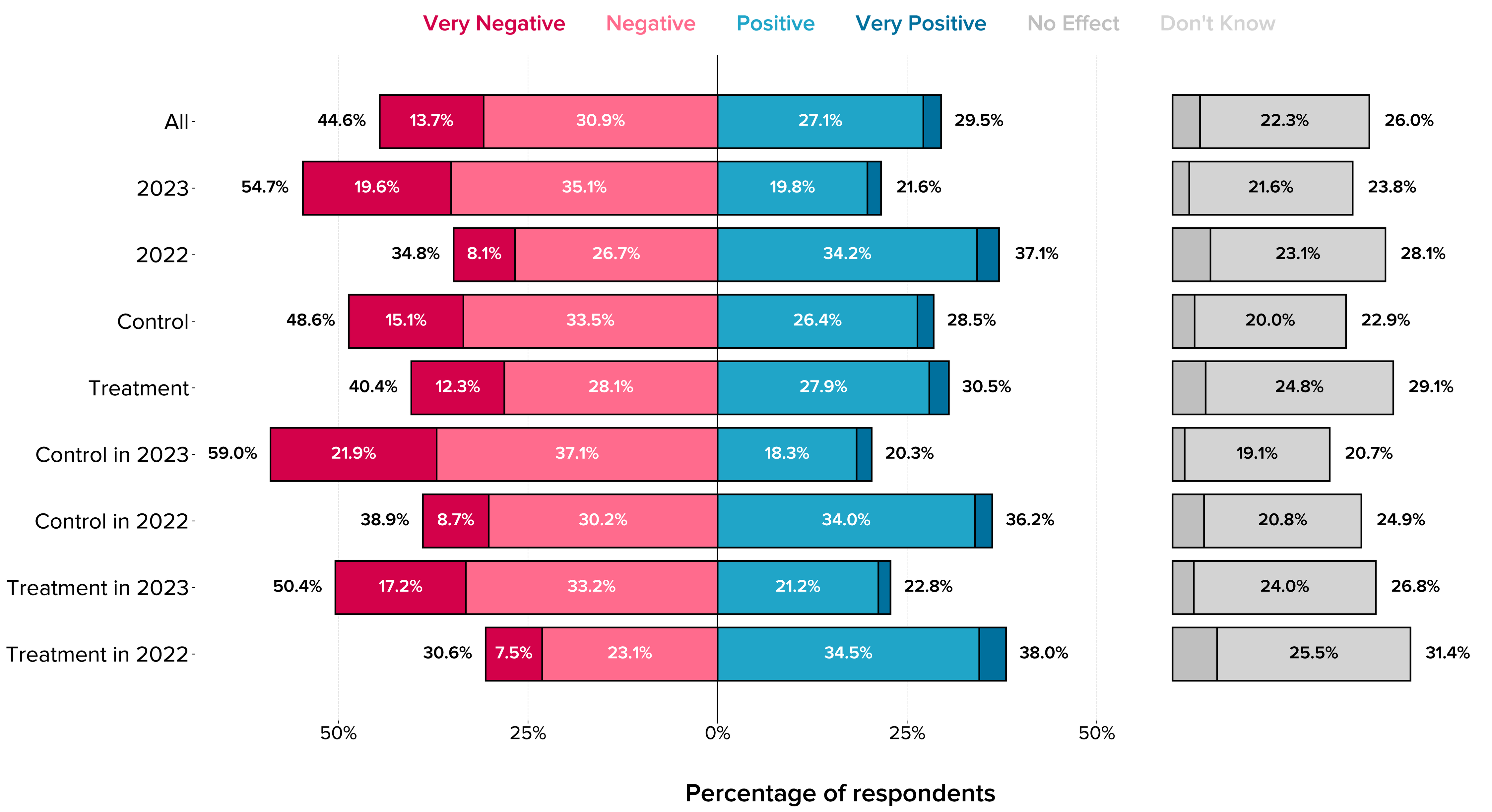

Supplement: S7.1 Fig — The figure shows unweighted relative frequencies across both survey waves. (TIFF) [file pone.0332919.s026.tif]
