# Supplementary material for: Local US officials’ views on the impacts and governance of AI: Evidence from 2022 and 2023 survey waves
Source: PLoS One. 2025 Oct 6;20(10):e0332919. doi: 10.1371/journal.pone.0332919 (PMC12500108; doi:10.1371/journal.pone.0332919)
Supplement: S2 — (PDF) [file pone.0332919.s018.pdf]

S2 Variable definitions The regression model is as follows:

$$y_i = gender + age + edu + race + party + year_{2023} + party * year_{2023} \\ + gov_{municipality} + gov_{county} + college + pop + Biden \\ + \mathbb{1}_{y_i \in \{Indices \setminus Index_{Policy\ Agreement}\}} * policy_{regai}$$

where $y_i \in \{QS1-4, Q4.1, Indices\}$, $\mathbb{1}_{y_i \in \{Indices \setminus Index_{Policy\ Agreement}\}}$ is an indicator variable for whether y_i is any index except the policy agreement index, and $policy_{regai}$ is Q4.1 (which is excluded from the regression equation for the policy agreement index as it is an element of that index).

All variables are treated as continuous unless otherwise indicated. See S3 Survey text for the text of all questions.

The independent variables in our regression model are defined as follows (coding in parentheses):

- *Gender*: self-identified gender of respondent
  - Woman (0)
  - Man (1)
  - Other / self-describe (2) (removed from regression model due to too few responses)
- *Age*: self-identified birth year cohort of respondent, treated as continuous variable by subtracting median of each bin from 2024
  - 1920 or earlier (104)
  - 1926 - 1930 (96)
  - 1931 - 1935 (91)
  - 1936 - 1940 (86)
  - 1941 - 1945 (81)
  - 1946 - 1950 (76)
  - 1951 - 1955 (71)
  - 1956 - 1960 (66)
  - 1961 - 1965 (61)
  - 1966 - 1970 (56)
  - 1971 - 1975 (51)
  - 1976 - 1980 (46)
  - 1981 - 1985 (41)
  - 1986 - 1990 (36)
  - 1991 - 1995 (31)
  - 1996 - 2000 (26)
  - 2001 - 2005 (21)
  - 2006 or later (18) ($n = 0$)
- *Edu*: self-identified education level of respondent
  - Less than high school' (0)
  - High school graduate (1)
  - Technical/trade school (2)
  - Some college (3)
  - College graduate (4)
  - Some graduate school(5)

- Graduate degree (6)

- *Race*: self-identified race of respondent
  - White (0)
  - Non-White (1)
- *Party*: political party identification of respondent, collapsed from three different questions: (1) A question asking respondent whether they think of themselves as a "Democrat"; "Republican"; "Independent"; or "Other party". (2) A question asking whether respondent thinks of themselves as closer to the "Democratic Party"; "Republican Party"; or "Neither". (3) A question asking respondent whether they think of themselves as "very conservative"; "somewhat conservative"; "moderate, middle of the road"; "somewhat liberal"; "very liberal"; or "not sure". (1) and (3) were asked of all respondents, and (2) was asked only to respondents who responded "Independent" or "Other party" to (1). Respondents who indicated in (2) that they "think of [themselves] as closer to" the Democratic or Republican parties were recoded as Democrats and Republicans, respectively. Then, respondents who "think of [themselves] as" very/somewhat liberal or as very/somewhat conservative were recoded as Democrats and Republicans, respectively. "Other party" responses to (1) were collapsed into the same category as "independent".
  - Democrat (0)
  - Independent (1)
  - Republican (2)
- $Year_{2023}$
  - 2022 (0)
  - 2023 (1)
- $Party * Year_{2023}$: the interaction term between *party* and $year_{2023}$.
- $Policy_{regai}$: response to Q4.1 ("Dreksler_AI_regulated" in the documentation). Only included for indices that were not the policy agreement index, since the policy agreement index averaged responses to all variables including Q4.1.

The following control variables in our regression model are defined below:

- *Census*: proportion of 25-years-or-older residents in respondent's geographic unit who have completed a 4-year, post-secondary degree. Data is from the 2015–2019 Five Year Data from the US Census American Community Survey, as compiled by IPUMS National Historical Geographic Information System (NHGIS). This variable is binned into terciles.
  - First tercile (0)
  - Second tercile (1)
  - Third tercile (2)
- *Pop*: total number of residents living in respondent's geographic unit. Data is from the 2015–2019 Five Year Data from the US Census American Community Survey, as compiled by IPUMS National Historical Geographic Information System (NHGIS). This variable is binned into terciles.
  - First tercile (0)
  - Second tercile (1)
  - Third tercile (2)
- *Biden*: proportion of the votes, by respondent's county, for Joe Biden in the 2020 Presidential election. Each sub-county government is matched to the relevant county in which it is contained. This variable is binned into terciles.
  - First tercile (0)
  - Second tercile (1)
  - Third tercile (2)
