# Supplementary material for: Local US officials’ views on the impacts and governance of AI: Evidence from 2022 and 2023 survey waves
Source: PLoS One. 2025 Oct 6;20(10):e0332919. doi: 10.1371/journal.pone.0332919 (PMC12500108; doi:10.1371/journal.pone.0332919)
Supplement: S3 — (PDF) [file pone.0332919.s019.pdf]

S3 Survey text The text of the survey questionnaires are contained in the files '2022 Survey draft.pdf' and '2023 Survey draft.pdf.' in the OSF repository [1] (reproduced below in S3.1 2022 Survey Text and S3.2 2023 Survey Text. The text of the substantive survey questions remained the same from 2022 to 2023, but note that our vendor (CivicPulse) made the following changes from 2022 to 2023:

- Incomplete responses: our vendor collected incomplete responses in 2022 but not in 2023. We do not report statistics on or results from incomplete responses.
- Race: note that in the original data, the race variable is binary and is coded differently from 2022 to 2023.
- Weight computation: the weights used for our regression model were computed by CivicPulse for the pooled sample according to [2] using the *gender*, *pop*, and *Biden* variables as defined in S2 Variable definitions and in the documentation.
- Census data included from 2022 to 2023: note that the 2023 dataset but not the 2022 dataset includes an *urban* variable for Census data on the proportion of the urban population in respondent's Census unit. In the original 2023 dataset, the weights were the computed using *urban*, but our regression weights were re-computed without this variable.

References

1. Dreksler N, Zhang B, Hatz S, Wei K. 2022-2023 Local US Policymaker Survey on AI; 2023. Available from: `https://osf.io/k2efj`.
2. DeBell M, Krosnick J. Computing Weights for American National Election Study Survey Data. Ann Arbor, MI, and Palo Alto, CA: American National Election Studies; 2009. nes012427. Available from: `https://electionstudies.org/wp-content/uploads/2018/04/nes012427.pdf`.

S3.1 2022 Survey Text

Spring 2022 Local Policymaker Omnibus Survey

Reference Guide for Public Access Dataset
Prepared for: Noemi Dreksler
June 13, 2022

Table of Contents

Survey Overview 4
Survey Description 4
Sample Composition 4
Survey Metadata 5
Respondent_ID 5
StartDate 5
EndDate 5
Finished 5
Weight_1 5
Weight_2 5
Dreksler_treatment 5
Questionnaire 6
Dreksler_preamble 6
Dreksler_local_effects_direct_GRID 6
Dreksler_local_effects_direct_jobs 6
Dreksler_local_effects_direct_incomes 6
Dreksler_local_effects_direct_polar 6
Dreksler_local_effects_direct_inequality 7
Dreksler_local_effects_direct_surveillance 7
Dreksler_local_effects_direct_bias 7
Dreksler_local_effects_improve_GRID 7
Dreksler_local_effects_improve_life 7
Dreksler_local_effects_improve_mental 7
Dreksler_local_effects_improve_physical 8
Dreksler_local_effects_improve_privacy 8
Dreksler_local_effects_improve_transport 8
Dreksler_broad_effects_direct_GRID 8
Dreksler_broad_effects_direct_economy 8
Dreksler_broad_effects_direct_democracy 8
Dreksler_broad_effects_direct_innovation 9
Dreksler_broad_effects_direct_misinformation 9
Dreksler_broad_effects_direct_conflicts 9
Dreksler_broad_effects_direct_greatpower 9

Dreksler_AI_regulated 9
Dreksler_broad_policy_GRID 9
Dreksler_broad_policy_antitrust 10
Dreksler_broad_policy_robot 11
Dreksler_broad_policy_corporate 11
Dreksler_broad_policy_safetynet 11
Dreksler_broad_policy_income 11
Dreksler_broad_policy_immigration 11
Dreksler_broad_policy_wage 11
Dreksler_broad_policy_training 11
Dreksler_broad_policy_privacy 11
Dreksler_broad_policy_safety 11
Dreksler_broad_policy_federal_leg 11
Dreksler_broad_policy_manufacturing 12
Dreksler_broad_policy_facialrecog 12
Dreksler_broad_policy_hiring 12
Dreksler_broad_policy_judicial 12
Dreksler_treatment_prompt 12
Dreaksler_future_effect 12
Dreksler_future_reason 12
Dreksler_future_reason_flag 12
Dreksler_local_policymaking 13
Dreksler_local_informed 13
Demographics 14
Gender 14
Age 14
Education 14
Party 14
Party_indep 14
Ideology 14
NonHispanic_white 15
Race_other_flag 15
Public Access Geographic Variables 16
Gov_level 16

State_code ... 16
State_abb ... 16
Census_area_college_bin ... 16
Census_area_population_bin ... 16
Census_area_urban_bin ... 16
County_voteshare_pres_2020_bin ... 17
Sample Representativeness ... 18

Survey Overview

Survey Description

The survey began on April 1, 2022 and was closed on May 29, 2022. The accompanying dataset includes the responses from 570 respondents. As indicated by the variable *Finished*, 524 of them completed the survey, while 46 completed part of the survey. The sample of respondents consisted of elected policymakers that were randomly drawn from U.S. local governments (i.e., township, municipality, and county governments) with a population over 1,000 residents. Elected policymakers include top elected officials and governing board members.

Sample Composition

Table 1: Government Level of Respondents

Level of Government	Number of Respondents
County	85
Municipality	352
Township	133
Total	570

Survey Metadata

Respondent_ID

A random number generated to uniquely identify each survey respondent.

StartDate

The time and date when the survey was started.

EndDate

The time and date when the survey was completed. For respondents that did not complete the survey, this variable records the time of their last activity on the survey.

Finished

‘1’ if respondent completed the survey; ‘0’ otherwise.

***Note**: We do not require survey participants to answer any particular survey question to complete the survey, though we do use “soft request” for questions left unanswered. Thus, a completed response may include unanswered questions if the respondent declined to answer.*

Weight_1

Probability weights created with a post-stratification raking procedure using the Census and presidential vote share variables. This procedure follows the methodology outlined in DeBell and Krosnick (2009) for the American National Elections Study (ANES). These weights are calculated for only the respondents who finished the survey (i.e., *Finished* = ‘1’).

Weight_2

Probability weights are created with a post-stratification raking procedure using the Census and presidential vote share variables. This procedure follows the methodology outlined in DeBell and Krosnick (2009) for the American National Elections Study (ANES). These weights are calculated for all respondents in the data set, which includes any respondent that answered at least one question.

Dreksler_treatment

This variable was randomly assigned to each respondent. Possible values are “treatment” or “control”. If *Dreksler_treatment* = “treatment” then the text in *Treatment_prompt* was shown to a respondent before the question *Future_effect*.

Questionnaire

Dreksler_preamble

The following questions will ask about the growing use of "artificial intelligence" (AI). We are interested in your thoughts as a local policymaker about the implications of AI for your community and for the country.

For the purposes of this survey, "artificial intelligence" (AI) refers to the use of computer systems to perform tasks or make decisions that usually require human intelligence. AI can perform these tasks or make these decisions without explicit human instructions.

Dreksler_local_effects_direct_GRID

*[**Instructions:** This question was displayed as a grid. Each respondent was randomly assigned 3 of the 6 rows. Response choices have been recoded using the numeric values in parenthesis. The recoded values were not displayed on the survey.]*

Please assess whether you believe AI will increase, decrease, or have no effect on each of the following outcomes for your local community in the years 2025-2050.

	Strongly decrease (-2)	Decrease (-1)	No effect (0)	Increase (1)	Strongly increase (2)	I don't know (-88)
Number of jobs						
People's incomes						
Levels of political polarization						
Inequality						
Levels of surveillance						
Bias and discrimination						

Dreksler_local_effects_direct_jobs

This variable contains responses for the row "Number of jobs" in the question *Dreksler_local_effects_direct_GRID.*

Dreksler_local_effects_direct_incomes

This variable contains responses for the row "People's incomes" in the question *Dreksler_local_effects_direct_GRID.*

Dreksler_local_effects_direct_polar

This variable contains responses for the row "Levels of political polarization" in the question *Dreksler_local_effects_direct_GRID.*

Dreksler_local_effects_direct_inequality

This variable contains responses for the row "Inequality" in the question *Dreksler_local_effects_direct_GRID.*

Dreksler_local_effects_direct_surveillance

This variable contains responses for the row "Levels of surveillance" in the question *Dreksler_local_effects_direct_GRID*.

Dreksler_local_effects_direct_bias

This variable contains responses for the row "Bias and discrimination" in the question *Dreksler_local_effects_direct_GRID*.

Dreksler_local_effects_improve_GRID

*[**Instructions:** This question was displayed as a grid. Each respondent was randomly assigned 3 of the 6 rows. Response choices have been recoded using the numeric values in parenthesis. The recoded values were not displayed on the survey.]*

Please assess whether you believe AI will improve, worsen, or have no effect on each of the following outcomes for your local community in the years 2025-2050.

	Strongly worsen (-2)	Worsen (-1)	No effect (0)	Improve (1)	Strongly Improve (2)	I don't know (-88)
Quality of life						
Mental health						
Physical health						
Data privacy and security						
Transportation and infrastructure						

Dreksler_local_effects_improve_life

This variable contains responses for the row "Quality of life" in the question *Dreksler_local_effects_improve_GRID.*

Dreksler_local_effects_improve_mental

This variable contains responses for the row "Mental health" in the question *Dreksler_local_effects_improve_GRID*.

Dreksler_local_effects_improve_physical

This variable contains responses for the row “Physical health” in the question *Dreksler_local_effects_improve_GRID*.

Dreksler_local_effects_improve_privacy

This variable contains responses for the row “Data privacy and security” in the question *Dreksler_local_effects_improve_GRID*.

Dreksler_local_effects_improve_transport

This variable contains responses for the row “Transportation and infrastructure” in the question *Dreksler_local_effects_improve_GRID*.

Dreksler_broad_effects_direct_GRID

*[**Instructions:** This question was displayed as a grid. Each respondent was randomly assigned 4 of the 6 rows. Response choices have been recoded using the numeric values in parenthesis. The recoded values were not displayed on the survey.]*

Beyond your local community, please assess whether you believe AI will increase, decrease, or have no effect on each of the following outcomes in the years 2025-2050.

	Strongly decrease (-2)	Decrease (-1)	No effect (0)	Increase (1)	Strongly increase (2)	I don’t know (-88)
Size of the US economy						
Strength of US democracy						
Rates of innovation in the US						
Amount of misinformation on US news and social media						
Number of conflicts and wars worldwide						
Likelihood of a great power war						

Dreksler_broad_effects_direct_economy

This variable contains responses for the row “Size of the US economy” in the question *Dreksler_broad_effects_direct_GRID*.

Dreksler_broad_effects_direct_democracy

This variable contains responses for the row “Strength of US democracy” in the question *Dreksler_broad_effects_direct_GRID*.

Dreksler_broad_effects_direct_innovation

This variable contains responses for the row "Rates of innovation in the US" in the question *Dreksler_broad_effects_direct_GRID*.

Dreksler_broad_effects_direct_misinformation

This variable contains responses for the row "Amount of misinformation on US news and social media" in the question *Dreksler_broad_effects_direct_GRID*.

Dreksler_broad_effects_direct_conflicts

This variable contains responses for the row "Number of conflicts and wars worldwide" in the question *Dreksler_broad_effects_direct_GRID*.

Dreksler_broad_effects_direct_greatpower

This variable contains responses for the row "Likelihood of a great power war" in the question *Dreksler_broad_effects_direct_GRID*.

Dreksler_AI_regulated

*[**Instructions:** Response choices have been recoded using the numeric values in parenthesis. The recoded values were not displayed on the survey.]*

To what extent do you agree or disagree with the following statement?

"AI should be regulated by the government."

- Strongly agree (2)
- Somewhat agree (1)
- Neither agree nor disagree (0)
- Somewhat disagree (-1)
- Strongly disagree (-2)

Dreksler_broad_policy_GRID

*[**Instructions:** This question was displayed as a grid. Each respondent was randomly assigned 5 of the 6 rows. Response choices have been recoded using the numeric values in parenthesis. The recoded values were not displayed on the survey.]*

The federal government may consider a range of policies related to artificial intelligence (AI). To what extent do you agree or disagree that the following policies would benefit the country in response to AI in the years 2025-2050?

	Strongly disagree (-2)	Somewhat Disagree (-1)	Neither agree nor disagree (0)	Somewhat agree (1)	Strongly agree (2)
Stronger anti-trust regulations					

A robot tax that taxes companies for deploying robots					
Higher corporate income taxes					
A stronger social safety net					
The federal government providing all Americans with a guaranteed income that would allow them to meet their basic needs					
Immigration reform that makes it easier to attract highly skilled AI developers					
Wage subsidies to counter wage declines					
Re-training opportunities for those at risk of unemployment due to AI and robots					
Stricter data privacy regulations					
Regulation that ensures deployed AI systems are safe, robust, and fair					
Federal legislation on how local governments use AI systems					
Subsidies for US manufacturing of semiconductors and high-end AI hardware systems					
Banning the use of facial recognition systems by law enforcement					
Requiring AI used in hiring and promotion decisions to be audited for bias					
Introducing stricter requirements for the use of AI systems in judicial decisions about sentencing and parole					

Dreksler_broad_policy_antitrust

This variable contains responses for the row “Stronger anti-trust regulations” in the question *Dreksler_broad_policy_GRID*.

Dreksler_broad_policy_robot

This variable contains responses for the row “A robot tax that taxes companies for deploying robots” in the question *Dreksler_broad_policy_GRID*.

Dreksler_broad_policy_corporate

This variable contains responses for the row “Higher corporate income taxes” in the question *Dreksler_broad_policy_GRID*.

Dreksler_broad_policy_safetynet

This variable contains responses for the row “A stronger social safety net” in the question *Dreksler_broad_policy_GRID*.

Dreksler_broad_policy_income

This variable contains responses for the row “The federal government providing all Americans with a guaranteed income that would allow them to meet their basic needs” in the question *Dreksler_broad_policy_GRID*.

Dreksler_broad_policy_immigration

This variable contains responses for the row “Immigration reform that makes it easier to attract highly skilled AI developers” in the question *Dreksler_broad_policy_GRID*.

Dreksler_broad_policy_wage

This variable contains responses for the row “Wage subsidies to counter wage declines” in the question *Dreksler_broad_policy_GRID*.

Dreksler_broad_policy_training

This variable contains responses for the row “Re-training opportunities for those at risk of unemployment due to AI and robots” in the question *Dreksler_broad_policy_GRID*.

Dreksler_broad_policy_privacy

This variable contains responses for the row “Stricter data privacy regulations” in the question *Dreksler_broad_policy_GRID*.

Dreksler_broad_policy_safety

This variable contains responses for the row “Regulation that ensures deployed AI systems are safe, robust, and fair” in the question *Dreksler_broad_policy_GRID*.

Dreksler_broad_policy_federal_leg

This variable contains responses for the row “Federal legislation on how local governments use AI systems” in the question *Dreksler_broad_policy_GRID*.

Dreksler_broad_policy_manufacturing

This variable contains responses for the row “Subsidies for US manufacturing of semiconductors and high-end AI hardware systems” in the question *Dreksler_broad_policy_GRID*.

Dreksler_broad_policy_facialrecog

This variable contains responses for the row “Banning the use of facial recognition systems by law enforcement” in the question *Dreksler_broad_policy_GRID*.

Dreksler_broad_policy_hiring

This variable contains responses for the row “Requiring AI used in hiring and promotion decisions to be audited for bias” in the question *Dreksler_broad_policy_GRID*.

Dreksler_broad_policy_judicial

This variable contains responses for the row “Introducing stricter requirements for the use of AI systems in judicial decisions about sentencing and parole” in the question *Dreksler_broad_policy_GRID*.

Dreksler_treatment_prompt

*[**Instructions:** This prompt was shown if Dreksler_treatment = “treatment”.]*

Think about children born in your community today. According to current average life expectancy rates, some of them might be alive in the year 2100.

Dreaksler_future_effect

*[**Instructions:** Response choices have been recoded using the numeric values in parenthesis. The recoded values were not displayed on the survey.]*

Do you think that AI will have an overall positive or negative effect on the US from now until 2100?

- Very positive (2)
- Positive (1)
- No effect (0)
- Negative (-1)
- Very negative (-2)
- I don’t know (-88)

Dreksler_future_reason

In 2-3 sentences, please explain why you believe AI will have this kind of impact in the long term.

Dreksler_future_reason_flag

The responses for the variable *Future_reason* were reviewed for identifying information. One responses were modified to remove specific geographic information. Those responses are marked with a ‘1’ in the variable *Future_reason*.

Dreksler_local_policymaking

*[**Instructions:** Response choices have been recoded using the numeric values in parenthesis. The recoded values were not displayed on the survey.]*

How likely is it that your local government will have to make decisions about AI-related policies and questions in the next few years?

- Have/definitely will (100% likelihood) (5)
- Highly likely (90% likelihood) (4)
- Likely (75% likelihood) (3)
- Unlikely (25% likelihood) (2)
- Highly unlikely (10% likelihood) (1)
- Definitely not (0% likelihood) (0)
- I don't know (-88)

Dreksler_local_informed

*[**Instructions:** Response choices have been recoded using the numeric values in parenthesis. The recoded values were not displayed on the survey.]*

To what extent do you agree or disagree with the following statement?

"Currently, if I had to make decisions about AI in my position, I would feel adequately informed to do so."

- Strongly agree (2)
- Somewhat agree (1)
- Neither agree nor disagree (0)
- Somewhat disagree (-1)
- Strongly disagree (-2)

Demographics

Gender

What is your gender?

- Man
- Woman
- Prefer to self-describe

Age

In what year were you born?
(1910 or earlier, 1911-1915, … , 2006 or later)

Education

What is the last grade of school you completed?

- Less than high school
- High school graduate
- Technical/trade school
- Some college
- College graduate
- Some graduate school
- Graduate degree
- Other (please specify):

Party

Generally speaking, do you usually think of yourself as a …

- Democrat
- Republican
- Independent
- Other party (please specify):

Party_indep

*[**Instructions:** This question was displayed if* Party = *"Independent" OR* Party = *"Other Party (please specify):"]*

Do you think of yourself as closer to the Democratic Party or the Republican Party?

- Democratic Party
- Republican Party
- Neither

Ideology

In general, do you think of yourself as:

- Very conservative

- Somewhat conservative
- Moderate, middle of the road
- Somewhat liberal
- Very liberal
- Not sure

NonHispanic_white

Respondents were asked their race and ethnicity using questions taken from the US Census. If a respondent identified as non-Hispanic and white, then they were coded as a ‘1’. If a person identified as Hispanic or non-white, they were coded as a ‘0’. See *Race_other_flag* for a further explanation of some of the respondents who did not identify as non-Hispanic whites.

The questions asked to construct *NonHispanic_white* are:

Are you of Hispanic, Latino, or Spanish origin?

- No, not of Hispanic, Latino, or Spanish origin
- Yes, Mexican, Mexican Am., Chicano
- Yes, Puerto Rican
- Yes, Cuban
- Yes, another Hispanic, Latino, or Spanish origin

Which of the following best describes your race/ethnicity? Please check all that apply.

- White
- Black/African American
- Asian/Asian American (includes East Asian, South Asian, Southeast Asian, and Pacific Islander)
- Native American
- Other (please specify):

Race_other_flag

Respondents were asked their race and ethnicity using questions taken from the US Census. A small minority of the respondents (less than 5 percent) identified as being “Other” non-white and specified in an open text box their preferred racial identity. In the past, some of the responses provided included, “Human,” “Why do you care?”, “Scotch-Irish and German,” “Scandinavian,” “Middle Eastern” and “Jewish.” Some of these responses may normally be categorized as “white” while some would be categorized as non-white, while yet others are not responsive to the question. The variable *Race_other_flag* was created to flag the respondents who answered “Other” so researchers could decide whether to include this group who did not identify as Non-Hispanic white, but may not be traditionally categorized as minorities.

Public Access Geographic Variables

Merging. To provide additional information about the geographic areas in which respondents work, additional covariates were merged to the survey response data (99% match rate). Where merging failed, these variables are labeled with ‘NA’.

De-identification. To ensure anonymity of the respondent, some variables were coarsened by binning them into terciles.

Gov_level

A variable indicating the level of government of the respondent. Its values are: “county,” “municipality,” and “township.”

State_code

The state in which the respondent resides provided as the two-digit FIPS code.

State_abb

The state in which the respondent resides provided as the states’ two-letter abbreviation.

Census_area_college_bin

The proportion of 25-years-or-older residents in the given geographic unit who have completed a 4-year, post-secondary degree. This data is from the 2015-2019 Five Year Data from the US Census American Community Survey, as compiled by IPUMS National Historical Geographic Information System (NHGIS). The terciles are:

- First tercile: 0 to 0.17
- Second tercile: 0.17 to 0.27
- Third tercile: 0.27 to 1.0

Census_area_population_bin

The total number of residents living in the given geographic unit. This data is from the 2015-2019 Five Year Data from the US Census American Community Survey, as compiled by IPUMS NHGIS. The terciles are:

- First tercile: 0 to 2422
- Second tercile: 2422 to 8774
- Third tercile: 8774+

Census_area_urban_bin

The proportion of residents in the given geographic unit who reside in an urban area. This data is taken the 2010 Census, as compiled by IPUMS NHGIS. The terciles are:

- First tercile: 0 to .1
- Second tercile: 0.1 to 0.96
- Third tercile: 0.96 to 1.0

County_voteshare_pres_2020_bin

The proportion of the votes, by county, for Joe Biden in the 2020 Presidential election. Each sub-county government is matched to the relevant county in which it is contained. The terciles are*:*

- First tercile: 0 to 0.31
- Second tercile: 0.31 to 0.46
- Third tercile: 0.46 to 1.0

Sample Representativeness

The tables below describe the representativeness of the sample, compared to the population. We also provide probability weights to increase sample representativeness.

Table 2: County Officials

Area Characteristics	Sample Median	Population Median
Proportion Urban	0.52	0.40
Proportion College-educated	0.21	0.19
Population Size	42,360	25,750
Democratic Vote Share 2020	0.36	0.30

Table 3: Municipality Officials

Area Characteristics	Sample Median	Population Median
Proportion Urban	0.99	0.98
Proportion College-educated	0.25	0.21
Population Size	5,030	4,180
Democratic Vote Share 2020	0.42	0.40

Table 4: Township Officials

Area Characteristics	Sample Median	Population Median
Proportion Urban	0.09	0.01
Proportion College-educated	0.27	0.22
Population Size	3,750	2,680
Democratic Vote Share 2020	0.48	0.39

S3.2 2023 Survey Text

Spring 2023 Local Policymaker Omnibus Survey

Reference Guide
Prepared for: Noemi Dreksler
June 26, 2023

The accompanying restricted dataset should only be accessed by people who have signed a Data Sharing Agreement with CivicPulse. CivicPulse has also provided a public version of the dataset that can be shared.

Table of Contents

Survey Overview 4
Survey Description 4
Sample Composition 4
Survey Metadata (Public Access) 5
Project_ID 5
StartDate 5
EndDate 5
Weight_1 5
Survey Items (Public Access) 6
Local_effects_GR 6
Local_effects_GR_jobs 6
Local_effects_GR_income 6
Local_effects_GR_polar 6
Local_effects_GR_ineq 6
Local_effects_GR_surv 6
Local_effects_GR_discr 6
Local_change_GR 7
Local_change_GR_qof 7
Local_change_GR_mh 7
Local_change_GR_ph 7
Local_change_GR_data 7
Local_change_GR_transp 7
Broad_effects_GR 7
Broad_effects_GR_econ 8
Broad_effects_GR_demo 8
Broad_effects_GR_innov 8
Broad_effects_GR_misinf 8
Broad_effects_GR_confl 8
Broad_effects_GR_war 8
Policy_regai_SC 8
Policy_GR 9
Policy_GR_antitrust 9
Policy_GR_robotax 10

Policy_GR_corptax 10
Policy_GR_socialsafety 10
Policy_GR_basicincome 10
Policy_GR_immgref 10
Policy_GR_wagsub 10
Policy_GR_retrain 10
Policy_GR_datareg 10
Policy_GR_aireg 10
Policy_GR_localuse 10
Policy_GR_semicond 10
Policy_GR_facerec 10
Policy_GR_biasaud 11
Policy_GR_juddec 11
Future_effect_SC 11
Future_reason_LOE 11
Policy_local_SC 11
Policy_informed_SC 11
Demographics (Public Access) 12
Demo_gender_SC 12
Demo_age_DD 12
Demo_education_SC 13
Demo_ethraceminority_bin 13
Demo_ideo_SC 13
Demo_party_SC 13
Demo_partylean_SC 14
Geographic Characteristics (Public Access) 15
Gov_level 15
State_code 15
State_abb 15
Census_area_college_bin 15
Census_area_population_bin 15
County_voteshare_pres_2020_bin 15
Geographic Characteristics (Restricted Access) 17
Gov_ID 17

Government_name 17
County_name 17
County_code 17
Place_code 17
Census_area_college 17
Census_area_population 17
County_voteshare_pres_2020 17
Sample Representativeness 18

Survey Overview

Survey Description

The survey began on May 9, 2023 and was closed on June 20, 2023. The accompanying dataset includes the responses from 504 respondents. The sample of respondents consisted of elected policymakers that were randomly drawn from U.S. local governments (i.e., township, municipality, and county governments) with a population over 1,000 residents. Elected policymakers include top elected officials and governing board members.

Sample Composition

Table 1: Government Level of Respondents

Level of Government	Number of Respondents
County	69
Municipality	324
Township	111
Total	504

Survey Metadata (Public Access)

Project_ID

A random number generated to uniquely identify each survey respondent.

StartDate

The time and date when the survey was started.

EndDate

The time and date when the survey was completed. For respondents that did not complete the survey, this variable records the time of their last activity on the survey.

Weight

Probability weights created with a post-stratification raking procedure using the Census and presidential vote share variables. This procedure follows the methodology outlined in DeBell and Krosnick (2009) for the American National Elections Study (ANES).

Children_prompt_EF

This variable was randomly assigned to each respondent. Possible values are “Treatment” or “Control”. If Children_prompt_EF = “Treatment” then the text in Future_treatment_TE was shown to a respondent before the question Future_effect_SC.

Survey Items (Public Access)

Local_effects_GR

[RANDOMLY PRESENT THREE ITEMS IN THE GRID BELOW, RECODE VALUES AS SHOWN IN GRID]

Please assess whether you believe AI will increase, decrease, or have no effect on each of the following outcomes for your local community in the years 2025-2050.

	Strongly decrease [-2]	Decrease [-1]	No effect [0]	Increase [1]	Strongly increase [2]	I don't know [-88]
Local_effects_GR_jobs						
Local_effects_GR_income						
Local_effects_GR_polar						
Local_effects_GR_ineq						
Local_effects_GR_surv						
Local_effects_GR_discr						

Local_effects_GR_jobs

Number of jobs

Local_effects_GR_income

People's incomes

Local_effects_GR_polar

Levels of political polarization

Local_effects_GR_ineq

Inequality

Local_effects_GR_surv

Levels of surveillance

Local_effects_GR_discr

Bias and discrimination

Local_change_GR

[RANDOMLY PRESENT THREE ITEMS IN THE GRID BELOW, RECODE VALUES AS SHOWN IN GRID]

Please assess whether you believe AI will improve, worsen, or have no effect on each of the following outcomes for your local community in the years 2025-2050.

	Strongly worsen [-2]	Worsen [-1]	No effect [0]	Improve [1]	Strongly improve [2]	I don't know [-88]
Local_change_GR_qof						
Local_change_GR_mh						
Local_change_GR_ph						
Local_change_GR_data						
Local_change_GR_transp						

Local_change_GR_qof

Quality of life

Local_change_GR_mh

Mental health

Local_change_GR_ph

Physical health

Local_change_GR_data

Data privacy and security

Local_change_GR_transp

Transportation and infrastructure

Broad_effects_GR

[RANDOMLY PRESENT FOUR ITEMS IN THE GRID BELOW, RECODE VALUES AS SHOWN IN GRID]

Beyond your local community, please assess whether you believe AI will increase, decrease, or have no effect on each of the following outcomes in the years 2025-2050.

	Strongly decrease [-2]	Decrease [-1]	No effect [0]	Increase [1]	Strongly increase [2]	I don't know [-88]
Broad_effects_GR_econ						
Broad_effects_GR_demo						
Broad_effects_GR_innov						
Broad_effects_GR_misinf						
Broad_effects_GR_confl						
Broad_effects_GR_war						

Broad_effects_GR_econ

Size of the US economy

Broad_effects_GR_demo

Strength of US democracy

Broad_effects_GR_innov

Rates of innovation in the US

Broad_effects_GR_misinf

Amount of misinformation on US news and social media

Broad_effects_GR_confl

Number of conflicts and wars worldwide

Broad_effects_GR_war

Likelihood of a great power war

Policy_regai_SC

[RECODE VALUES AS SHOWN IN BRACKETS]
To what extent do you agree or disagree with the following statement?

"AI should be regulated by the government."

- Strongly agree [2]
- Somewhat agree [1]
- Neither agree nor disagree [0]

- Somewhat disagree [-1]
- Strongly disagree [-2]

Policy_GR

The federal government may consider a range of policies related to artificial intelligence (AI). To what extent do you agree or disagree that the following policies would benefit the country in response to AI in the years 2025-2050?

[RANDOMLY PRESENT FIVE ITEMS IN THE GRID BELOW, RECODE VALUES AS SHOWN IN GRID]

	Strongly disagree [-2]	Somewhat disagree [-1]	Neither agree nor disagree [0]	Somewhat agree [1]	Strongly agree [2]
Policy_GR_antitrust					
Policy_GR_robotax					
Policy_GR_corptax					
Policy_GR_socialsafety					
Policy_GR_basicincome					
Policy_GR_immgref					
Policy_GR_wagsub					
Policy_GR_retrain					
Policy_GR_datareg					
Policy_GR_aireg					
Policy_GR_localuse					
Policy_GR_semicond					
Policy_GR_facerec					
Policy_GR_biasaud					
Policy_GR_juddec					

Policy_GR_antitrust

Stronger anti-trust regulations

Policy_GR_robotax

A robot tax that taxes companies for deploying robots

Policy_GR_corptax

Higher corporate income taxes

Policy_GR_socialsafety

A stronger social safety net

Policy_GR_basicincome

The federal government providing all Americans with a guaranteed income that would allow them to meet their basic needs

Policy_GR_immgref

Immigration reform that makes it easier to attract highly skilled AI developers

Policy_GR_wagsub

Wage subsidies to counter wage declines

Policy_GR_retrain

Re-training opportunities for those at risk of unemployment due to AI and robots

Policy_GR_datareg

Stricter data privacy regulations

Policy_GR_aireg

Regulation that ensures deployed AI systems are safe, robust, and fair

Policy_GR_localuse

Federal legislation on how local governments use AI systems

Policy_GR_semicond

Subsidies for US manufacturing of semiconductors and high-end AI hardware systems

Policy_GR_facerec

Banning the use of facial recognition systems by law enforcement

Policy_GR_biasaud

Requiring AI used in hiring and promotion decisions to be audited for bias

Policy_GR_juddec

Introducing stricter requirements for the use of AI systems in judicial decisions about sentencing and parole

Future_treatment_TE

[Displayed only when Children_prompt_EF = "Treatment"]
Think about children born in your community today. According to current average life expectancy rates, some of them might be alive in the year 2100.

Future_effect_SC

[RECODE VALUES AS SHOWN IN BRACKETS]
Do you think that AI will have an overall positive or negative effect on the US from now until 2100?

- Very positive [2]
- Positive [1]
- No effect [0]
- Negative [-1]
- Very negative [-2]
- I don't know [-88]

Future_reason_LOE

In 2-3 sentences, please explain why you believe AI will have this kind of impact in the long term.

Policy_local_SC

[RECODE VALUES AS SHOWN IN BRACKETS]
How likely is it that your local government will have to make decisions about AI-related policies and questions in the next few years?

- Have/definitely will (100% likelihood) [5]
- Highly likely (90% likelihood) [4]
- Likely (75% likelihood) [3]
- Unlikely (25% likelihood) [2]
- Highly unlikely (10% likelihood) [1]
- Definitely not (0% likelihood) [0]
- I don't know [-88]

Policy_informed_SC

[RECODE VALUES AS SHOWN IN BRACKETS]

To what extent do you agree or disagree with the following statement?

> “Currently, if I had to make decisions about AI in my position, I would feel adequately informed to do so.”

- Strongly agree [2]
- Somewhat agree [1]
- Neither agree nor disagree [0]
- Somewhat disagree [-1]
- Strongly disagree [-2]

Demographics (Public Access)

Demo_gender_SC

What is your gender?

- Man
- Woman
- Prefer to self-describe ***[Conditional display of short text box]***

Demo_age_DD

When were you born?

- 1920 or earlier
- 1921 – 1925
- 1926 – 1930
- 1931 – 1935
- 1936 – 1940
- 1941 – 1945
- 1946 – 1950
- 1951 – 1955
- 1956 – 1960
- 1961 – 1965
- 1966 – 1970
- 1971 – 1975
- 1976 – 1980
- 1981 – 1985
- 1986 – 1990
- 1991 – 1995
- 1996 – 2000
- 2001 – 2005
- 2006 or later

Demo_education_SC

What is the highest level of education you have completed?

- Less than high school
- High school graduate
- Technical/trade school
- Some college
- College graduate
- Some graduate school
- Graduate degree

Demo_ethraceminority_bin

This variable is based on responses to the question *Demo_ethrace_MS* (below). Responses are grouped to prevent identifiability. ‘1’ is assigned if a response contained “Asian/Pacific Islander”, “Black/African American”, “Hispanic/Latinx”, “Native American”, “Middle Eastern”, or “Mixed Race”. ‘0’ is assigned if “White” or “Other” was selected and NOT an additional selection.

Which of the following best describes your race/ethnicity? Please check all that apply.

- Asian/Pacific Islander
- Black/African American
- Hispanic/Latinx
- Native American
- Middle Eastern
- Mixed Race
- White
- Prefer to self-describe ***[Conditional display of short text box]***

Demo_ideo_SC

In general, do you think of yourself as:

- Very conservative
- Somewhat conservative
- Moderate, middle of the road
- Somewhat liberal
- Very liberal
- Not sure

Demo_party_SC

Generally speaking, do you usually think of yourself as a…

- Democrat
- Republican
- Independent
- Other party ***[Conditional display of short text box]***

Demo_partylean_SC

Do you think of yourself as closer to the Democratic Party or the Republican Party?

- Democratic Party
- Republican Party
- Neither

Geographic Characteristics (Public Access)

Merging. To provide additional information about the geographic areas in which respondents work, additional covariates were merged to the survey response data (99% match rate). Where merging failed, these variables are labeled with ‘NA’.

De-identification. To ensure anonymity of the respondent, some variables were coarsened by binning them into terciles.

Gov_level

A variable indicating the level of government of the respondent. Its values are: “county,” “municipality,” and “township.”

State_code

The state in which the respondent resides provided as the two-digit FIPS code.

State_abb

The state in which the respondent resides provided as the states’ two-letter abbreviation.

Census_area_college_bin

The proportion of 25-years-or-older residents in the given geographic unit who have completed a 4-year, post-secondary degree. This data is from the 2015-2019 Five Year Data from the US Census American Community Survey, as compiled by IPUMS National Historical Geographic Information System (NHGIS). The terciles are:

- First tercile: 0 to 0.17
- Second tercile: 0.17 to 0.27
- Third tercile: 0.27 to 1.0

Census_area_population_bin

The total number of residents living in the given geographic unit. This data is from the 2015-2019 Five Year Data from the US Census American Community Survey, as compiled by IPUMS NHGIS. The terciles are:

- First tercile: 0 to 2422
- Second tercile: 2422 to 8774
- Third tercile: 8774+

County_voteshare_pres_2020_bin

The proportion of the votes, by county, for Joe Biden in the 2020 Presidential election. Each sub-county government is matched to the relevant county in which it is contained. The terciles are*:*

- First tercile: 0 to 0.31
- Second tercile: 0.31 to 0.46
- Third tercile: 0.46 to 1.0

Geographic Characteristics (Restricted Access)

Gov_ID

This ID corresponds to the CensusID in the US Census's Census of Governments.

Government_name

The name of a respondent's government jurisdiction.

County_name

The county name where a government is located. (For county governments, this is the same name as the government.)

County_code

The three-digit County FIPS code corresponding to the county where a government is located.

Place_code

The two-digit Place FIPS code corresponding to a government's jurisdiction. This is NA for county governments.

Census_area_college

The proportion of 25-years-or-older residents in the given geographic unit who have completed a 4-year, post-secondary degree. This data is from the 2015-2019 Five Year Data from the US Census American Community Survey, as compiled by IPUMS National Historical Geographic Information System (NHGIS). This corresponds to Census_area_college_bin.

Census_area_population

The total number of residents living in the given geographic unit. This data is from the 2015-2019 Five Year Data from the US Census American Community Survey, as compiled by IPUMS NHGIS. This corresponds to Census_area_population_bin.

County_voteshare_pres_2020

The proportion of the votes, by county, for Joe Biden in the 2020 Presidential election. Each sub-county government is matched to the relevant county in which it is contained. This corresponds to County_voteshare_pres_2020_bin.

Sample Representativeness

The tables below describe the representativeness of the sample, compared to the population. We also provide probability weights to increase sample representativeness.

Table 1: County Officials

Area Characteristics	Sample Median	Population Median
Proportion College-educated	0.22	0.19
Population Size	42,360	25,750
Democratic Vote Share 2020	0.35	0.30

Table 2: Municipality Officials

Area Characteristics	Sample Median	Population Median
Proportion College-educated	0.25	0.21
Population Size	5,080	4,180
Democratic Vote Share 2020	0.43	0.40

Table 3: Township Officials

Area Characteristics	Sample Median	Population Median
Proportion College-educated	0.27	0.22
Population Size	3,900	2,680
Democratic Vote Share 2020	0.48	0.39
