# Supplementary material for: Local US officials’ views on the impacts and governance of AI: Evidence from 2022 and 2023 survey waves
Source: PLoS One. 2025 Oct 6;20(10):e0332919. doi: 10.1371/journal.pone.0332919 (PMC12500108; doi:10.1371/journal.pone.0332919)
Supplement: S4 — (PDF) [file pone.0332919.s020.pdf]

S4 Indices definitions We created respondent-level indices on which we fit our regression model. These indices are defined below using the variable definitions from S3 Survey text (original variable names in parentheses):

- Policy agreement index: average of all policy questions (Q4.1–QS4)
- Positive impacts index: average of
  - Number of jobs (Local_effects_GR_jobs)
  - People's incomes (Local_effects_GR_income)
  - Quality of life (Local_change_GR_qof)
  - Mental health (Local_change_GR_mh)
  - Physical health (Local_change_GR_ph)
  - Data privacy and security (Local_change_GR_data)
  - Transportation and infrastructure (Local_change_GR_transp)
  - Size of the US economy (Broad_effects_GR_econ)
  - Strength of US democracy (Broad_effects_GR_demo)
  - Rates of innovation in the US (Broad_effects_GR_innov)
- Negative impacts index: average of
  - Levels of political polarization (Local_effects_GR_polar)
  - Inequality (Local_effects_GR_ineq)
  - Levels of surveillance (Local_effects_GR_surv)
  - Bias and discrimination (Local_effects_GR_discr)
  - Amount of misinformation on US news and social media (Broad_effects_GR_misinf)
  - Number of conflicts and wars worldwide (Broad_effects_GR_confl)
  - Likelihood of a great power war (Broad_effects_GR_war)
- Economic impacts index: average of
  - Number of jobs (Local_effects_GR_jobs)
  - People's incomes (Local_effects_GR_income)
  - Inequality (Local_effects_GR_ineq)
  - Size of the US economy (Broad_effects_GR_econ)
- Societal impacts index: average of
  - Levels of surveillance (Local_effects_GR_surv)
  - Bias and discrimination (Local_effects_GR_discr)
  - Data privacy and security (Local_change_GR_data)
  - Amount of misinformation on US news and social media (Broad_effects_GR_misinf)
- Personal well-being & community health index: average of
  - Quality of life (Local_change_GR_qof)
  - Mental health (Local_change_GR_mh)
  - Physical health (Local_change_GR_ph)
- Progress and innovation impacts index: average of
  - Transportation and infrastructure (Local_change_GR_transp)
  - Rates of innovation in the US (Broad_effects_GR_innov)

- Political impacts index: average of
  - Levels of political polarization (Local_effects_GR_polar)
  - Strength of US democracy (Broad_effects_GR_demo)
  - Number of conflicts and wars worldwide (Broad_effects_GR_confl)
  - Likelihood of a great power war (Broad_effects_GR_war)
- Positive local community impacts index: average of
  - Number of jobs (Local_effects_GR_jobs)
  - People's incomes (Local_effects_GR_income)
  - Quality of life (Local_change_GR_qof)
  - Mental health (Local_change_GR_mh)
  - Physical health (Local_change_GR_ph)
  - Data privacy and security (Local_change_GR_data)
  - Transportation and infrastructure (Local_change_GR_transp)
- Negative local community impacts index: average of
  - Levels of political polarization (Local_effects_GR_polar)
  - Inequality (Local_effects_GR_ineq)
  - Levels of surveillance (Local_effects_GR_surv)
  - Bias and discrimination (Local_effects_GR_discr)
- Positive broad impacts index: average of
  - Size of the US economy (Broad_effects_GR_econ)
  - Strength of US democracy (Broad_effects_GR_demo)
  - Rates of innovation in the US (Broad_effects_GR_innov)
- Negative broad impacts index: average of
  - Amount of misinformation on US news and social media (Broad_effects_GR_misinf)
  - Number of conflicts and wars worldwide (Broad_effects_GR_confl)
  - Likelihood of a great power war (Broad_effects_GR_war)
