# Supplementary material for: Local US officials’ views on the impacts and governance of AI: Evidence from 2022 and 2023 survey waves
Source: PLoS One. 2025 Oct 6;20(10):e0332919. doi: 10.1371/journal.pone.0332919 (PMC12500108; doi:10.1371/journal.pone.0332919)
Supplement: S5 — (PDF) [file pone.0332919.s021.pdf]

S5 Full Regression Results Tables S1.1–S1.5 contain the full results of the survey-weighted linear regression model as specified in Materials and Methods, across both survey waves. P-values are Benjamini-Hochberg adjusted, and IDK responses are re-coded as neutral (0) where applicable. The results in this section are the full version of the truncated results presented in Results.

We also present figures to assess the fit of our imputations. Fig S1.1 contains trace plots for imputed parameters across 200 iterations. We display only eight randomly selected imputed datasets for readability (our procedure imputed 120 datasets). Generally, convergence appears to occur for all parameters except the *Biden_county_*2020 and *party* variables; however, missingness for these variables are extremely low since the first is Census-level data and the latter is combined from three different fields, so our results should not be implicated. Fig S1.2 display density plots for the same eight imputed datasets layered on top of the original sample; for the vast majority of variables, the imputations look to map well onto the original distribution.

S6 Alternative Regression Results contains the full results for the same model specification but with IDK responses imputed where applicable.

Question[a]				Covariate			
	Age	*Education*	*Gender*[b]	*Race*[c]	*Party*[d]	*Year*[e]	*Party ∗ Year*
**QS1: Local Effects of AI**							
Number of Jobs	0.006	-0.002	0.137	0.077	-0.037	-0.311	-0.007
	(-0.001, 0.013)	(-0.057, 0.054)	(-0.045, 0.32)	(-0.168, 0.322)	(-0.144, 0.069)	(-0.537, -0.085)	(-0.149, 0.135)
Income Level	0.004	-0.057	-0.011	-0.065	-0.027	-0.295	0.013
	(-0.003, 0.01)	(-0.11, -0.004)	(-0.194, 0.171)	(-0.321, 0.192)	(-0.136, 0.081)	(-0.518, -0.072)	(-0.131, 0.157)
Political Polarization	-0.003	0.013	-0.014	-0.008	-0.021	0.137	-0.006
	(-0.008, 0.003)	(-0.034, 0.06)	(-0.182, 0.155)	(-0.237, 0.22)	(-0.122, 0.08)	(-0.079, 0.354)	(-0.14, 0.128)
Inequality	0.003	0.006	-0.118	0.105	-0.047	0.066	0.001
	(-0.003, 0.009)	(-0.042, 0.054)	(-0.31, 0.075)	(-0.146, 0.356)	(-0.152, 0.058)	(-0.162, 0.294)	(-0.137, 0.14)
Surveillance Level	**-0.010****	**0.095*****	0.047	0.121	0.030	-0.005	0.017
	**(-0.016, -0.005)**	**(0.051, 0.138)**	(-0.113, 0.207)	(-0.078, 0.32)	(-0.068, 0.128)	(-0.198, 0.189)	(-0.107, 0.141)
Bias & Discrimination	0.007	-0.012	-0.199	0.161	-0.001	0.194	-0.021
	(-0.0, 0.013)	(-0.064, 0.04)	(-0.387, -0.012)	(-0.075, 0.398)	(-0.114, 0.112)	(-0.031, 0.42)	(-0.165, 0.124)
**QS2: Local Effects of AI**							
Quality of Life	-0.003	0.054	**0.263***	-0.161	**-0.161***	-0.274	0.033
	(-0.009, 0.004)	(0.009, 0.099)	**(0.094, 0.432)**	(-0.396, 0.073)	**(-0.263, -0.058)**	(-0.486, -0.061)	(-0.106, 0.173)
Mental Health	0.003	0.049	**0.313****	0.111	-0.137	-0.220	0.066
	(-0.004, 0.009)	(0.005, 0.093)	**(0.149, 0.478)**	(-0.125, 0.347)	(-0.235, -0.039)	(-0.441, 0.0)	(-0.073, 0.205)
Physical Health	0.001	0.054	0.188	0.119	**-0.181****	-0.159	0.050
	(-0.005, 0.008)	(0.005, 0.103)	(0.01, 0.365)	(-0.107, 0.345)	**(-0.278, -0.085)**	(-0.366, 0.047)	(-0.082, 0.183)
Data Privacy &	-0.002	0.042	0.091	0.246	-0.014	-0.077	-0.105
Security	(-0.009, 0.006)	(-0.011, 0.095)	(-0.107, 0.288)	(-0.025, 0.518)	(-0.132, 0.104)	(-0.327, 0.173)	(-0.264, 0.053)
Transportation &	-0.001	**0.121*****	0.168	0.023	-0.084	0.092	-0.068
Infrastructure	(-0.007, 0.005)	**(0.075, 0.167)**	(-0.012, 0.348)	(-0.194, 0.241)	(-0.18, 0.011)	(-0.103, 0.287)	(-0.202, 0.067)

Table S1.1. Full Results from Regression Analysis for QS1–2.[f]

* = $p < 0.05$, ** = $p < 0.01$, *** = $p < 0.001$. All statistically significant results in bold.

[a] For each question, higher values represent belief that outcomes would increase.

[b] 0 = woman and 1 = man.

[c] 0 = white, 1 = non-white.

[d] 0 = Democrat, 1 = independent or other party, and 2 = Republican.

[e] 0 = 2022 and 1 = 2023.

[f] Results are for the survey-weighted linear regression model as specified in Materials and Methods, across both survey waves. P-values are Benjamini-Hochberg adjusted. IDK responses are re-coded as neutral (0).

**Question**[a]	*Age*	*Education*	*Gender*[b]	**Covariate** *Race*[c]	*Party*[d]	*Year*[e]	*Party ∗ Year*
**QS3: Broad Effects of AI**							
US Economy	0.007	0.019	**0.246***	0.157	-0.104	-0.269	-0.031
	(0.001, 0.013)	(-0.028, 0.066)	**(0.077, 0.414)**	(-0.057, 0.37)	(-0.203, -0.005)	(-0.475, -0.063)	(-0.166, 0.103)
US Democracy	0.005	-0.000	0.083	0.238	-0.099	-0.281	0.033
	(-0.001, 0.012)	(-0.047, 0.046)	(-0.083, 0.248)	(0.015, 0.461)	(-0.204, 0.006)	(-0.498, -0.065)	(-0.108, 0.173)
US Innovation	-0.001	0.052	0.214	0.076	-0.051	0.000	-0.063
	(-0.008, 0.005)	(0.002, 0.101)	(0.032, 0.397)	(-0.151, 0.302)	(-0.158, 0.055)	(-0.22, 0.221)	(-0.209, 0.084)
Misinformation	-0.008	-0.014	-0.061	-0.040	0.043	0.298	-0.045
(News & Social Media)	(-0.014, -0.002)	(-0.06, 0.033)	(-0.234, 0.111)	(-0.289, 0.209)	(-0.072, 0.157)	(0.061, 0.535)	(-0.192, 0.103)
Number of Conflicts	-0.003	-0.048	-0.153	-0.025	0.029	0.105	0.019
	(-0.008, 0.002)	(-0.089, -0.008)	(-0.303, -0.002)	(-0.218, 0.167)	(-0.064, 0.123)	(-0.086, 0.297)	(-0.102, 0.14)
Probability of	-0.006	**-0.071***	-0.058	-0.017	-0.012	0.109	0.045
Great Power War	(-0.012, -0.001)	**(-0.113, -0.03)**	(-0.207, 0.09)	(-0.219, 0.185)	(-0.107, 0.082)	(-0.078, 0.295)	(-0.076, 0.167)

Table S1.2. Full Results from Regression Analysis for QS3.[f]

* = $p < 0.05$, ** = $p < 0.01$, *** = $p < 0.001$. All statistically significant results in bold.

[a] For each question, higher values represent belief that outcomes would increase.

[b] 0 = woman and 1 = man.

[c] 0 = white, 1 = non-white.

[d] 0 = Democrat, 1 = independent or other party, and 2 = Republican.

[e] 0 = 2022 and 1 = 2023.

[f] Results are for the survey-weighted linear regression model as specified in Materials and Methods, across both survey waves. P-values are Benjamini-Hochberg adjusted. Respondents did not have the IDK option for QS3.

Question[a]	*Age*	*Education*	*Gender*[b]	Covariate *Race*[c]	*Party*[d]	*Year*[e]	$Party * Year$
**QS4.1: General Support for AI Regulation**							
Support AI Regulation	-0.003	-0.007	0.056	-0.265	**-0.363*****	**0.374****	0.142
	(-0.009, 0.004)	(-0.057, 0.044)	(-0.126, 0.238)	(-0.505, -0.025)	**(-0.473, -0.252)**	**(0.178, 0.569)**	(-0.0, 0.284)

Table S1.3. Full Results from Regression Analysis for Q4.1.[f]

* = $p < 0.05$, ** = $p < 0.01$, *** = $p < 0.001$. All statistically significant results in bold.

[a] For each question, higher values represent belief that outcomes would increase.

[b] 0 = woman and 1 = man.

[c] 0 = white, 1 = non-white.

[d] 0 = Democrat, 1 = independent or other party, and 2 = Republican.

[e] 0 = 2022 and 1 = 2023.

[f] Results are for the survey-weighted linear regression model as specified in Materials and Methods, across both survey waves. P-values are Benjamini-Hochberg adjusted. Respondents did not have the IDK option for QS4.1.

Question[a]	*Age*	*Education*	*Gender*[b]	Covariate<br>*Race*[c]	*Party*[d]	*Year*[e]	*Party ∗ Year*
**QS4: Policy Support**							
Stronger Anti-Trust	0.004	0.033	-0.087	-0.234	**-0.359*****	-0.016	0.080
	(-0.005, 0.013)	(-0.034, 0.101)	(-0.319, 0.145)	(-0.546, 0.078)	**(-0.49, -0.228)**	(-0.272, 0.24)	(-0.086, 0.246)
Robot Tax	-0.002	0.007	**-0.552***	-0.296	**-0.298***	0.078	0.068
	(-0.014, 0.01)	(-0.078, 0.092)	**(-0.875, -0.229)**	(-0.686, 0.095)	**(-0.472, -0.123)**	(-0.259, 0.415)	(-0.14, 0.275)
Higher Corporate	-0.011	0.003	-0.257	0.112	**-0.659*****	0.039	-0.004
Income Taxes	(-0.021, -0.001)	(-0.076, 0.081)	(-0.539, 0.025)	(-0.256, 0.479)	**(-0.821, -0.497)**	(-0.268, 0.346)	(-0.197, 0.188)
Stronger Social Safety Net	0.009	-0.016	-0.146	-0.053	**-0.555*****	0.070	0.024
	(0.0, 0.019)	(-0.091, 0.06)	(-0.411, 0.119)	(-0.394, 0.288)	**(-0.699, -0.412)**	(-0.223, 0.363)	(-0.159, 0.207)
Universal Basic Income	-0.013	0.086	-0.087	0.428	**-0.859*****	-0.062	0.042
	(-0.022, -0.004)	(0.018, 0.154)	(-0.337, 0.163)	(0.037, 0.818)	**(-1.015, -0.703)**	(-0.397, 0.274)	(-0.138, 0.221)
Immigration Reform for	0.005	-0.003	0.250	-0.079	**-0.317****	-0.132	0.062
AI Developers	(-0.004, 0.015)	(-0.078, 0.072)	(-0.004, 0.504)	(-0.421, 0.262)	**(-0.482, -0.151)**	(-0.492, 0.229)	(-0.142, 0.265)
Wage Subsidies for	0.001	-0.039	-0.296	-0.157	**-0.642*****	0.057	-0.016
Wage Declines	(-0.009, 0.01)	(-0.113, 0.035)	(-0.534, -0.059)	(-0.493, 0.179)	**(-0.79, -0.494)**	(-0.234, 0.349)	(-0.194, 0.162)
Re-Training for Unemployed	0.003	0.014	0.134	-0.079	**-0.281****	-0.007	0.045
	(-0.006, 0.011)	(-0.051, 0.078)	(-0.097, 0.365)	(-0.393, 0.235)	**(-0.422, -0.139)**	(-0.279, 0.264)	(-0.122, 0.213)
Stricter Data Privacy Regulations	-0.004	**0.116****	-0.112	-0.088	-0.186	0.055	0.041
	(-0.013, 0.006)	**(0.049, 0.184)**	(-0.363, 0.139)	(-0.424, 0.249)	(-0.329, -0.043)	(-0.209, 0.32)	(-0.14, 0.223)
AI Deployment Regulations	0.001	0.039	-0.208	-0.282	**-0.307*****	0.113	0.109
	(-0.01, 0.011)	(-0.037, 0.115)	(-0.452, 0.036)	(-0.638, 0.073)	**(-0.446, -0.168)**	(-0.14, 0.366)	(-0.065, 0.283)
Federal Regulations on	-0.005	0.044	-0.128	0.153	**-0.320****	-0.130	0.071
Local Government AI	(-0.015, 0.004)	(-0.036, 0.123)	(-0.416, 0.16)	(-0.199, 0.505)	**(-0.491, -0.149)**	(-0.463, 0.203)	(-0.123, 0.265)
Semiconductor &	0.008	0.004	0.104	-0.172	**-0.260***	0.274	-0.056
& AI Hardware Subsidies	(-0.002, 0.018)	(-0.074, 0.083)	(-0.181, 0.389)	(-0.57, 0.227)	**(-0.419, -0.101)**	(-0.026, 0.574)	(-0.25, 0.138)
Law Enforcement	-0.006	0.007	-0.226	0.155	-0.131	-0.088	0.029
Facial Recognition Ban	(-0.017, 0.005)	(-0.076, 0.089)	(-0.538, 0.086)	(-0.257, 0.566)	(-0.314, 0.052)	(-0.438, 0.262)	(-0.182, 0.24)
Bias Audits for Hiring &	-0.009	0.052	-0.209	-0.084	**-0.386*****	0.030	0.075
Promotion AI	(-0.02, 0.002)	(-0.033, 0.137)	(-0.55, 0.133)	(-0.47, 0.302)	**(-0.561, -0.211)**	(-0.31, 0.369)	(-0.141, 0.29)
Parole & Sentencing	-0.011	0.084	0.177	-0.008	**-0.397*****	0.305	0.051
AI Regulations	(-0.024, 0.001)	(-0.006, 0.173)	(-0.134, 0.488)	(-0.408, 0.392)	**(-0.576, -0.219)**	(-0.052, 0.662)	(-0.164, 0.267)

Table S1.4. Full Results from Regression Analysis for QS4.[f]

* = $p < 0.05$, ** = $p < 0.01$, *** = $p < 0.001$. All statistically significant results in bold.

[a] For each question, higher values represent belief that outcomes would increase.

[b] 0 = woman and 1 = man.

[c] 0 = white, 1 = non-white.

[d] 0 = Democrat, 1 = independent or other party, and 2 = Republican.

[e] 0 = 2022 and 1 = 2023.

[f] Results are for the survey-weighted linear regression model as specified in Materials and Methods, across both survey waves. P-values are Benjamini-Hochberg adjusted. Respondents did not have the IDK option for QS4.

Question[a]	*Age*	*Education*	*Gender*[b]	*Race*[c]	*Party*[d]	*Year*[e]	*Party ∗ Year*	$Policy_{RegAI}$
	Covariate							
**Constructed Indices**								
Policy Agreement	-0.002	0.019	-0.092	-0.082	**-0.400*****	0.106	0.056	–
	(-0.006, 0.002)	(-0.014, 0.051)	(-0.206, 0.021)	(-0.239, 0.075)	**(-0.470, -0.330)**	(-0.028, 0.239)	(-0.038, 0.150)	
Positive Impacts (All)	0.002	0.036	**0.208****	0.087	-0.089	-0.185	-0.010	0.018
	(-0.002, 0.006)	(0.006, 0.066)	**(0.1, 0.317)**	(-0.057, 0.232)	(-0.156, -0.022)	(-0.321, -0.049)	(-0.1, 0.081)	(-0.026, 0.062)
Negative Impacts (All)	-0.004	-0.008	-0.084	0.047	0.029	0.105	0.003	0.046
	(-0.007, -0.0)	(-0.037, 0.021)	(-0.194, 0.027)	(-0.09, 0.184)	(-0.041, 0.099)	(-0.033, 0.243)	(-0.086, 0.093)	(0.002, 0.09)
Economic Impacts	0.006	-0.018	0.085	0.101	-0.058	-0.156	-0.026	0.018
	(0.001, 0.011)	(-0.052, 0.017)	(-0.035, 0.205)	(-0.074, 0.275)	(-0.133, 0.018)	(-0.318, 0.006)	(-0.132, 0.079)	(-0.031, 0.068)
Societal Impacts	-0.004	0.012	-0.080	0.023	0.025	0.043	-0.048	0.046
	(-0.009, 0.001)	(-0.024, 0.047)	(-0.209, 0.049)	(-0.161, 0.207)	(-0.058, 0.107)	(-0.122, 0.207)	(-0.158, 0.062)	(-0.004, 0.096)
Personal Well-Being &	0.000	0.053	**0.266****	0.030	**-0.174*****	-0.236	0.063	0.003
Community Health Impacts	(-0.005, 0.006)	(0.015, 0.09)	**(0.122, 0.411)**	(-0.161, 0.221)	**(-0.256, -0.092)**	(-0.411, -0.061)	(-0.053, 0.179)	(-0.052, 0.058)
Progress &	-0.002	**0.091*****	0.201	0.069	-0.025	0.029	-0.083	0.073
Innovation Impacts	(-0.007, 0.003)	**(0.052, 0.13)**	(0.058, 0.343)	(-0.118, 0.255)	(-0.111, 0.061)	(-0.147, 0.205)	(-0.202, 0.036)	(0.017, 0.129)
Political Impacts	-0.003	-0.040	-0.024	0.036	-0.019	-0.058	0.050	0.023
	(-0.006, 0.001)	(-0.072, -0.009)	(-0.136, 0.087)	(-0.106, 0.178)	(-0.088, 0.050)	(-0.195, 0.079)	(-0.041, 0.141)	(-0.024, 0.070)
Positive Impacts	0.001	0.042	**0.204***	0.041	-0.090	-0.167	-0.012	0.007
(Local Effects)	(-0.004, 0.005)	(0.009, 0.074)	**(0.083, 0.324)**	(-0.117, 0.200)	(-0.161, -0.019)	(-0.314, -0.020)	(-0.110, 0.086)	(-0.040, 0.053)
Negative Impacts	-0.001	0.022	-0.070	0.109	0.015	0.044	0.001	0.055
(Local Effects)	(-0.006, 0.003)	(-0.014, 0.057)	(-0.199, 0.060)	(-0.059, 0.277)	(-0.066, 0.097)	(-0.118, 0.207)	(-0.107, 0.109)	(0.002, 0.107)
Positive Impacts	0.004	0.021	**0.195***	0.193	-0.077	-0.201	-0.021	0.038
(Broad Effects)	(-0.001, 0.009)	(-0.019, 0.062)	**(0.059, 0.332)**	(0.007, 0.379)	(-0.165, 0.010)	(-0.378, -0.024)	(-0.140, 0.098)	(-0.020, 0.096)
Negative Impacts	-0.006	-0.045	-0.093	-0.038	0.029	0.167	-0.002	0.025
(Broad Effects)	(-0.010, -0.001)	(-0.080, -0.010)	(-0.223, 0.038)	(-0.205, 0.128)	(-0.057, 0.115)	(0.007, 0.328)	(-0.109, 0.105)	(-0.028, 0.079)

Table S1.5. Full Results from Regression Analysis for Constructed Indices.[f]

* = $p < 0.05$, ** = $p < 0.01$, *** = $p < 0.001$. All statistically significant results in bold.

[a] For each question, higher values represent belief that outcomes would increase.

[b] 0 = woman and 1 = man.

[c] 0 = white, 1 = non-white.

[d] 0 = Democrat, 1 = independent or other party, and 2 = Republican.

[e] 0 = 2022 and 1 = 2023.

[f] Results are for the survey-weighted linear regression model as specified in Materials and Methods, across both survey waves. P-values are Benjamini-Hochberg adjusted. IDK responses are re-coded as neutral (0). Index definitions are contained in S4 Indices definitions.

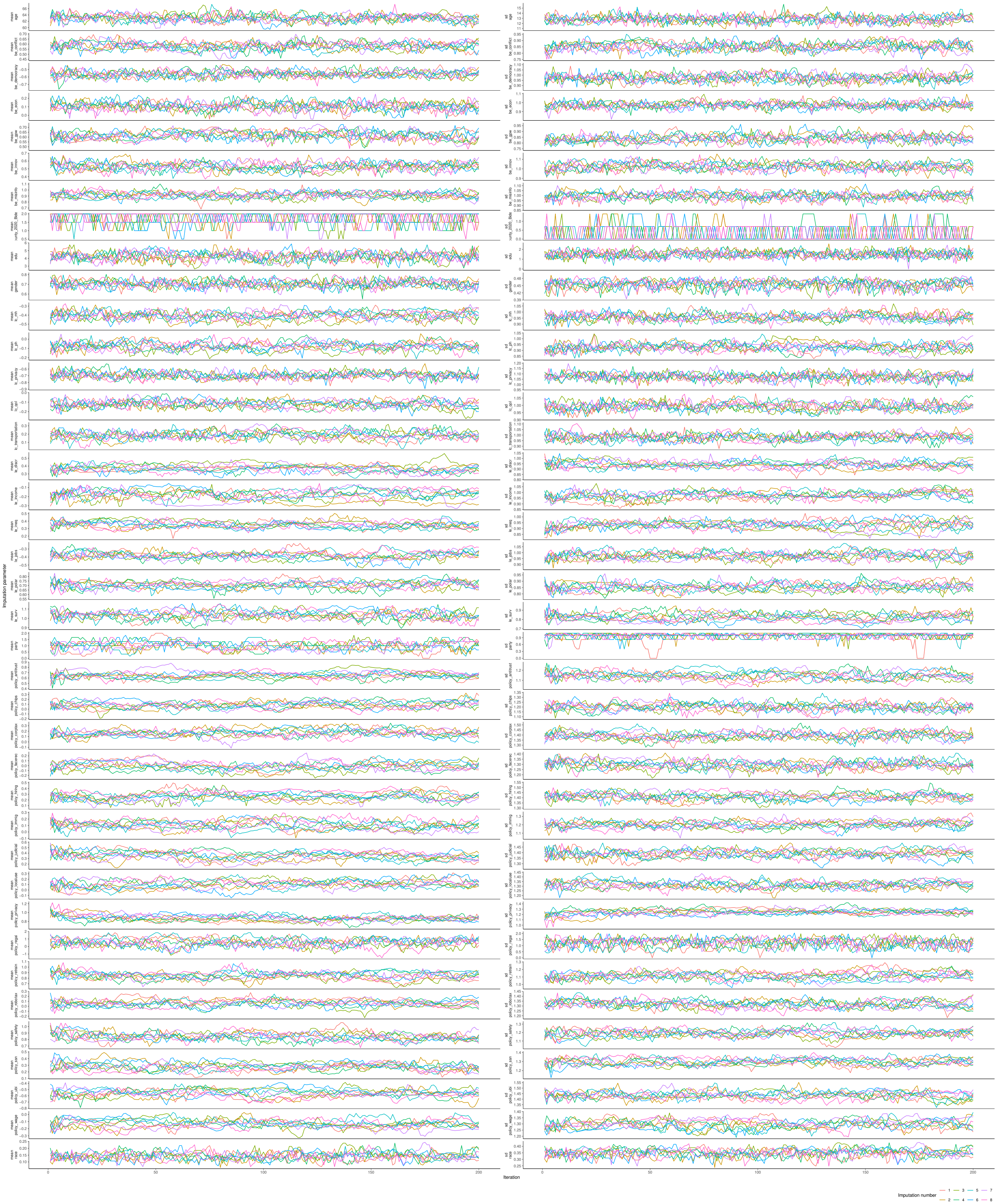

Fig S1.1. Convergence Plots for 8 Randomly Selected MICE Imputations, Across 200 Iterations Each

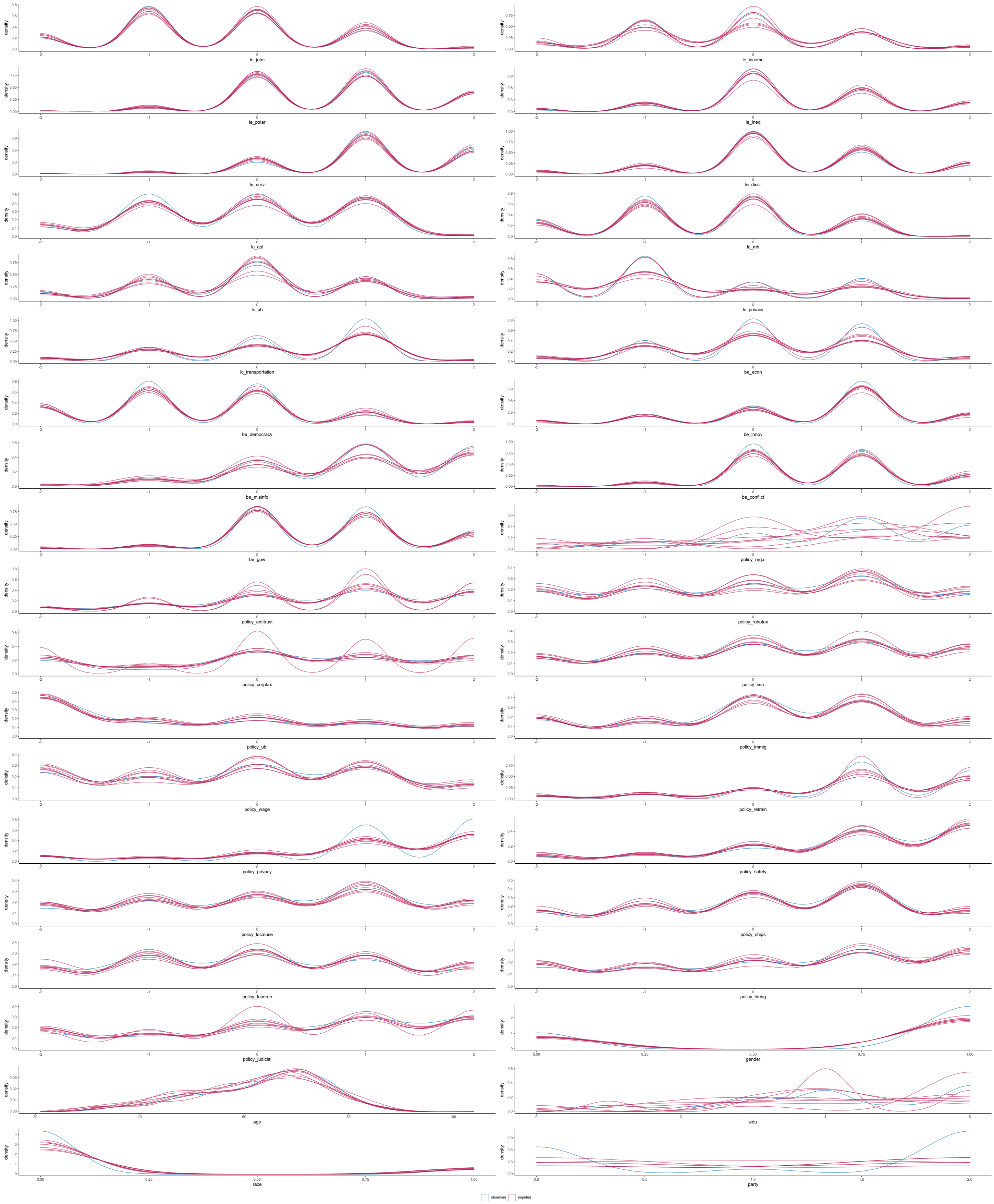

Fig S1.2. Density Plots for 8 Randomly Selected MICE Imputations
