# Supplementary material for: Local US officials’ views on the impacts and governance of AI: Evidence from 2022 and 2023 survey waves
Source: PLoS One. 2025 Oct 6;20(10):e0332919. doi: 10.1371/journal.pone.0332919 (PMC12500108; doi:10.1371/journal.pone.0332919)
Supplement: S6 — (PDF) [file pone.0332919.s024.pdf]

S6 Alternative Regression Results Tables S1.1–S1.5 contain the full results the full results of the survey-weighted linear regression model as specified in Materials and Methods, across both survey waves, but with IDK responses imputed where applicable rather than re-coded as neutral (0) as is done for the results in Differences by party, education, and gender, Differences from 2022 to 2023, and S5 Full Regression Results. P-values are Benjamini-Hochberg adjusted.

All coefficients that were statistically significant at $p < 0.05$ when IDKs are re-coded as neutral are also significant when IDKs are imputed. In addition, the model fit on IDK-imputed data sees 21 more statistically significant coefficients than the same model fit on IDKs recoded as neutral (54 vs. 33 coefficients).

Question[a]	Covariate *Age*	*Education*	*Gender*[b]	*Race*[c]	*Party*[d]	*Year*[e]	*Party ∗ Year*
**QS1: Local Effects of AI**							
Number of Jobs	0.005	0.004	0.204	0.064	-0.046	**-0.387****	0.017
	(-0.002, 0.013)	(-0.056, 0.064)	(0.005, 0.403)	(-0.19, 0.319)	(-0.163, 0.071)	**(-0.626, -0.147)**	(-0.137, 0.171)
Income Level	0.006	-0.068	0.057	-0.037	-0.039	-0.357	0.036
	(-0.002, 0.014)	(-0.13, -0.007)	(-0.169, 0.284)	(-0.31, 0.235)	(-0.175, 0.097)	(-0.623, -0.091)	(-0.132, 0.204)
Political Polarization	-0.001	0.012	-0.163	0.055	-0.006	0.165	-0.040
	(-0.008, 0.006)	(-0.041, 0.064)	(-0.35, 0.024)	(-0.186, 0.296)	(-0.115, 0.103)	(-0.057, 0.387)	(-0.183, 0.102)
Inequality	0.004	-0.001	-0.240	0.190	-0.060	0.112	-0.007
	(-0.003, 0.011)	(-0.058, 0.056)	(-0.45, -0.03)	(-0.093, 0.472)	(-0.183, 0.064)	(-0.135, 0.358)	(-0.162, 0.148)
Surveillance Level	**-0.008****	**0.066****	-0.102	0.109	0.025	0.031	-0.005
	**(-0.013, -0.002)**	**(0.024, 0.108)**	(-0.263, 0.059)	(-0.076, 0.295)	(-0.072, 0.121)	(-0.161, 0.222)	(-0.127, 0.118)
Bias & Discrimination	0.007	-0.023	-0.282	0.167	0.002	0.246	-0.046
	(-0.0, 0.015)	(-0.083, 0.036)	(-0.503, -0.062)	(-0.121, 0.454)	(-0.128, 0.131)	(-0.026, 0.518)	(-0.212, 0.12)
**QS2: Local Effects of AI**							
Quality of Life	-0.003	0.060	**0.352****	-0.148	**-0.210****	**-0.392****	0.077
	(-0.011, 0.004)	(0.006, 0.113)	**(0.156, 0.548)**	(-0.415, 0.120)	**(-0.320, -0.100)**	**(-0.637, -0.146)**	(-0.076, 0.230)
Mental Health	0.001	0.064	**0.396*****	0.135	**-0.173****	-0.288	0.107
	(-0.006, 0.008)	(0.015, 0.113)	**(0.219, 0.574)**	(-0.113, 0.383)	**(-0.278, -0.068)**	(-0.528, -0.048)	(-0.040, 0.255)
Physical Health	0.003	**0.073****	0.210	0.133	**-0.213****	-0.259	0.108
	(-0.004, 0.010)	**(0.020, 0.126)**	(0.017, 0.403)	(-0.132, 0.399)	**(-0.323, -0.103)**	(-0.495, -0.022)	(-0.042, 0.258)
Data Privacy & Security	-0.004	0.057	0.149	0.206	-0.054	-0.194	-0.044
	(-0.012, 0.004)	(0.004, 0.11)	(-0.067, 0.365)	(-0.088, 0.5)	(-0.181, 0.073)	(-0.456, 0.067)	(-0.209, 0.121)
Transportation & Infrastructure	-0.002	**0.151*****	0.197	0.077	-0.112	0.010	-0.019
	(-0.009, 0.004)	**(0.099, 0.203)**	(-0.003, 0.396)	(-0.171, 0.325)	(-0.215, -0.01)	(-0.213, 0.232)	(-0.164, 0.126)

Table S1.1. Full Results from Regression Analysis for QS1–2.[f]

* = $p < 0.05$, ** = $p < 0.01$, *** = $p < 0.001$. All statistically significant results in bold.

[a] For each question, higher values represent belief that outcomes would increase.

[b] 0 = woman and 1 = man.

[c] 0 = white, 1 = non-white.

[d] 0 = Democrat, 1 = independent or other party, and 2 = Republican.

[e] 0 = 2022 and 1 = 2023.

[f] Results are for the survey-weighted linear regression model as specified in Materials and Methods, across both survey waves. P-values are Benjamini-Hochberg adjusted. IDK responses are imputed with MICE.

Question[a]	Covariate: *Age*	*Education*	*Gender*[b]	*Race*[c]	*Party*[d]	*Year*[e]	$Party * Year$
**QS3: Broad Effects of AI**							
US Economy	0.007	0.031	0.279	0.193	-0.141	**-0.381***	-0.008
	(-0.001, 0.015)	(-0.023, 0.086)	(0.059, 0.499)	(-0.065, 0.450)	(-0.253, -0.030)	**(-0.629, -0.133)**	(-0.168, 0.153)
US Democracy	0.005	0.009	0.172	0.225	-0.109	**-0.338***	0.060
	(-0.003, 0.012)	(-0.043, 0.060)	(-0.026, 0.369)	(-0.034, 0.484)	(-0.230, 0.012)	**(-0.584, -0.092)**	(-0.091, 0.212)
US Innovation	0.002	0.056	0.199	0.082	-0.087	-0.053	-0.049
	(-0.006, 0.009)	(-0.000, 0.112)	(-0.011, 0.408)	(-0.171, 0.335)	(-0.203, 0.029)	(-0.291, 0.185)	(-0.207, 0.110)
Misinformation	-0.007	-0.006	-0.159	-0.027	0.051	**0.348***	-0.083
(News & Social Media)	(-0.014, -0.001)	(-0.057, 0.045)	(-0.350, 0.031)	(-0.275, 0.221)	(-0.071, 0.173)	**(0.102, 0.594)**	(-0.236, 0.070)
Number of Conflicts	-0.002	-0.040	**-0.296****	0.012	0.059	0.207	-0.041
	(-0.008, 0.005)	(-0.091, 0.011)	**(-0.465, -0.127)**	(-0.205, 0.228)	(-0.048, 0.166)	(-0.018, 0.432)	(-0.178, 0.096)
Probability of	-0.006	**-0.074***	-0.141	0.068	0.007	0.282	-0.036
Great Power War	(-0.012, 0.001)	**(-0.121, -0.027)**	(-0.316, 0.034)	(-0.168, 0.304)	(-0.103, 0.117)	(0.065, 0.499)	(-0.173, 0.101)

Table S1.2. Full Results from Regression Analysis for QS3.[f]

* = $p < 0.05$, ** = $p < 0.01$, *** = $p < 0.001$. All statistically significant results in bold.

[a] For each question, higher values represent belief that outcomes would increase.

[b] 0 = woman and 1 = man.

[c] 0 = white, 1 = non-white.

[d] 0 = Democrat, 1 = independent or other party, and 2 = Republican.

[e] 0 = 2022 and 1 = 2023.

[f] Results are for the survey-weighted linear regression model as specified in Materials and Methods, across both survey waves. P-values are Benjamini-Hochberg adjusted. Respondents did not have the IDK option for QS3.

Question[a]				Covariate			
	Age	*Education*	*Gender*[b]	*Race*[c]	*Party*[d]	*Year*[e]	$Party * Year$
**QS4.1: General Support for AI Regulation**							
Support AI Regulation	-0.003	-0.006	0.056	-0.259	**-0.361*****	**0.376****	0.141
	(-0.009, 0.004)	(-0.057, 0.045)	(-0.125, 0.237)	(-0.499, -0.018)	**(-0.472, -0.250)**	**(0.180, 0.572)**	(-0.001, 0.284)

Table S1.3. Full Results from Regression Analysis for Q4.1.[f]

* = $p < 0.05$, ** = $p < 0.01$, *** = $p < 0.001$. All statistically significant results in bold.
[a] For each question, higher values represent belief that outcomes would increase.
[b] 0 = woman and 1 = man.
[c] 0 = white, 1 = non-white.
[d] 0 = Democrat, 1 = independent or other party, and 2 = Republican.
[e] 0 = 2022 and 1 = 2023.
[f] Results are for the survey-weighted linear regression model as specified in Materials and Methods, across both survey waves. P-values are Benjamini-Hochberg adjusted. Respondents did not have the IDK option for QS4.1.

Question[a]	Covariate Age	Education	Gender[b]	Race[c]	Party[d]	Year[e]	Party * Year
**QS4: Policy Support**							
Stronger Anti-Trust	0.004	0.036	-0.046	-0.279	**-0.352*****	-0.012	0.071
	(-0.005, 0.013)	(-0.031, 0.104)	(-0.316, 0.224)	(-0.609, 0.052)	**(-0.488, -0.217)**	(-0.271, 0.248)	(-0.091, 0.233)
Robot Tax	-0.004	0.000	**-0.589****	-0.271	**-0.275***	0.109	0.048
	(-0.017, 0.009)	(-0.084, 0.085)	**(-0.889, -0.288)**	(-0.679, 0.138)	**(-0.441, -0.109)**	(-0.228, 0.446)	(-0.156, 0.253)
Higher Corporate Income Taxes	-0.010	0.002	-0.246	0.113	**-0.671*****	0.064	-0.019
	(-0.020, -0.000)	(-0.083, 0.086)	(-0.523, 0.031)	(-0.247, 0.473)	**(-0.831, -0.512)**	(-0.232, 0.360)	(-0.216, 0.177)
Stronger Social Safety Net	0.009	-0.008	-0.148	-0.053	**-0.560*****	0.049	0.038
	(-0.000, 0.019)	(-0.086, 0.069)	(-0.410, 0.115)	(-0.367, 0.260)	**(-0.707, -0.414)**	(-0.247, 0.345)	(-0.148, 0.224)
Universal Basic Income	**-0.014***	0.094	-0.110	0.389	**-0.850*****	-0.037	0.035
	**(-0.023, -0.005)**	(0.022, 0.166)	(-0.361, 0.141)	(0.006, 0.771)	**(-1.010, -0.690)**	(-0.351, 0.276)	(-0.142, 0.213)
Immigration Reform for AI Developers	0.005	0.001	0.208	-0.073	**-0.319****	-0.187	0.079
	(-0.005, 0.014)	(-0.074, 0.076)	(-0.074, 0.491)	(-0.427, 0.281)	**(-0.485, -0.153)**	(-0.527, 0.154)	(-0.111, 0.268)
Wage Subsidies for Wage Declines	0.000	-0.036	-0.292	-0.168	**-0.648*****	0.074	-0.020
	(-0.009, 0.010)	(-0.116, 0.044)	(-0.560, -0.025)	(-0.491, 0.155)	**(-0.803, -0.494)**	(-0.224, 0.373)	(-0.198, 0.159)
Re-Training for Unemployed	0.002	0.022	0.154	-0.050	**-0.272****	0.006	0.055
	(-0.008, 0.012)	(-0.046, 0.091)	(-0.103, 0.411)	(-0.358, 0.257)	**(-0.407, -0.137)**	(-0.258, 0.270)	(-0.113, 0.222)
Stricter Data Privacy Regulations	-0.004	**0.113***	-0.099	-0.058	-0.188	0.080	0.038
	(-0.014, 0.007)	**(0.042, 0.184)**	(-0.334, 0.136)	(-0.414, 0.297)	(-0.335, -0.041)	(-0.203, 0.364)	(-0.142, 0.217)
AI Deployment Regulations	0.000	0.041	-0.187	-0.262	**-0.298****	0.116	0.105
	(-0.009, 0.010)	(-0.033, 0.115)	(-0.437, 0.063)	(-0.602, 0.077)	**(-0.451, -0.146)**	(-0.147, 0.379)	(-0.071, 0.281)
Federal Regulations on Local Government AI	-0.005	0.043	-0.120	0.135	**-0.320****	-0.109	0.076
	(-0.016, 0.006)	(-0.044, 0.131)	(-0.397, 0.157)	(-0.226, 0.496)	**(-0.487, -0.153)**	(-0.426, 0.209)	(-0.122, 0.275)
Semiconductor & AI Hardware Subsidies	0.008	0.000	0.083	-0.133	**-0.265***	0.236	-0.048
	(-0.002, 0.017)	(-0.086, 0.086)	(-0.187, 0.352)	(-0.549, 0.283)	**(-0.425, -0.104)**	(-0.060, 0.531)	(-0.234, 0.138)
Law Enforcement Facial Recognition Ban	-0.007	0.012	-0.249	0.120	-0.117	-0.051	0.001
	(-0.018, 0.004)	(-0.070, 0.094)	(-0.574, 0.076)	(-0.296, 0.536)	(-0.302, 0.067)	(-0.400, 0.299)	(-0.210, 0.212)
Bias Audits for Hiring & Promotion AI	-0.009	0.057	-0.237	-0.028	**-0.389****	0.021	0.084
	(-0.020, 0.003)	(-0.031, 0.146)	(-0.555, 0.081)	(-0.394, 0.338)	**(-0.576, -0.203)**	(-0.328, 0.371)	(-0.129, 0.298)
Parole & Sentencing AI Regulations	-0.011	0.088	0.169	-0.019	**-0.391*****	0.275	0.059
	(-0.023, 0.001)	(0.002, 0.174)	(-0.161, 0.499)	(-0.445, 0.407)	**(-0.574, -0.208)**	(-0.062, 0.612)	(-0.151, 0.269)

Table S1.4. Full Results from Regression Analysis for QS4.[f]

* = $p < 0.05$, ** = $p < 0.01$, *** = $p < 0.001$. All statistically significant results in bold.
[a] For each question, higher values represent belief that outcomes would increase.
[b] 0 = woman and 1 = man.
[c] 0 = white, 1 = non-white.
[d] 0 = Democrat, 1 = independent or other party, and 2 = Republican.
[e] 0 = 2022 and 1 = 2023.
[f] Results are for the survey-weighted linear regression model as specified in Materials and Methods, across both survey waves. P-values are Benjamini-Hochberg adjusted. Respondents did not have the IDK option for QS4.

Question[a]	Covariate *Age*	*Education*	*Gender*[b]	*Race*[c]	*Party*[d]	*Year*[e]	*Party ∗ Year*	$Policy_{RegAI}$
**Constructed Indices**								
Policy Agreement	-0.002	0.019	-0.098	-0.083	**-0.399*****<br>**(-0.469, -0.33)**	0.106	0.056	–
	(-0.006, 0.002)	(-0.014, 0.052)	(-0.212, 0.016)	(-0.237, 0.072)		(-0.027, 0.239)	(-0.038, 0.15)	
Positive Impacts (All)	0.002	**0.052***	**0.235****	0.104	**-0.114***	**-0.275***	0.037	0.005
	(-0.003, 0.007)	**(0.018, 0.086)**	**(0.104, 0.366)**	(-0.068, 0.276)	**(-0.192, -0.036)**	**(-0.441, -0.108)**	(-0.071, 0.144)	(-0.046, 0.055)
Negative Impacts (All)	-0.003	-0.012	**-0.194***	0.061	0.051	0.201	-0.051	0.050
	(-0.007, 0.001)	(-0.043, 0.02)	**(-0.318, -0.07)**	(-0.089, 0.211)	(-0.028, 0.13)	(0.048, 0.355)	(-0.151, 0.049)	(0.0, 0.101)
Economic Impacts	0.007	-0.012	0.105	0.109	-0.071	-0.237	-0.005	0.012
	(0.001, 0.012)	(-0.051, 0.028)	(-0.04, 0.249)	(-0.085, 0.303)	(-0.158, 0.016)	(-0.426, -0.048)	(-0.127, 0.118)	(-0.045, 0.069)
Societal Impacts	-0.003	0.013	-0.147	0.014	0.015	0.090	-0.067	0.038
	(-0.008, 0.002)	(-0.025, 0.051)	(-0.285, -0.009)	(-0.186, 0.214)	(-0.075, 0.104)	(-0.09, 0.27)	(-0.187, 0.053)	(-0.016, 0.092)
Personal Well-Being &	0.000	**0.066***	**0.319****	0.040	**-0.203*****	**-0.336***	0.117	0.003
& Community Health Impacts	(-0.006, 0.006)	**(0.025, 0.107)**	**(0.159, 0.479)**	(-0.178, 0.258)	**(-0.295, -0.111)**	**(-0.54, -0.131)**	(-0.015, 0.249)	(-0.056, 0.062)
Progress &	-0.001	**0.105*****	0.209	0.128	-0.057	-0.031	-0.053	0.063
Innovation Impacts	(-0.006, 0.004)	**(0.061, 0.148)**	(0.04, 0.377)	(-0.074, 0.329)	(-0.149, 0.035)	(-0.228, 0.165)	(-0.184, 0.077)	(0.001, 0.126)
Political Impacts	-0.002	-0.047	-0.090	0.045	-0.020	-0.064	0.037	0.024
	(-0.006, 0.003)	(-0.082, -0.012)	(-0.220, 0.040)	(-0.114, 0.204)	(-0.098, 0.057)	(-0.224, 0.096)	(-0.068, 0.141)	(-0.028, 0.075)
Positive Impacts	0.001	**0.057***	**0.248****	0.048	**-0.113***	**-0.246***	0.030	0.000
(Local Effects)	(-0.004, 0.006)	**(0.020, 0.093)**	**(0.111, 0.385)**	(-0.137, 0.234)	**(-0.193, -0.033)**	**(-0.420, -0.071)**	(-0.082, 0.143)	(-0.051, 0.052)
Negative Impacts	-0.000	0.016	**-0.191***	0.121	0.032	0.119	-0.049	0.062
(Local Effects)	(-0.005, 0.005)	(-0.025, 0.056)	**(-0.332, -0.050)**	(-0.058, 0.300)	(-0.057, 0.121)	(-0.056, 0.294)	(-0.167, 0.069)	(0.004, 0.119)
Positive Impacts	0.004	0.035	**0.223***	0.209	-0.107	**-0.289***	0.009	0.031
(Broad Effects)	(-0.002, 0.010)	(-0.010, 0.080)	**(0.058, 0.389)**	(0.005, 0.414)	(-0.205, -0.009)	**(-0.495, -0.084)**	(-0.127, 0.145)	(-0.033, 0.095)
Negative Impacts	-0.005	-0.037	**-0.205***	-0.003	0.044	**0.290***	-0.065	0.025
(Broad Effects)	(-0.010, 0.000)	(-0.076, 0.001)	**(-0.347, -0.063)**	(-0.188, 0.181)	(-0.053, 0.140)	**(0.106, 0.474)**	(-0.184, 0.053)	(-0.033, 0.082)

Table S1.5. Full Results from Regression Analysis for Constructed Indices.[f]

* = $p < 0.05$, ** = $p < 0.01$, *** = $p < 0.001$. All statistically significant results in bold.

[a] For each question, higher values represent belief that outcomes would increase.

[b] 0 = woman and 1 = man.

[c] 0 = white, 1 = non-white.

[d] 0 = Democrat, 1 = independent or other party, and 2 = Republican.

[e] 0 = 2022 and 1 = 2023.

[f] Results are for the survey-weighted linear regression model as specified in Materials and Methods, across both survey waves. P-values are Benjamini-Hochberg adjusted. IDK responses are imputed with MICE. Index definitions are contained in S4 Indices definitions.
