# Supplementary material for: Local US officials’ views on the impacts and governance of AI: Evidence from 2022 and 2023 survey waves
Source: PLoS One. 2025 Oct 6;20(10):e0332919. doi: 10.1371/journal.pone.0332919 (PMC12500108; doi:10.1371/journal.pone.0332919)
Supplement: S7 — (PDF) [file pone.0332919.s025.pdf]

S7 Survey experiment We included a survey experiment to test whether respondents' beliefs on the effects of AI would changed based on a prompt about the long-term effects of AI. Respondents were randomly assigned to receive the prompt below:

> Think about children born in your community today. According to current average life expectancy rates, some of them might be alive in the year 2100.

All respondents were then presented with Q4.2: "Do you think that AI will have an overall positive or negative effect on the US from now until 2100?" Respondents were then asked to explain their response in a free-text field. The response scale was a 5-point Likert scale ranging from very negative (-2) to very positive (2); respondents were permitted to respond with the IDK option.

Relative frequencies for responses to Q4.2 are shown in Table S1.1.

We estimated the average treatment effect (ATE) of the experimental treatment above using the following survey-weighted linear regression model:

$$\begin{aligned} y = \mathbb{1}_{Treatment} + gender + age + edu + race + party + year_{2023} + party * year_{2023} \\ + gov_{municipality} + gov_{county} + college + pop + Biden \end{aligned}$$

where y is the response to Q4.2, $\mathbb{1}_{Treatment}$ is an indicator variable for whether the respondent was treated, and the other variables are as defined in S2 Variable definitions. As with the main regression results presented in S5 Full Regression Results and S6 Alternative Regression Results, we estimate the ATE on both 1) an imputed dataset where the IDK values are set to neutral and not imputed, and 2) an imputed dataset where the IDK values are treated as missing and imputed.

As is shown in S1.1, the experimental treatment was not found to have a statistically significant effect at the 5% level. These results were not adjusted for multiple comparisons.

	ATE	Standard Error	P-Value	95% CI
Neutral-Coded IDKs	0.120	0.078	0.127	(-0.034, 0.273)
Imputed IDKs	0.126	0.078	0.107	(-0.027, 0.0230)

Table S1.1. ATE Regression Results for Experimental Treatment (Q4.2)

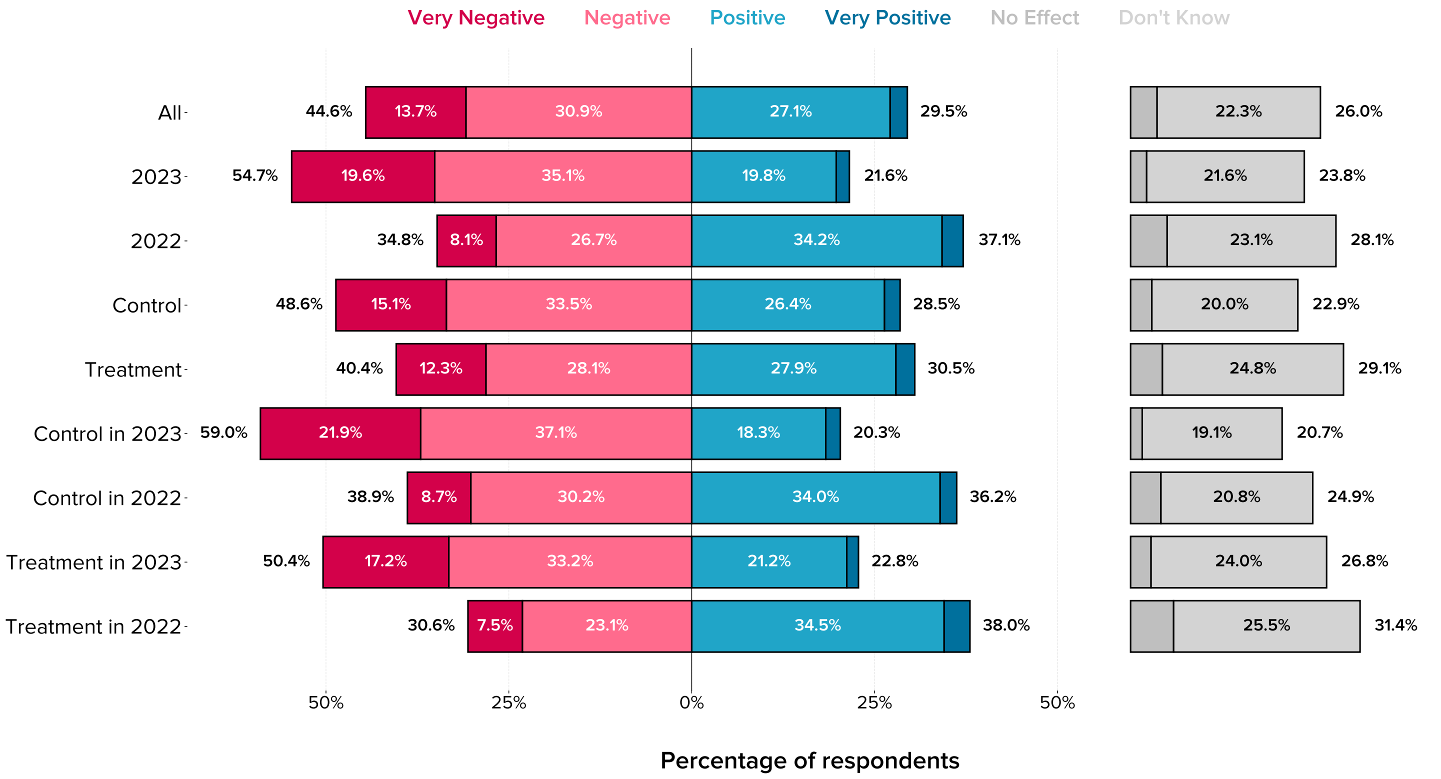

Fig S1.1. Local US officials' responses to the question "Do you think that AI will have an overall positive or negative effect on the US from now until 2100?", with overall support and segments by treatment group and year.[a]

[a] The figure shows unweighted relative frequencies across both survey waves.
