# Supplementary material for: Local US officials’ views on the impacts and governance of AI: Evidence from 2022 and 2023 survey waves
Source: PLoS One. 2025 Oct 6;20(10):e0332919. doi: 10.1371/journal.pone.0332919 (PMC12500108; doi:10.1371/journal.pone.0332919)
Supplement: S8 — (PDF) [file pone.0332919.s027.pdf]

S8 Sample representativeness Same representativeness statistics relative to Census statistics are presented below in Tables S1.1 and S1.2.

Table S1.1. Sample representativeness of county, municipality, and township officials in the 2022 survey wave

Category	Area Characteristics	Sample Median	Population Median
County Officials	Proportion Urban	0.52	0.40
	Proportion College-educated	0.21	0.19
	Population Size	42,360	25,750
	Democratic Vote Share 2020	0.36	0.30
Municipality Officials	Proportion Urban	0.99	0.98
	Proportion College-educated	0.25	0.21
	Population Size	5,030	4,180
	Democratic Vote Share 2020	0.42	0.40
Township Officials	Proportion Urban	0.09	0.01
	Proportion College-educated	0.27	0.22
	Population Size	3,750	2,680
	Democratic Vote Share 2020	0.48	0.39

Table S1.2. Sample representativeness of county, municipality, and township officials in the 2023 survey wave

Category	Area Characteristics	Sample Median	Population Median
County Officials	Proportion College-educated	0.22	0.19
	Population Size	42,360	25,750
	Democratic Vote Share 2020	0.35	0.30
Municipality Officials	Proportion College-educated	0.25	0.21
	Population Size	5,080	4,180
	Democratic Vote Share 2020	0.43	0.40
Township Officials	Proportion College-educated	0.27	0.22
	Population Size	3,900	2,680
	Democratic Vote Share 2020	0.48	0.39
